# Better Fitness in Captive Cuvier’s Gazelle despite Inbreeding Increase: Evidence of Purging?

**DOI:** 10.1371/journal.pone.0145111

**Published:** 2015-12-17

**Authors:** Eulalia Moreno, Javier Pérez-González, Juan Carranza, Jordi Moya-Laraño

**Affiliations:** 1 Estación Experimental de Zonas Áridas (CSIC), Dept. Ecología Funcional y Evolutiva, Ctra. de Sacramento s/n, E-04120, La Cañada de San Urbano, Almería, Spain; 2 Ungulate Research Unit, Cátedra de Recursos Cinegéticos y Piscícolas, Universidad de Córdoba, E-14071, Córdoba, Spain; 3 Guardería Rural, Mancomunidad Integral de Municipios Centro, E-06810, Calamonte, Badajoz, Spain; University of Arkansas, UNITED STATES

## Abstract

Captive breeding of endangered species often aims at preserving genetic diversity and to avoid the harmful effects of inbreeding. However, deleterious alleles causing inbreeding depression can be purged when inbreeding persists over several generations. Despite its great importance both for evolutionary biology and for captive breeding programmes, few studies have addressed whether and to which extent purging may occur. Here we undertake a longitudinal study with the largest captive population of Cuvier's gazelle managed under a European Endangered Species Programme since 1975. Previous results in this population have shown that highly inbred mothers tend to produce more daughters, and this fact was used in 2006 to reach a more appropriate sex-ratio in this polygynous species by changing the pairing strategy (i.e., pairing some inbred females instead of keeping them as surplus individuals in the population). Here, by using studbook data we explore whether purging has occurred in the population by investigating whether after the change in pairing strategy a) inbreeding and homozygosity increased at the population level, b) fitness (survival) increased, and c) the relationship between inbreeding and juvenile survival, was positive. Consistent with the existence of purging, we found an increase in inbreeding coefficients, homozygosity and juvenile survival. In addition, we showed that in the course of the breeding programme the relationship between inbreeding and juvenile survival was not uniform but rather changed over time: it was negative in the early years, flat in the middle years and positive after the change in pairing strategy. We highlight that by allowing inbred individuals to mate in captive stocks we may favour sex-ratio bias towards females, a desirable managing strategy to reduce the surplus of males that force most zoos to use ethical culling and euthanizing management tools. We discuss these possibilities but also acknowledge that many other effects should be considered before implementing inbreeding and purging as elements in management decisions.

## Introduction

Captive breeding is a valuable tool for the preservation of endangered species, and in some instances it may turn out to be the only possible way to avoid total extinction. Most captive breeding programmes maintain a small population of breeders that usually begins with very few founders. Under such conditions, management decisions are rooted in the aim of preserving genetic diversity and avoiding inbreeding to reduce the deleterious effects of an increased level of homozygosity in the population: the so named “inbreeding depression” or reduced fitness of offspring due to mating between relatives. Since Darwin’s experiments on selfing in plants [1], inbreeding depression has been documented in a wide array of species [2–4]. However, various authors failed to find the predicted decrease in fitness after inbreeding [5–9], so that the deleterious effects of inbreeding are not universal and straightforward (see also [10]). Inbreeding depression may result from any inherent advantage of heterozygotes over homozygotes (the overdominance hypothesis), although increasing evidence suggests that the most common cause is the unmasking of recessive deleterious alleles in homozygous genotypes (the dominance or partial dominance hypothesis [11–13]). Although a process of inbred mating can reduce the fitness of the individuals and the population, it also enhances selection against recessive deleterious alleles as they are expressed in homozygosity [14–16], and can therefore purge them from the population even to the point of counteracting the negative effects of genetic load caused by drift and inbreeding [17–18].

The phenomenon of purging implies the existence of different temporal phases across generations [9]: in an earlier stage we expect depression (inbreeding increases the probability of homozygosity for deleterious recessive alleles), and it is then when natural selection can act against deleterious alleles that may thereby reduce their frequency [19]. Thereafter, the genetic load may have been reduced until a point in which inbred individuals do not show lower fitness. Purging may occur in small captive populations [17] but it can also be the reason why inbreeding depression is not evident in many current endangered populations in islands [20]. Under conditions of partial or incomplete dominance between alleles, the removal of deleterious recessive alleles by purging can even lead to increased fitness of individuals or populations after inbreeding events due to the added effects of positively-selected homologous-alleles [12]. However, in natural populations, the effectiveness of purging and its beneficial effects on fitness may depend on the rate of inbreeding, so that only a slight inbreeding over a rather long period of time could remove deleterious recessive alleles without harmful effects on population viability [21].

Different approaches have been used to study inbreeding depression and purging. Computer simulations [16, 22–23] have been relevant in testing hypotheses explaining the underlying genetic mechanism of inbreeding depression and purging. In many occasions laboratory animals have been used as pilot species for evaluating the outcomes of inbreeding and for testing predictions [9–10, 17, 21, 24]. The literature published so far shows contrasting results on how purging events can affect population viability [9, 17, 21] so that it is uncertain to which extent the potential benefits of purging could be a useful tool in managing captive populations.

Zoo studbooks containing detailed pedigree of species kept in captivity have also been used to investigate the consequences of inbreeding on fitness related traits [8]. Studbooks are valuable as generally contain both long-term pedigrees as well as relevant associated data of the captive stock of interest for conservation programmes (e.g., dates of birth, and death [25]). They also provide information to genetically manage captive populations through a variety of approaches that account for genetic variables (mean inbreeding, mean kinship, gene diversity).

In this paper we undertake a longitudinal study to investigate the relationship between inbreeding and juvenile survival, an obvious key fitness trait, in a captive population of Cuvier’s gazelle (*Gazella cuvieri* [26]) managed under a European Endangered Species Programme (EEP) since 1975. Previous studies in this population demonstrated the existence of inbreeding depression in some fitness traits, whose values declined in parallel to an increase of inbreeding. Alados and Escós [27] showed that high levels of inbreeding were linked to low birth weight, which reduced juvenile survival. Roldan et al. [28] reported that among males of *Gazella cuvieri*, the inbreeding coefficient showed a strong negative relationship with ejaculate quality. Cassinello [29] found that survival to one month in males and to sexual maturity in both sexes was significantly higher in less inbred individuals of Cuvier’s gazelles. However, later on in the breeding programme some authors failed to find inbreeding depression in this same population. Ibáñez et al. [30] did not find any inbreeding effect on body weight. Similarly, Ruiz-López et al. [31] and Ibáñez et al. [32] showed that neither mother nor offspring inbreeding had any effect on juvenile mortality.

These seemingly contradictory results within the same captive population, reported in different time periods, are certainly intriguing and deserve a reappraisal. Inbreeding avoidance is a common practice in captive breeding programs due to the potentially negative fitness consequences of inbreeding [8, 33–34]. However, if after a number of generations of inbreeding purging is demonstrated to take place in captive populations, it might be advantageous to favor inbreeding, at least in some individuals. In polygynous species this would be done when arranging pairings, and would serve as a way of solving the organizational and even ethical (if culling is the alternative option) problems posed to captive breeders of where and how to keep the surplus individuals, mainly males [35–36].

Here we integrate studbook information and molecular techniques to analyze the fitness consequences of inbreeding in the captive population of Cuvier’s gazelle maintained at La Hoya Experimental Field Station (Almería, Spain) for more than 35 years. Its EEP began in Almería from four founders: 1 male and 3 females [37]. In this extremely bottlenecked population it was shown that more inbred mothers had a higher probability of producing daughters [38], which was used by EEP’s manager (EM) to try to reach a more appropriate sex-ratio in the captive population of this polygynous species. Obviously, an increase of inbreeding occurred as a result of the management to increase female bias in the population [39]. This provided us with the unique opportunity to explore the possibility that deleterious alleles had been purged from this captive population. We predicted that after the change in paring strategy a) inbreeding and homozygosity would increase at the population level, b) fitness (juvenile survival, a key fitness-linked trait) would increase, and c) the relationship between inbreeding and juvenile survival would be positive despite an increase in inbreeding and homozygosity.

## Material and Methods

### Study captive population

Cuvier’s gazelle is a Sahelo-Saharan endangered species whose populations have steeply declined since the 1950’s apparently due to excessive hunting and habitat degradation in their range (Morocco, Tunisia, Algeria [40]). This polygynous species is medium-sized and sexually dimorphic, with adult males 24% heavier than adult females (average body mass of adult females: 26 kg; adult males: 34 kg) [37]. Females are fertile at about 8–9 months and males at 12–13. Gestation is about 5.5 months, twins representing up to 39% of births [37]. The captive population within its EEP is about 160–170 individuals distributed at four zoological institutions [41], the biggest population being that housed at La Hoya, with a mean of about 140–150 individual in the last 5–10 years. Our study has been carried out in this latter population, which is currently the only breeding population within the EEP (53 males: 95 females by 31 December 2011) [41].

### Management of the captive population

Since the captive population of Cuvier’s gazelle was established at La Hoya Field Station, most animals have been included in breeding herds comprised of one adult male (included in the breeding herd in October-November) and a group of five to eight adult females. Young males are removed from breeding herds when they become 7–8 months old and form bachelor groups (see detailed husbandry guidelines and different types of enclosures in [37]). Due to space limitations existing in La Hoya as well as the high proportion of twins in Cuvier’s gazelle, since 2004 only 4–6 breeding herds are formed per breeding season, the rest of the animals being kept in bachelor, single sex enclosures. Pairing strategies are set up by using the SPARKS software programme [42] following the criteria of minimizing inbreeding in descendants. Highly inbred animals are generally not used as breeders as their potential offspring would have an inbreeding coefficient higher than the current day average of mean inbreeding values [43]. These highly inbred individuals represent for this and other EEPs the surplus stock: animals that are no longer needed for the goals of the EEP, although they can be (and certainly are) used for exhibition and educational purposes in zoos participating in the EEP.

Since the late 90s the sex ratio of the Cuvier’s population was rather balanced. However, Moreno et al. [38] observed that the higher the inbreeding coefficient of the mother the higher the probability of producing daughters, which could be advantageous to obtain a female biased population (see also [39]), thereby both, increasing the productivity of the captive stock of this polygynous species, and decreasing the number of born males and thus helping the always problematic management of surplus males. Therefore, in agreement with other suggestions for manipulating the sex-ratio in endangered captive populations [44–45], in autumn 2006 the strategy for pairing arrangements was slightly changed. Considering the criterion of minimizing the inbreeding of the future offspring and using the SPARKS software [42] breeding herds were set up as follows: 1 male and 4–6 females were chosen to breed and then 1–2 females with the highest inbreeding were also included in these breeding enclosures. These later highly inbred females were not randomly chosen but were rather chosen by trying to minimize the expected inbreeding of their offspring considering the inbreeding coefficient (relatedness) of the male they would mate with. As a consequence of the later criterion, there was no correlation between the inbreeding coefficient of the mothers and their offspring (r = -0.05, ns; N = 129). This change in the pairing strategy allowed us to use as breeders females that otherwise would be considered as “surplus”, non-breeder females within the EEP. By doing this we expected to increase the production of daughters born in our population and to reduce the number of future surplus males. As a consequence of the change in the pairing strategy, 94 and 35 calves were born from highly and low inbred females during 2007–2011 respectively. Juvenile survival for both groups was similar: 83.0% for the former and 88.6% for the later. Inbreeding coefficient for the descendants of these two groups of females did not differ either (mean ± SD = 0.250 ± 0.047 for calves born from highly inbred females *vs*. mean ± SD = 0.252 ± 0.061 for calves born from low inbred females; Mann-Whitney U tests, p>0.1). Neither did it individual heterozygosity, measured as standardized heterozygosity (SH; [46]) (SH ± SD = 0.772 ± 0.256; N = 5; for descendants from highly inbred mothers *vs*, SH ± SD = 0.949 ± 0.183; N = 5; for descendants from low inbred mothers; Mann-Whitney U tests, p>0.1).

As expected, since spring 2007 sex ratio in the captive Cuvier’s gazelle population progressively changed to female biased [41]. However, even though offspring born from highly inbred mothers did not have higher inbreeding coefficients than the mean individual inbreeding coefficient of the other offspring born in the same breeding season this change in the pairing strategy could also increase the mean inbreeding at the population level [47–48]. Although an increase of inbreeding has occurred in La Hoya population since it was established in 1975 [30], we tested whether the radical change in the pairing strategy performed in 2006 would substantially increase the rate of inbreeding in the captive population. Thus, this situation provides us with the opportunity of monitoring possible effects of mothers' inbreeding on offspring fitness by comparing the relationship between inbreeding and juvenile survival before and after the above-mentioned change in management in the studied captive population.

It might be argued that having more females than males is good for management in this population but it also decrease genetic variation faster than having equal numbers of the two sexes. Although unequal sex ratio decreases Ne and increases the loss of genetic variation, the effect of estimated Ne on actual transmission of genetic variability is also mediated by other factors, including the distribution of mating success and generation time. Hence, when the distribution of mating success is skewed, like in harem-defense polygyny (the case of Cuvier’s gazelle), the actual number of males contributing to the next generation might become lower than expected by Ne. But in captive breeding, by contrast, managers may tend to balance mating success for all available males by pairing them in several breeding seasons, a practice normally applied to Cuvier’s gazelle thanks to both, its short generation time and long lifespan [37]. By doing so, the distribution of mating in captivity is likely more even among males than expected in the wild for the same sex ratio. This means that some sex-ratio bias in the captive stock may not differ too much from a more even sex-ratio situation in the wild under polygynous mating.

### Inbreeding coefficient and juvenile survival

Data for 631 Cuvier’s gazelles housed currently or historically at La Hoya Field Station were used in the analyses. These data were obtained from the studbook (S1 File [41]) which includes records of all individual Cuvier’s gazelle in the EEP population. It is regularly updated using the studbook database software SPARK [42]. Data from SPARKS are exported to the studbook analysis software PMx [49] for calculation of inbreeding coefficients. In this population, whose percentage of pedigree known is 100%, the increase in inbreeding coefficient is not linear across generations [30] but rather depends on pedigree depth (see [50–51] for similar findings in other populations). Results presented in this paper are based on studbook data up to December 31^st^ 2011 [41].

We were interested in the relationship between inbreeding and juvenile survival at 14 days of age, survival being a target phenotypic trait related to fitness widely used in inbreeding depression studies (see e. g. [3]). This age represents the critical period of mortality for Cuvier’s gazelle in captivity, as above 80% of calves that die, do so below that age [32].

### DNA sampling and genetic analyses

To study genetic effects at the population level after the change in management, we compared heterozygosity before and after the change. We obtained DNA samples from skins in EEZA-CSIC scientific collections which are stored in 95% ethanol. Ear skins samples are routinely taken from all individuals born in La Hoya during the process of ear tagging for individual identification, these samples being all included in the EEZA-CSIC scientific collections. Skins samples of 39 randomly chosen individuals were used in the analyses. The birth year of these individuals ranged from 1984 to 2011: 29 of them before and 10 after the management change in 2006. Although the proportion of individuals sampled for genotyping after 2006 was relatively small (10/39 = 0.256), this proportion was similar to the proportion of individuals analyzed for inbreeding and survival analyses in the same period (129/631 = 0.204; see below). No skin samples were available in the collection for individuals born earlier than 1984. Genomic DNA was purified by proteinase K digestion and salting out procedure. Fifteen fluorescent-labeled microsatellite markers were initially tested: CelJP15, MM12, OarFCB304, OarFCB193, ETH225, BM1818, OarFCB5, TGLA53, CelJP38, CSSM19, RME25, CSSM22, CSSM43, OarCP26 and CSPS115 [52–59]. There markers were initially developed for species such as sheep, cattle or red deer. Some of the analyzed markers were discarded either due to the absence of polymorphism or due to difficulties in amplifying or scoring. Finally, 8 microsatellite markers were chosen to genetically type the samples: CSPS115 (5 alleles in our sample), CSSM22 (4 alleles), CSSM43 (5 alleles), ETH225 (7 alleles), OarFCB193 (6 alleles), OarFCB304 (8 alleles), CelJP15 (3 alleles) and MM12 (2 alleles). After polymerase chain reaction (PCR), we used ABI 3130 DNA sequencer and GENEMAPPER 3.7 software (Applied Biosystems) to estimate allele sizes.

We used GENEPOP, version 4.3 [60] to assess the presence of linkage disequilibrium between loci. In our sample, significant linkage disequilibrium was detected between CSPS115 and CSSM43 loci. We conducted genetic analyses after removing the CSSM43 locus. Therefore, genetic effects were assessed by using 7 mixrosatellites markers (CSPS115, CSSM22, ETH225, OarFCB193, OarFCB304, CelJP15 and MM12; genotypes in S2 File). Results were very similar after removing CSPS115 instead of CSSM43. Observed (*H*_*o*_) and expected (*H*_*e*_) heterozygosities were obtained using the software GENETIX, version 4.05 [61]. Heterozygosity before and after 2007 was compared by using the F_is_ index as the difference between expected and observed heterozygosity divided by the expected one [62]. We assessed whether F_is_ indexes were significantly different from 0 by using 10,000 permutations in GENETIX.

### Statistical analyses

In addition to heterozygosity, we compared individual inbreeding and juvenile survival at age of 14 days before and after 2007 (the first generation after the pairing strategy changed was born in spring 2007; N = 502, N = 129 respectively). In order to increase the power of our analyses, we performed permutation tests in which we obtained samples by permuting the original dataset 10,000 times. In each sample a pivotal statistic was estimated and compared to the observed value in the original dataset [63]. P values were obtained by counting the proportion of times the value of the observed statistic was lower or higher than the statistic resulting in the samples. Inbreeding before and after 2007 was compared by T-Student tests, so a *t* statistic was used as pivotal for bootstrap analyses; juvenile survival was compared by a logistic regression, so a *z* statistic was used as pivotal for permutation tests.

We assessed how the relationship between inbreeding and juvenile survival changed through time. The data were analyzed fitting Generalized Linear Mixed Models (GLMM) using the function “glmer” in lme4 (package version 1.1.9) within R [64]. Juvenile survival (estimated as explained above) was the dependent variable. Birth year, inbreeding and their interaction were included in the model as fixed factors. In all the analyses, offspring were used as statistical units. As some of them belong to the same mother in different breeding seasons, they are not totally independent. Hence, mother identity was introduced as a random factor in the analyses. Furthermore, we know that this dam component affects offspring survival [65], thus making its inclusion necessary. Twins tend to be lighter at birth, and therefore, their mortality tends to be higher than for singletons [66]. Thus, we also included twin status (single birth *vs*. being born with a twin) as an additional fixed factor in the model. Because we knew beforehand that the evolution of inbreeding in the studied population was non-linear [30] we fitted a spline with three knots (function “bs”) around inbreeding to investigate the relationship between inbreeding and juvenile survival. The percentage of variance explained by mother random effect was calculated by providing marginal and conditional R^2^ values respectively for fixed and overall effects [67]. Confidence intervals for dam effects were estimated using the function “confint.merMod” in package “lme4”. To plot the main results of the model we fitted the same model in “glmer” (“lme4” package version 1.1.9) and used the package “effects” (version 3.0.4), which is particularly suitable when complex interactions are involved [68].

## Results

### Inbreeding coefficient and heterozygosity

**Fig 1:**
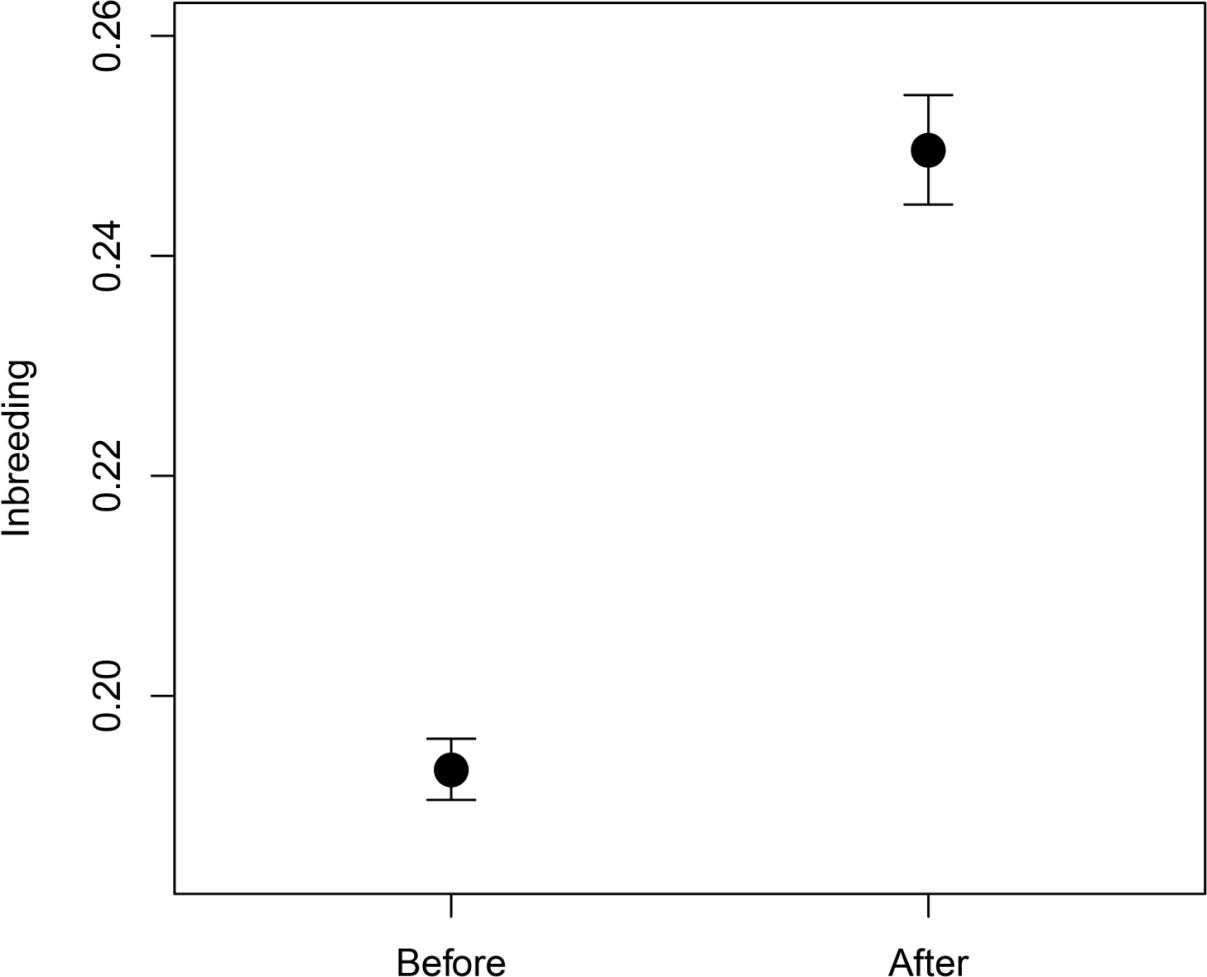
Differences in inbreeding. Inbreeding before and after the pairing strategy change occurred in 2006. Figure shows means and standard errors.

Change in pairing strategy carried out in 2006 in La Hoya increased the Cuvier’s gazelle inbreeding coefficient at the population level (mean ± SE = 0.193 ± 0.003, before change; mean ± SE = 0.250 ± 0.005, after change; permutation test, observed *t* = -9.881, 10,000 samples, p < 0.00001; “Fig 1”). The change in pairing strategy also produced a reduction in heterozygosity (*H*_*o*_ = 0.70, *H*_*e*_ = 0.63, F_is_ = -0.11 before change; *H*_*o*_ = 0.57, *H*_*e*_ = 0.64, *F*_*is*_ = 0.11 after change). Before change F_is_ value was significantly lower than 0 (10,000 permutations, p < 0.01) while it tended to be higher than 0 (10,000 permutations, p = 0.08) after the change. Therefore, the population went from having a level of heterozygosity above Hardy-Weinberg equilibrium before management practices changed, to a level lower than expected under equilibrium after the change in management.

### Effects on juvenile survival

**Fig 2:**
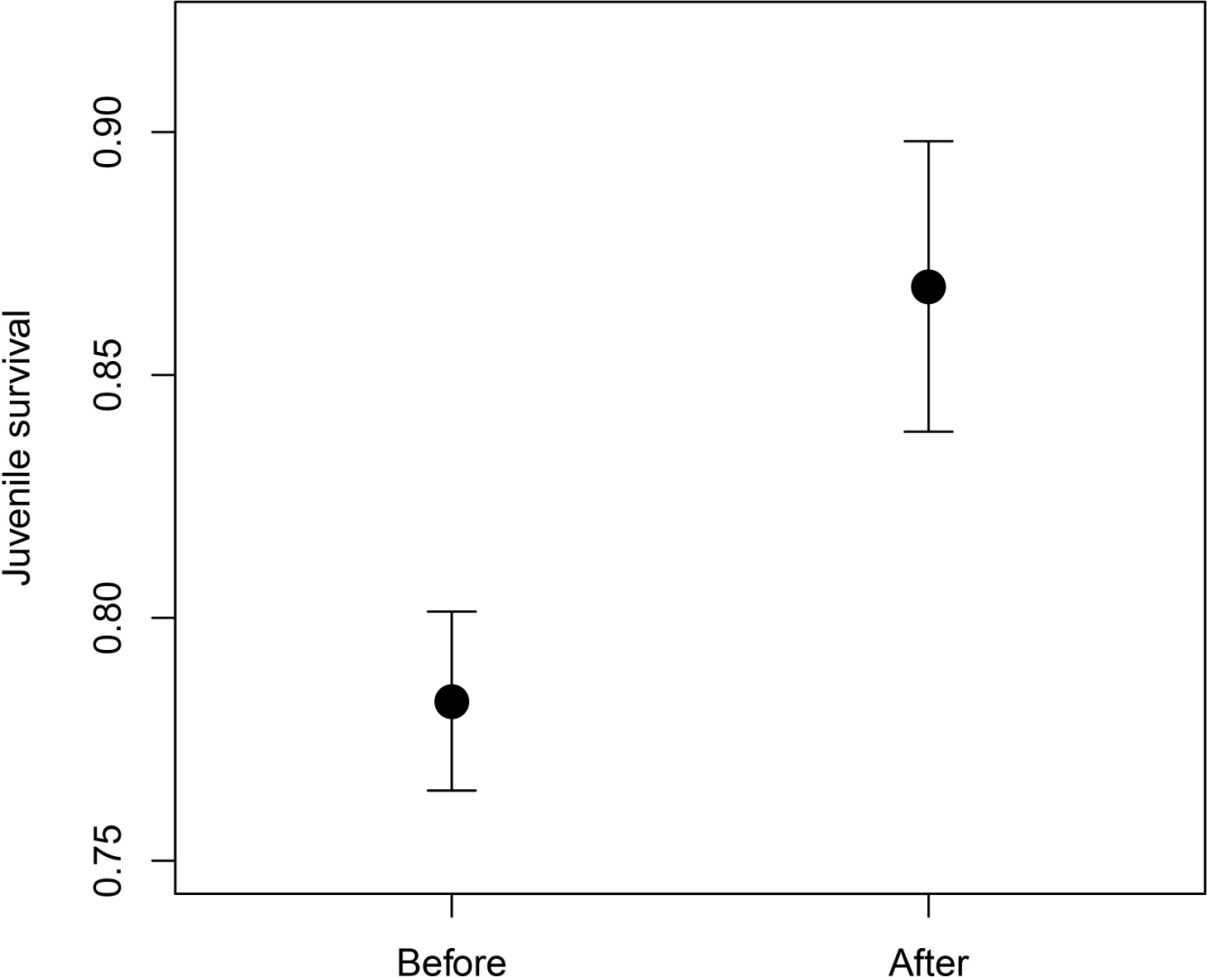
Differences in juvenile survival. Juvenile survival before and after the pairing strategy change. Juvenile survival is a dichotomous variable in which 0 indicates dead individuals and 1 indicates surviving individuals. Figure shows mean and standard error.

Juvenile survival was significantly higher after the pairing strategy was changed in 2006 despite the increase in inbreeding (juvenile survival: 0 dead individuals, 1 surviving individuals; mean ± SE = 0.783 ± 0.018, before change; mean ± SE = 0.868 ± 0.030, after change; permutation test, observed *z* = 2.138, 10,000 samples, p = 0.013; “Fig 2”).

**Fig 3:**
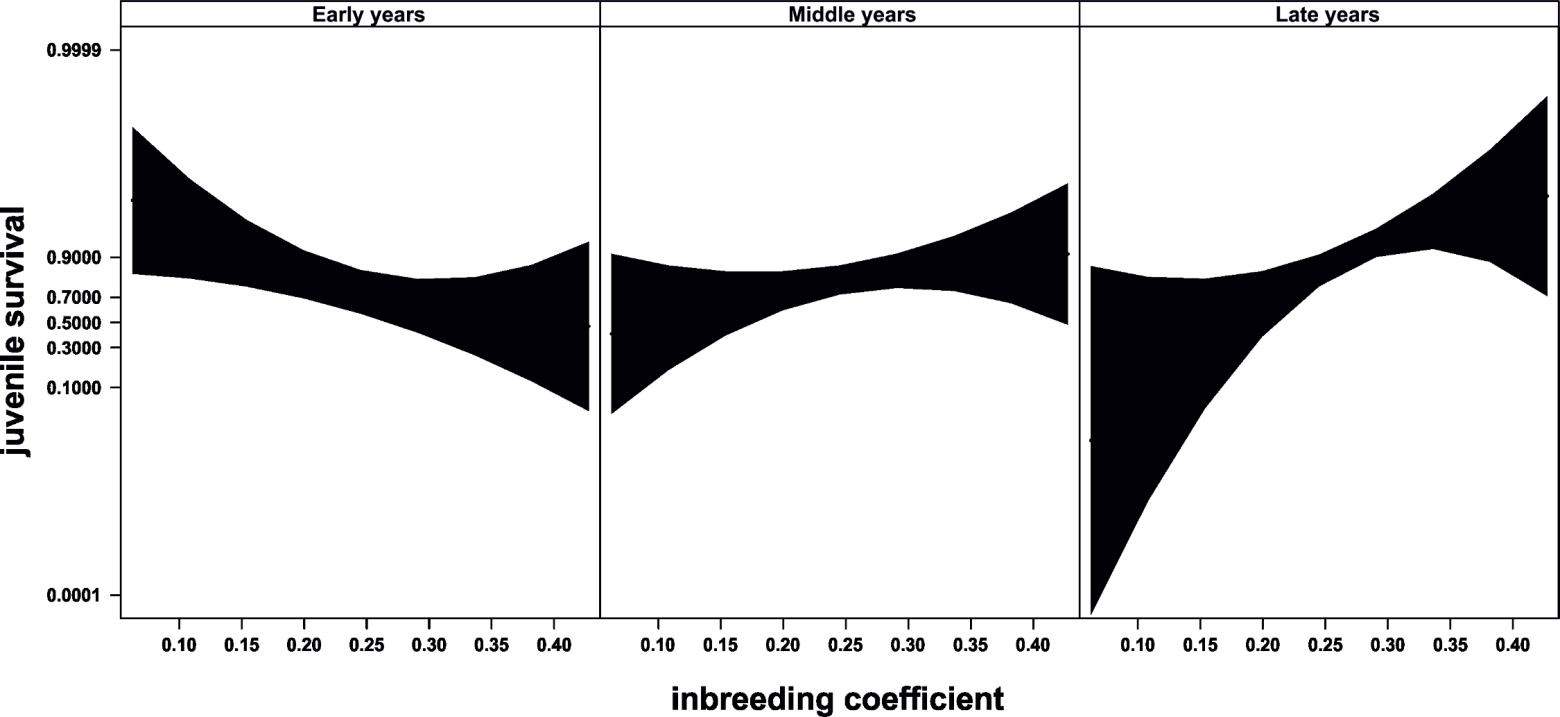
Evolution of juvenile survival through time. Relationship between inbreeding and juvenile survival through time. Juvenile survival is a dichotomous variable in which 0 indicates dead individuals and 1 indicates surviving individuals. Figure shows predicted values and 95% confidence bands. Time progresses from left to right, so the left graph shows the relationship between inbreeding and juvenile survival centred in year 1981, while the right graph shows this relationship centred in year 2006 when a major change in pairing management occurred. Graphs were produced with library “effects” [68], which uses the estimates of the effects from GLMMs to predict the values across the entire expand of the explanatory variables. Confidence bands are calculated from standard errors estimated at each of three levels of inbreeding (0.0625, 0.2451 and 0.4277) within each of the time periods.

The relationship between inbreeding and juvenile survival changed through time in the breeding programme (Table 1, “Fig 3”). At the beginning, higher levels of inbreeding were associated with low juvenile survival (“Fig 3” left). However, this trend reverted as time elapsed, and higher inbreeding was positively associated to higher juvenile survival during the years after the change in pairing strategy (“Fig 3” right). Additionally, the random effect of mothers reached significance (sd = 1.23, lower 95% CI = 0.81, upper 95% CI = 1.77) and explained most of the variance (marginal-R^2^ = 0.055; conditional-R^2^ = 0.354), indicating either maternal or genetic effects on offspring survival. Twin status was not significant, nor any of its 2-way or 3-way interactions with the other terms (all P>0.3), and it was therefore discarded from the final model.

**Table 1:** GLMM model using the library “lme4”.

	estimate	se	z	P
Intercept	6.381	2.247	2.840	0.0045
Birth Year	-0.451	0.219	-2.059	0.0395
Inbreeding 1	-10.91	4.839	-2.254	0.0242
Inbreeding 2	-1.299	2.730	-0.476	0.6341
Inbreeding 3	-7.735	3.173	-2.438	0.0148
Birth Year * Inbreeding 1	1.035	0.446	2.323	0.0202
Birth Year * Inbreeding 2	0.042	0.179	0.235	0.8145
Birth Year * Inbreeding 3	0.695	0.282	2.468	0.0136

Fixed effects of the GLMM model after using cubic spline with linear, quadratic and cubic. See also “Fig 3”.

## Discussion

The deleterious effect of inbreeding is of major concern in conservation biology. Threatened species are often kept in captivity and they are frequently managed through captive breeding programmes such as the European Endangered Species Programme (EEP), which very likely represents the most intensive type of population management for endangered species. In the breeding programme of Cuvier’s gazelle in ‘La Hoya’ the change in pairing strategy carried out in 2006 resulted in i) an increase of mean inbreeding, ii) a reduction in population heterozygosity, and iii) an increase in fitness as measured from juvenile survival. Moreover, for the period of time during the breeding programme, we found two contrasting situations regarding the relationship between inbreeding and fitness. We detected an initial stage of inbreeding depression, in which inbred individuals were less fit than non-inbred ones, and a later stage in which this relationship disappeared and even reversed, so that inbred individuals showed higher fitness than non-inbred ones. This suggests that in the Cuvier’s gazelle of ‘La Hoya’ a process of purging might have occurred. “Fig 3” illustrates this possible process of purging by analyzing the temporal relationship between inbreeding and juvenile mortality. Up to mid-90s, the inbreeding coefficient of those individuals that died before reaching the age of 14 days was higher than that of the survivors, corresponding with an ongoing process involving inbreeding depression. As time progressed, the difference in the inbreeding coefficient between dead individuals and survivors tended to equalize. This result is in agreement with selection acting against deleterious alleles by means of the observed higher mortality of inbred individuals [19]. In the midpoint of the longitudinal study (“Fig 3”, central panel) we found a transitional period during which inbreeding of survivors and non-survivors did not differ, suggesting that genetic load may be low enough to prevent the expression of inbreeding depression [69]. After that period and particularly after the change in pairing management (“Fig 3” right panel) we found inbred individuals having higher fitness in terms of juvenile survival compared to non-inbred ones. These results can be explained largely if a process of purging has taken place [9, 14–18, 21].

However, since inbreeding depression occurs mostly in stressful conditions [10], improvements in husbandry could have led to higher average survival in the captive population as well [70]. Our records at La Hoya Field Station allow us to discard the possibility that any major change in husbandry has taken place during the study period, although we cannot fully rule out that some unnoticed influence might have occurred. Nevertheless, we found not only an increase in population fitness but also a reversal in the relationship between inbreeding and fitness before and after the change in pairing strategy that occurred in 2006, which cannot be explained by the husbandry conditions shared by all the individuals.

It could be argued that what happens for this population might be more extreme than what would be expected for others due to the extremely low number of founders of the studied population. But in many captive breeding programmes of endangered species the number of founders is not greater than ours. For example, *Gazella spekei* began its captive breeding programme with 1 male: 3 females [71]; *Tragelaphus imberbis* began with 2 males: 2 females [72]; *Canis rufus* began with 2 males: 1 female [73]… Hence, what we find in Cuvier’s gazelle might not be exceptional and could explain, for example, the high level of genetic variation found by Templeton et al. [74] in the species they studied.

Our results provide new insights into the genetic basis of the inbreeding effects on juvenile mortality in our Cuvier’s gazelle population. At least in an important proportion of the genes related to juvenile mortality, the genotypic value of the homozygotes for non-deleterious alleles should be higher (higher probability to survive) than the genotypic value of the heterozygotes, which may include non-deleterious and deleterious alleles. This result is in agreement with a partial or incomplete dominance relationship between homologous alleles at genes influencing survival [12–13, 75].

Ibáñez and coauthors [65] have recently shown that in this Cuvier’s gazelle population maternal contribution to juvenile survival through genetic and environmental effects seems to be of major importance. That is, there exist differences among mothers (and among the environment they provide to their offspring) which are influencing the probability of survival for their offspring as well, which agrees with our results of mother identity being significant when introduced in the analyses as a random factor explaining 36% of the variability in offspring survival.

Templeton and Read [5] suggested that, according to theory, controlled inbreeding could be used to purge the genetic load in captive breeding programmes. However, Boakes et al. [8] argued against this practice in zoo populations of threatened species in spite of finding evidence of purging in some of the populations they studied (see [9] for a review). Larsen and coauthors [76] studied captive populations of guppy *(Poecilia reticulata)* and proposed that contrary to full-sib mating or selfing, a slow accumulation of inbreeding over several generations should promote purging. Pekkala et al. [21] made inbreeding experiments with *Drosophila* and concluded that strong inbreeding can produce population decline and that only slow rates of inbreeding are able to effectively purge the frequency of highly deleterious alleles without compromising population viability. The mean number of generations in the pedigree for our studied gazelles is 4 (range: 0–7) (see [30] for details). Considering that inbreeding accumulation is not linear, and its effects may depend on the pedigree depth (e.g., number of generations) [50, 77], our findings could be interpreted as a progressive process of inbreeding across several generations allowing the purging of deleterious alleles in agreement with Larsen et al. [76] and Pekkala et al. [21] conclusions, also giving support to Templeton and Read’s [5] suggestion for gazelles' captive breeding programmes [6].

Clear evidence for purging is rare even for laboratory species with short generation times and it is almost lacking for large mammals [17]. Understanding how small or medium size populations respond to inbreeding in captive programmes is of great importance. Our studied population experienced heterozygosity loss and inbreeding increase, but juvenile survival increased across generations and it was even higher for homozygous individuals, thus supporting the suggestion that purging has occurred and led to a rebound of fitness and a reduction of genetic load, in agreement with the partial dominance hypothesis as the mechanism underlying inbreeding depression [78].

Our results also indicate that purging may be an efficient mechanism to decrease genetic load, it being therefore a potential tool in the management of this captive population. However, many other aspects should be considered before implementing inbreeding and purging as elements in captive breeding management decisions. On one hand, inbreeding may increase the loss not only of deleterious alleles but also of some proportion of beneficial genetic variants due to drift [9, 15]. This may entail risks for the long term maintenance of small captive populations due to depletion of genetic variability [21, 79]. On the other hand, purging alleles under the conditions of captivity may produce departures from the genetic composition of original wild populations, as trait values under captivity conditions may differ from those under natural selection in their natural ecosystems [80–82]. These considerations are especially relevant when captive stocks should provide specimens to restocking natural areas.

EEPs of most endangered species are mostly spawned and coordinated by zoos. No zoo has unlimited space, money or possibilities to maintain all new born individuals, and culling and euthanizing surplus animals (those that are no longer needed for the goals of the EEP) are already included in the World Association of Zoos and Aquaria (WAZA) ethical standards as available management tools. The reported effect of mother inbreeding on offspring sex-ratio bias to daughters [38–39] opens an interesting opportunity to use some controlled inbreeding to reduce the production of surplus males. In spite of the negative consequences on any conscious increase of inbreeding for the long term viability of a captive population managed under an EEP, if rigorously conducted upon previous and scientifically based knowledge of that population (genetic, demographic, behavioural,…), it might represent a better management option than culling.

## Supporting Information

S1 File Studbook of Cuvier’s gazelle, taken from [41]. (PDF)

S2 File Genotypes for the seven microsatellite markers used to estimate genetic diversity in Cuvier’s gazelle. PERIOD indicates whether individuals (*ID*) were born before (*P1*) or after (*P2*) the pairing strategy changes in 2006.
(PDF)

## References

[1] Darwin C (1876) The Effects of Cross and Self-Fertilization in the Vegetable Kingdom. John Murray, London.

[2] Ralls K, Ballou J (1983) Extinction: Lessons from zoos In: Schonewald-Cox CM, Chambers SM, MacBryde B, Thomas L (eds.), Genetics and Conservation: A Reference for Managing Wild Animal and Plant Populations. Benjamin/Cummings, Merlo Park, CA, pp. 164–184.

[3] Crnokrak P, Roff DA (1999) Inbreeding depression in the wild. Heredity 83:260–270. 10504423 10.1038/sj.hdy.6885530

[4] Frankham R, Ballou JD, Briscoe DA (2002) Introduction to Conservation Genetics. Cambridge University Press, Cambridge, UK.

[5] Templeton AR, Read B (1984) Factors eliminating inbreeding depression in a captive herd of Spekes Gazelle (*Gazella spekei*). Zoo Biol 3:177–199.

[6] Hedrick PW (1994) Purging inbreeding depression and the probability of extinction: full-sib mating. Heredity 73:363–372. 7989216 10.1038/hdy.1994.183

[7] Zschoekke S, Baur B (2002) Inbreeding, outbreeding, infant growth, and size dimorphism in captive Indian Rhinoceros (*Rhinoceros unicornis*). Can J Zool 80:2014–2023.

[8] Boakes EH, Wang J, Amos W (2007) An investigation of inbreeding depression and purging in captive pedigreed populations. Heredity 98:172–182. 17180167 10.1038/sj.hdy.6800923

[9] Leberg PL, Firmin BD (2008) Role of inbreeding depression and purging in captive breeding and restoration programmes. Mol Ecol 17:334–343. 10.1111/j.1365-294X.2007.03433.x 18173505

[10] Fox CW, Reed DH (2011) Inbreeding depression increases with environmental stress: an experimental study and meta-analysis. Evolution 65:246–258. 10.1111/j.1558-5646.2010.01108.x 20731715

[11] Charlesworth D, Charlesworth B (1987) Inbreeding depression and its evolutionary consequences. Annu Rev Ecol Syst 18:237–268.

[12] Roff DA (2002) Inbreeding depression: tests of the overdominance and partial dominance hypotheses. Evolution 56:768–75. 12038534 10.1111/j.0014-3820.2002.tb01387.x

[13] Charlesworth D, Willis JH (2009) The genetics of inbreeding depression. Nat Rev Genet 10:783–796. 10.1038/nrg2664 19834483

[14] Barrett SCH, Charlesworth D (1991) Effects of a change in the level of inbreeding on the genetic load. Nature 352:522–524. 1865906 10.1038/352522a0

[15] Hedrick PW (2002) Lethals in finite populations. Evolution 56:654–657. 11989693 10.1111/j.0014-3820.2002.tb01374.x

[16] Glémin S (2003) How are deleterious mutations purged? Drift versus nonrandom mating. Evolution 57:2678–2687. 14761049 10.1111/j.0014-3820.2003.tb01512.x

[17] Crnokrak P, Barret SC (2002) Perspective: purging the genetic load: a review of the experimental evidence. Evolution 56:2347–58. 12583575 10.1111/j.0014-3820.2002.tb00160.x

[18] Xue Y, Prado-Martinez J, Sudmant PH, Narasimhan V, Ayub Q, Szpak M, et al (2015) Mountain gorilla genomes reveal the impact of long-term population decline and inbreeding. Science 348:342–345. 10.1126/science.aaa3952 PMC4668944 25859046

[19] Ohta T, Cockerham CC (1974) Detrimental genes with partial selfing and effects on a neutral locus. Genet Res 23:191–200. 4421247 10.1017/s0016672300014816

[20] Jamieson IG, Wallis GP, Briskie JV (2006) Inbreeding and endangered species management: is New Zealand out of step with the rest of the world? Conserv Biol 20:38–47. 16909657 10.1111/j.1523-1739.2005.00282.x

[21] Pekkala N, Knott KE, Kotiaho JS, Puurtinen M (2012) Inbreeding rate modifies the dynamics of genetic load in small populations. Ecology and Evolution 2:1791–1804. 10.1002/ece3.293 22957182 PMC3433984

[22] Wang J (2000) Effect of population structures and selection strategies on the purging of inbreeding depression due to deleterious mutations. Genetic Res Camb 76:75–86. 10.1017/s0016672399004450 11006636

[23] Robert A (2009) Captive breeding genetic and reintroduction success. Biol Conserv 142:2915–2922.

[24] Fox CW, Scheibly KL, Reed KL (2008) Experimental evolution of the genetic load and its implications for the genetic basis of inbreeding depression. Evolution 62:2236–2249. 10.1111/j.1558-5646.2008.00441.x 18564378

[25] Pelletier F, Réale D, Watters J, Boakes EH, Garant D (2009) Value of captive populations for quantitative genetics research. TREE 24:263–270. 10.1016/j.tree.2008.11.013 19269058

[26] Ogilby WM (1841) Proc Zool Soc 1836–1840:34–35.

[27] Alados CL, Escós JM (1991) Phenotypic and genetic characteristics affecting lifetime reproductive success in female Cuvier´s, dama and dorcas gazelles (*Gazella cuvieri*, *G*. *dama* and *G*. *dorcas*). J Zool Lond 223:307–321.

[28] Roldan ERS, Cassinello J, Abaigar T, Gomendio M (1998) Inbreeding, fluctuating asymmetry, and ejaculate quality in an endangered ungulate. Proc R Soc Lond B 265:243–248. 10.1098/rspb.1998.0288 PMC1688876 9493409

[29] Cassinello J (2005) Inbreeding depression on reproductive performance and survival in captive gazelles of great conservation value. Biol Conserv 122:453–464.

[30] Ibáñez B, Moreno E, Barbosa A (2011) No inbreeding effects on body size in two captive endangered gazelles. Mamm Biol 76:748–754.

[31] Ruiz-López MJ, Espeso G, Evenson DP, Roldán ERS, Gomendio M (2010) Paternal level of DNA damage in spermatozoa and maternal parity influence mortality in an endangered ungulate. Proc R Soc Lond B 277:2541–2546. 10.1098/rspb.2010.0333 PMC2894927 20392732

[32] Ibáñez MB, Moreno E, Barbosa A (2013) Parity, but not inbreeding, affects juvenile mortality in two captive endangered gazelles. Animal Conservation 16:108–117.

[33] Fredrickson R, Hedrick P (2002). Body size in endangered Mexican wolves: Effects of inbreeding and cross-lineage matings. Animal Conservation 5:39–43.

[34] Joron M, Brakefield PM (2003) Captivity masks inbreeding effects on male mating success in butterflies. Nature 424:191–194. 12853956 10.1038/nature01713

[35] Lacy R (1995) Culling Surplus Animals for Population Management, in Norton G. G., Hutchins M., Stevens E. F. and Maple T. R. (eds.), Ethics on the Ark: Ethics, Animal Welfare, and Wildlife Conservation, Washington: Smithsonian Institution Press, pp. 187–194.

[36] Penfold LM, Powell D, Traylor‐Holzer K, Asa CS (2014) “Use it or Lose it”: Characterization, Implications, and Mitigation of Female Infertility in Captive Wildlife. Zoo Biology 33:20–28. 10.1002/zoo.21104 24375838

[37] Moreno E, Espeso G (2008) International studbook. Cuvier´sgazelle (*Gazella cuvieri*). CSIC, Roquetas de Mar, Almería.

[38] Moreno E, Espeso G, Barbosa A (2006) Variation of sex-ratio at birth as a tool for captive breeding programmes: A case study with Cuvier's gazelle. Proceedings of the 23th EAZA Annual Conference. Madrid, Spain.

[39] Moreno E, Ibáñez MB, Barbosa A (2011) Mother traits and offspring sex in two threatened gazelle species in captivity. J Nat Conserv 19:148–153.

[40] Beudels RC, Devillers P, Lafontaine R-M, Devillers-Terschuren J, Beudels M-O (2005) Sahelo-Saharan Antelopes. Status and Perspectives; UNEP/CMS Secretariat: Bonn, Germany, pp. 57–70.

[41] Espeso G, Moreno E (2012) International Cuvier's Gazelle Studbook, *Gazella cuvieri* Accessed 15 June 2015. Available: http://www.eeza.csic.es/eeza/documentos/StdkCuvier01-01-2012.pdf.

[42] ISIS. (2004). International species information system: SPARK Single Population Animal Record Keeping System Software, version 1.54. MN, USA: Eagan.

[43] Wilcken J Lees C (eds) 1998 Managing Zoo populations: compiling and analysing studbook data Australasina Regional Association of Zoological Parks and Aquaria: Sydney, Australia.

[44] Robertson BC, Elliot GP, Eson DK, Clout MN, Gemmel NJ (2006) Sex allocation theory aids species conservation. Biology Letters 2:229–231. 17148369 10.1098/rsbl.2005.0430 PMC1618899

[45] Tella JL (2001) Sex-ratio theory in conservation biology. TREE 16:76–77.

[46] Coltman DW, Pilkington JG, Smith JA, Pemberton JM (1999) Parasite-mediated selection against inbred Soay sheep in a free-living, island population. Evolution 53:1259–1267. 28565537 10.1111/j.1558-5646.1999.tb04538.x

[47] Mitton JB, Schuster WSF, Cothran EG, De Fries JC (1993) Correlation between the individual heterozygosity of parents and their offspring. Heredity 71:59–63. 8360078 10.1038/hdy.1993.107

[48] Reid JM, Arcese P, Keller LF (2006) Intrinsic parent‐offspring correlation in inbreeding level in a song sparrow (*Melospiza melodia*) population open to immigration. Am Nat 168:1–13. 10.1086/504852 16685634

[49] Lacy RC, Ballou JD, Pollak JP (2012) PMx: Software package for demographic and genetic analysis and management of pedigreed populations. Methods in Ecology and Evolution 3:433–437.

[50] González-Recio G, López de Maturana E, Gutiérrez JP (2007) Inbreeding depression on female fertility and calving ease in Spanish dairy cattle. J Dairy Sci 90:5744–5752. 18024768 10.3168/jds.2007-0203

[51] Fair MD, van Wykl JB, Cloete SWP (2012) Pedigree analysis of an ostrich breeding flock. South African Journal of Animal Science 42:114–122.

[52] Buchanan FC, Crawford AM (1993) Ovine microsatellites at the OarFCB11, OarFCB128, OarFCB193, OarFCB266 and OarFCB304 loci. Animal Genetics 24:145 8328699 10.1111/j.1365-2052.1993.tb00269.x

[53] Steffen P, Eggen A, Dietz AB et al (1993) Isolation and mapping of polymorphic microsatellites in cattle. Animal Genetics 24:121–124. 8328693 10.1111/j.1365-2052.1993.tb00252.x

[54] Bishop MD, Kappes SM, Keele JW et al (1994) A genetic linkage map for cattle. Genetics 130:619–639. 10.1093/genetics/136.2.619 PMC1205813 7908653

[55] Moore SS, Byrne K, Berger KT et al (1994) Characterization of 65 bovine microsatellites. Mammalia Genome 5:84–90. 10.1007/BF00292333 8180478

[56] Grosse WM, Finlay O, Kossarek LM, Clark TG, McGraw RA (1995) Five bovine microsatellite markers derived from skeletal muscle cDNA, RME01, RME11, RME23, RME25 and RME33. Animal Genetics 25:126–127. 10.1111/j.1365-2052.1995.tb02652.x 7733500

[57] Ede AJ, Pierson CA, Crawford AM (1995) Ovine microsatellites at the OARCP9, OARCP16, OARCP20, OARCP21, OARCP23 and OARCP26 loci. Animal Genetics 25:129–30. 10.1111/j.1365-2052.1995.tb02655.x 7733503

[58] Mommens G, Coppieters W, Van de Weghe A, Van Zeveren A, Bouquet Y (1994) Dinucleotide repeat polymorphism at the bovine MM12E6 and MM8D3 loci. Animal Genetics 25:368. 10.1111/j.1365-2052.1994.tb00381.x 7818181

[59] Coulson TN, Pemberton JM, Albon SD, Beaumont M, Marshall TC, Slate J, et al (1998) Microsatellites reveal heterosis in red deer. Proceedings of the Royal Society B: Biological Sciences 265:489–495. 9569667 10.1098/rspb.1998.0321 PMC1688908

[60] Raymond M, Rousset F (1995) GENEPOP (version 1.2): population genetics software for exact tests and ecumenicism. J Hered 86:248–249.

[61] Belkhir K, Borsa P, Chikhi L, Raufaste N, Bonhomme F (2004) GENETIX 4.05, logiciel sous Windows TM pour la génétique des populations. Laboratoire Génome, populations, interactions, CNRS UMR 5171, Université de Montpellier II, Montpellier (France).

[62] Wright S (1969) Evolution and the Genetics of Populations, vol. 2: The Theory of Gene Frequencies. University of Chicago Press, Chicago.

[63] Legendre P, Legendre L (1998) Numerical Ecology, second English ed. Elsevier Science BV, Amsterdam.

[64] R Core Team (2015) R: A language and environment for statistical computing R Foundation for Statistical Computing, Vienna, Austria ISBN 3-900051-07-0. Available: http://www.R-project.org/.

[65] Ibáñez B, Cervantes I, Gutiérrez JP, Goyache F, Moreno E (2014) Estimates of direct and indirect effects for early juvenile survival in captive populations maintained for conservation purposes: the case of Cuvier’s gazelle. Ecology and Evolution 2014:4117–4129. 10.1002/ece3.1280 PMC4242564 25505538

[66] Johnstone-Yellin TL, Shipley LA, Myers WL 0Robinson HS (2009) To twin or not to twin? Trade-offs in litter size and fawn survival in mule deer. J Mammal 90: 453–460.

[67] Nakagawa S, Schielzeth H (2013) A general and simple method for obtaining R2 from generalized linear mixed-effects models, Methods in Ecol Evol 14:133–142.

[68] Fox J (2003) Effect Displays in R for Generalised Linear Models. Journal of Statistical Software 8:1–9.

[69] Crown JF (1993) Mutation, mean fitness, and genetic load In: Futuyma D, Antonovics J (eds), Oxford surveys in evolutionary biology. Vol. 9 Oxford University Press, Oxford, pp. 3–42.

[70] Kalinowski ST, Hedrick PW, Miller PS (1999) No inbreeding depression observed in Mexican and red wolf captive breeding programmes. Conserv Biol 13:1371–1377.

[71] Templeton AR, Read B (1983) The elimination of inbreeding depression in a captive herds of Speke’s gazelle, in Schonewald-Cox C.M., Chambers S.M., MacBryde B. and Thomas L. (eds.), Genetics and conservation, Menlo Park, California: Benjamin/Cummings, pp. 241–261.

[72] Steck B (2014) Lesser Kudu European Studbook. Accessed 25 August 2015. Available: http://eaza.portal.isis.org/member_area/TAGs/Antelope/Shared%20Documents/2014%20Lesser%20kudu%20ESB%20Studbook.pdf).

[73] Hedrick PW, Miller PS, Geffen E, Wayne R (1997) Genetic evaluation of the three captive Mexican wolf lineages. Zoo Biology 16:47–69.

[74] Templeton AR, Davis SK, Read B (1987) Genetic variability in a captive herd of Spekes gazelles (*Gazella spekei*). Zoo Biol 6:305–313.5.

[75] Falconer DS, Mackay TFC (1996) Introduction to Quantitative Genetics. Longman, London.

[76] Larsen L K, Pelabon C, Bolstad GH, Viken A, Fleming IA, Rosenqvist G (2011) Temporal change in inbreeding depression in life-history traits in captive populations of guppy (*Poecilia reticulata*): evidence for purging? J Evol Biol 24:823–834. 10.1111/j.1420-9101.2010.02224.x 21276111

[77] Gutiérrez JP, Cervantes I, Molina A, Valera M, Goyache F (2008) Individual increase in inbreeding allows estimating realized effective sizes from pedigrees. Genet Sel Evol 40:359–378. 10.1051/gse:2008008 18558071 PMC2674907

[78] Charlesworth DMT, Morgan MT, Charlesworth B (1990) Inbreeding depression, genetic load, and the evolution of outcrossing rates in a multi-locus system with no linkage. Evolution 44:1469–1489. 28564321 10.1111/j.1558-5646.1990.tb03839.x

[79] Reed DH, Bryant EH (2000) Experimental tests of minimum viable population size. Anim Conserv 3:7–14.

[80] Bijlsma R, Bundgaard J, Van Putten WF (1999) Environmental dependence of inbreeding depression and purging in *Drosophila melanogaster* . J Evol Biol 12:1125–1137.

[81] Van Oosterhout C, Trigg RE, Carvalho GR, Magurran AE, Hauser L, Shaw PW (2003) Inbreeding depression and genetic load of sexually selected traits: how the guppy lost its spots. J Evol Biol 16:273–281. 14635866 10.1046/j.1420-9101.2003.00511.x

[82] Frankham R (2008) Genetic adaptation to captivity in species conservation programs. Mol Ecol 17:325–333. 10.1111/j.1365-294X.2007.03399.x 18173504

