# Correction: Better Fitness in Captive Cuvier's Gazelle despite Inbreeding Increase: Evidence of Purging?

**DOI:** 10.1371/journal.pone.0152542

**Published:** 2016-03-24

**Authors:** Eulalia Moreno, Javier Pérez-González, Juan Carranza, Jordi Moya-Laraño

Fig 3 appears incorrectly in the published article. Please see the correct Fig 3 and its legend here.

**Fig 3:**
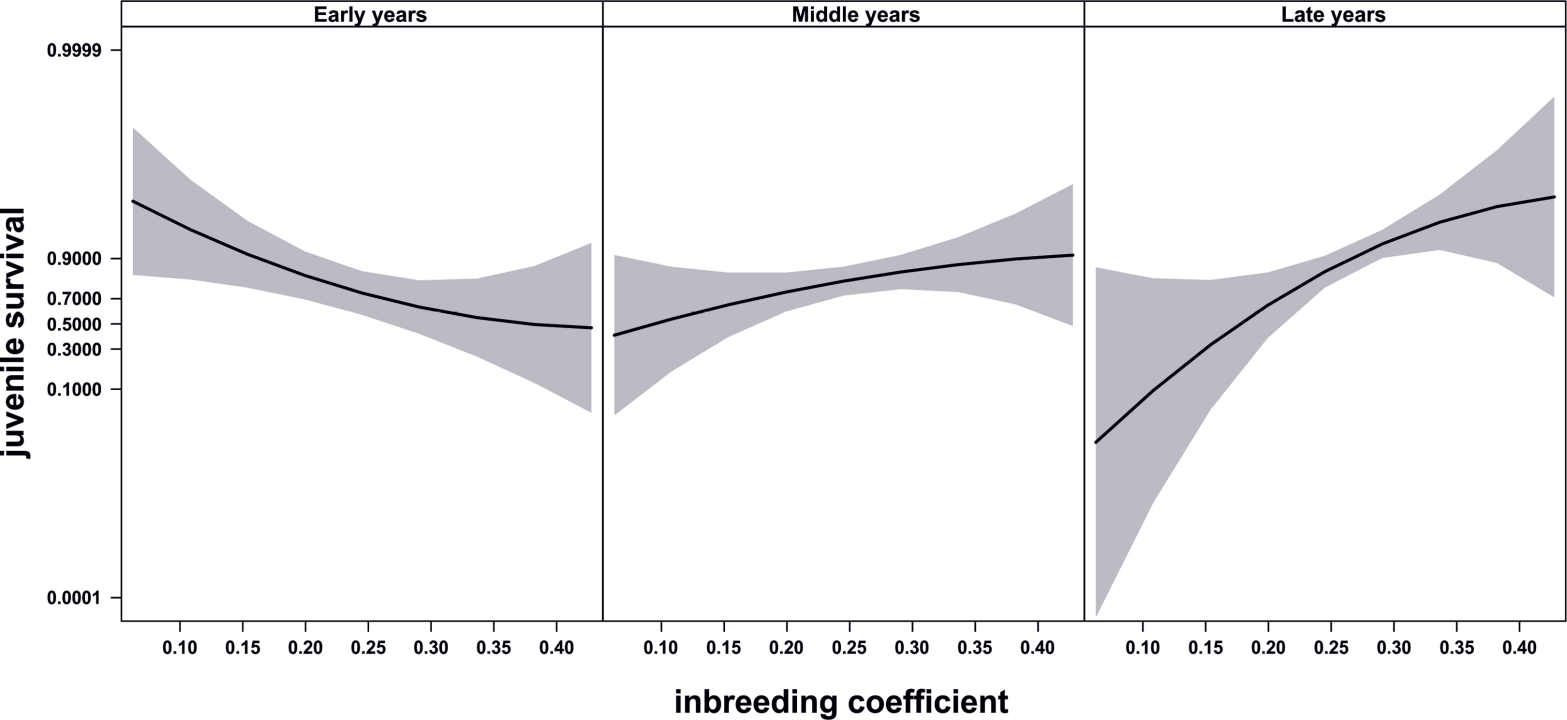
Evolution of juvenile survival through time. Relationship between inbreeding and juvenile survival through time. Juvenile survival is a dichotomous variable in which 0 indicates dead individuals and 1 indicates surviving individuals. Figure shows predicted values and 95% confidence bands. Time progresses from left to right, so the left graph shows the relationship between inbreeding and juvenile survival centred in year 1981, while the right graph shows this relationship centred in year 2006 when a major change in pairing management occurred. Graphs were produced with library “effects” [68], which uses the estimates of the effects from GLMMs to predict the values across the entire expand of the explanatory variables. Confidence bands are calculated from standard errors estimated at each of three levels of inbreeding (0.0625, 0.2451 and 0.4277) within each of the time periods.
