# Supplementary material for: Better Fitness in Captive Cuvier’s Gazelle despite Inbreeding Increase: Evidence of Purging?
Source: PLoS One. 2015 Dec 17;10(12):e0145111. doi: 10.1371/journal.pone.0145111 (PMC4682998; doi:10.1371/journal.pone.0145111)
Supplement: S1 File — (PDF) [file pone.0145111.s001.pdf]

Supporting information

S1 File: Studbook of Cuvier's gazelle, taken from Espeso and Moreno (2012) and downloadable at http://www.eeza.csic.es/documentos/StdkCuvier01-01-2012.pdf.

International
Cuvier's Gazelle Studbook

Gazella cuvieri

Compiled by:
Gerardo Espeso & Eulalia Moreno

gerardo@eeza.csic.es emoremo@eeza.csic.es

Finca Experimantal La Hoya.
Estación Experimental de Zonas Áridas
Consejo Superior de Investigaciones Científicas
Almeria

Data current as of 01 January 2012

You created this PDF from an application that is not licensed to print to novaPDF printer (http://www.novapdf.com)

Stud#	Sex	Birth Date	Sire	Dam	Location	Date	LocalID	Event	Name
1	M	~ 1973	WILD	WILD	W.AFRICA	7 May 1975	NONE	Capture	MONSENOR
					ALMERIA	7 May 1975	EE1	Transfer	
						26 Apr 1978		Death	
		[Death by: Unknown means]							
2	F	~ 1973	WILD	WILD	W.AFRICA	7 May 1975	NONE	Capture	CHRISTIANE
					ALMERIA	7 May 1975	EE2	Transfer	
						7 Oct 1978		Death	
		[Death by: Unknown means]							
3	F	~ 1973	WILD	WILD	W.AFRICA	7 May 1975	NONE	Capture	CUERNO ROTO
					ALMERIA	7 May 1975	EE3	Transfer	
						12 Aug 1980		Death	
		[Death by: Unknown means]							
4	F	20 Oct 1975	1	2	ALMERIA	20 Oct 1975	EE4	Birth	AIDA
						31 May 1981		Death	
		[Death by: Unknown means]							
5	F	13 Nov 1975	1	3	ALMERIA	13 Nov 1975	EE5	Birth	LUCERO
						18 Dec 1982		Death	
		[Death by: Unknown means]							
6	M	~ 1973	WILD	WILD	W.AFRICA	14 Nov 1975	NONE	Capture	VICTOR
					ALMERIA	14 Nov 1975	EE6	Transfer	
						27 Jul 1979		Death	
		[Death by: Unknown means]							
7	F	3 Jul 1976	1	2	ALMERIA	3 Jul 1976	EE7	Birth	QUINIELA
						1 Jun 1989		Death	
		[Death by: Unknown means]							
8	M	3 Jul 1976	1	2	ALMERIA	3 Jul 1976	EE8	Birth	ARROZ
						29 Jul 1978		Death	
		[Death by: Unknown means]							
9	M	23 Aug 1976	1	3	ALMERIA	23 Aug 1976	EE9	Birth	FERIANTE
						27 Feb 1979		Death	
		[Death by: Unknown means]							

You created this PDF from an application that is not licensed to print to novaPDF printer (http://www.novapdf.com)

10 F 7 Feb 1977 1 4 ALMERIA 7 Feb 1977 EE10
Birth MARTA
15 May 1984
Death
[Death by: Unknown means]

11 F 7 Feb 1977 1 4 ALMERIA 7 Feb 1977 EE11
Birth MARIA
8 Feb 1977
Death
[Death by: Unknown means]

12 M 14 Feb 1977 1 5 ALMERIA 14 Feb 1977 EE12
Birth LUCERO II
16 Jul 1978
Death
[Death by: Unknown means]

13 M 14 Feb 1977 1 5 ALMERIA 14 Feb 1977 EE13
Birth PATAS BLANCA
11 Apr 1978
Death
[Death by: Unknown means]

14 M 18 Feb 1977 1 2 ALMERIA 18 Feb 1977 EE14
Birth ABELARDO
MUNICH 13 Oct 1980 1
Transfer
SANDIEGOZ 15 May 1982 282276
Transfer
9 Feb 1990
Death
[Death by: Unknown means]

15 F 18 Feb 1977 1 2 ALMERIA 18 Feb 1977 EE15
Birth ELOISA
17 Jan 1979
Death
[Death by: Unknown means]

16 M 11 Mar 1977 1 3 ALMERIA 11 Mar 1977 EE16
Birth CASTOR
16 Sep 1982
Death
[Death by: Unknown means]

17 M 11 Mar 1977 1 3 ALMERIA 11 Mar 1977 EE17
Birth POLUX
9 Sep 1987
Death
[Death by: Unknown means]

18 M 13 Jun 1977 1 7 ALMERIA 13 Jun 1977 EE18
Birth SAN ANTONIO
16 Jun 1977
Death
[Death by: Unknown means]

19 M 13 Aug 1977 1 4 ALMERIA 13 Aug 1977 EE19
Birth SN
14 Aug 1977
Death
[Death by: Unknown means]

20 F 21 Sep 1977 1 3 ALMERIA 21 Sep 1977 EE20
Birth SUSANA
23 May 1988
Death

You created this PDF from an application that is not licensed to print to novaPDF printer (http://www.novapdf.com)

```
                        [Death by: Unknown means]

        21   F    21 Sep 1977       1       3  ALMERIA    21 Sep 1977   EE21
Birth     CASTA
                                                          22 Sep 1977
Death
                        [Death by: Unknown means]

        22   F    24 Sep 1977       1       5  ALMERIA    24 Sep 1977   EE22
Birth     RAQUEL
                                               MUNICH     13 Oct 1980      6
Transfer
                                               L RUHE      5 Jul 1988    UNK
Transfer
                                               SANDIEGOZ  16 Nov 1988 588433
Transfer
                                                          14 May 1993
Death
                        [Death by: Unknown means]

        23   F     2 Oct 1977       1       2  ALMERIA     2 Oct 1977   EE23
Birth     PILI
                                                          28 May 1979
Death
                        [Death by: Unknown means]

        24   F     2 Oct 1977       1       2  ALMERIA     2 Oct 1977   EE24
Birth     MILI
                                                          18 Feb 1983
Death
                        [Death by: Unknown means]

        25   M     2 Jan 1978       8       7  ALMERIA     2 Jan 1978   EE25
Birth     SN
                                                           3 Jan 1978
Death
                        [Death by: Unknown means]

        26   F     2 Jan 1978       8       7  ALMERIA     2 Jan 1978   EE26
Birth     REYES
                                                          25 Aug 1980
Death
                        [Death by: Unknown means]

        27   M    23 Feb 1978       8       4  ALMERIA    23 Feb 1978   EE27
Birth     RUBIO
                                                           4 Apr 1978
Death
                        [Death by: Unknown means]

        28   F    23 Feb 1978       8       4  ALMERIA    23 Feb 1978   EE28
Birth     MELERA
                                                          26 Feb 1983
Death
                        [Death by: Unknown means]

        29   F    10 Mar 1978       8      15  ALMERIA    10 Mar 1978   EE29
Birth     SN
                                                          10 Mar 1978
Death
                        [Death by: Unknown means]

        30   M    18 Mar 1978       8       5  ALMERIA    18 Mar 1978   EE30
Birth     MANCHADO
                                                           2 Sep 1978
Death
                        [Death by: Unknown means]

        31   F    18 Mar 1978       8       5  ALMERIA    18 Mar 1978   EE31
```


You created this PDF from an application that is not licensed to print to novaPDF printer (http://www.novapdf.com)

Birth NORMAL
22 Jan 1983
Death
[Death by: Unknown means]

32 F 20 Mar 1978 8 3 ALMERIA 20 Mar 1978 EE32
Birth MELI
MUNICH 13 Oct 1980 4
Transfer
SANDIEGOZ 11 May 1982 282278
Transfer
7 Dec 1994
Death
[Death by: Euthanasia (medical) + Unknown + No necropsy planned]

33 F 20 Mar 1978 8 3 ALMERIA 20 Mar 1978 ______
Birth ELISA
MUNICH 13 Oct 1980 5
Transfer
SANDIEGOZ 11 May 1982 282285
Transfer
2 Dec 1982
Death
[Death by: Unknown means]

34 F 22 Mar 1978 8 10 ALMERIA 22 Mar 1978 EE34
Birth SOLA
5 Mar 1979
Death
[Death by: Unknown means]

35 F 28 Mar 1978 8 2 ALMERIA 28 Mar 1978 EE35
Birth JEANETTE
8 Nov 1981
Death
[Death by: Unknown means]

36 M 10 Jul 1978 8 7 ALMERIA 10 Jul 1978 EE36
Birth PACO
ALMER.CTY 1 Oct 1983
Death
[Death by: Unknown means]

37 F 10 Jul 1978 8 7 ALMERIA 10 Jul 1978 EE37
Birth MAR
13 Jul 1978
Death
[Death by: Unknown means]

38 F 24 Sep 1978 8 4 ALMERIA 24 Sep 1978 EE38
Birth CUMBRE
28 Oct 1980
Death
[Death by: Unknown means]

39 F 24 Sep 1978 8 4 ALMERIA 24 Sep 1978 EE39
Birth CIMA
25 Jun 1979
Death
[Death by: Unknown means]

40 M 6 Oct 1978 8 5 ALMERIA 6 Oct 1978 EE40
Birth AGUJETAS
24 Mar 1982
Death
[Death by: Unknown means]

41 M 6 Oct 1978 8 5 ALMERIA 6 Oct 1978 EE41

You created this PDF from an application that is not licensed to print to novaPDF printer (http://www.novapdf.com)

Birth ARA¥AZO

26 Mar 1979

Death

[Death by: Unknown means]

42 M 23 Oct 1978 8 3 ALMERIA 23 Oct 1978 EE42

Birth RETRASADO

MUNICH 13 Oct 1980 3

Transfer

SANDIEGOZ 11 May 1982 282277

Transfer

3 Dec 1991

Death

[Death by: Unknown means]

43 M 14 Feb 1979 8 22 ALMERIA 14 Feb 1979 EE43

Birth SN

14 Feb 1979

Death

[Death by: Unknown means]

44 F 24 Feb 1979 8 20 ALMERIA 24 Feb 1979 EE44

Birth SN

25 Feb 1979

Death

[Death by: Unknown means]

45 M 24 Feb 1979 8 20 ALMERIA 24 Feb 1979 EE45

Birth LEVI

31 Aug 1980

Death

[Death by: Unknown means]

46 F 1 Mar 1979 14 7 ALMERIA 1 Mar 1979 EE46

Birth BLANCA

21 Jul 1979

Death

[Death by: Unknown means]

47 F 1 Mar 1979 14 7 ALMERIA 1 Mar 1979 EE47

Birth AURORA

21 Mar 1981

Death

[Death by: Unknown means]

48 M 5 Mar 1979 14 23 ALMERIA 5 Mar 1979 EE48

Birth SN

6 Mar 1979

Death

[Death by: Unknown means]

49 M 10 Mar 1979 17 10 ALMERIA 10 Mar 1979 EE49

Birth MERCURIO

17 Sep 1979

Death

[Death by: Unknown means]

50 F 10 Mar 1979 17 10 ALMERIA 10 Mar 1979 EE50

Birth VENUS

10 May 1980

Death

[Death by: Unknown means]

51 M 10 Mar 1979 14 24 ALMERIA 10 Mar 1979 EE51

Birth JUPITER

14 Mar 1980

Death

[Death by: Unknown means]

You created this PDF from an application that is not licensed to print to novaPDF printer (http://www.novapdf.com)

52 M 10 Mar 1979 14 24 ALMERIA 10 Mar 1979 EE52
Birth SATURNO
18 Oct 1980
Death
[Death by: Unknown means]

53 F 13 Mar 1979 17 28 ALMERIA 13 Mar 1979 EE53
Birth DEBORA
27 Jun 1986
Death
[Death by: Unknown means]

54 M 14 Mar 1979 17 31 ALMERIA 14 Mar 1979 EE54
Birth SN
16 Mar 1979
Death
[Death by: Unknown means]

55 M 15 Mar 1979 14 26 ALMERIA 15 Mar 1979 EE55
Birth SN
16 Mar 1979
Death
[Death by: Unknown means]

56 M 20 Mar 1979 17 32 ALMERIA 20 Mar 1979 EE56
Birth SN
22 Mar 1979
Death
[Death by: Unknown means]

57 F 23 Mar 1979 17 33 ALMERIA 23 Mar 1979 EE57
Birth SN
23 Mar 1979
Death
[Death by: Unknown means]

58 M 29 Mar 1979 14 4 ALMERIA 29 Mar 1979 EE58
Birth SN
29 Mar 1979
Death
[Death by: Unknown means]

59 M 26 Apr 1979 14 3 ALMERIA 26 Apr 1979 EE59
Birth BLANCO
14 Sep 1987
Death
[Death by: Unknown means]

60 M 26 Apr 1979 14 3 ALMERIA 26 Apr 1979 EE60
Birth NEGRO
26 Dec 1983
Death
[Death by: Unknown means]

61 M 27 Apr 1979 14 5 ALMERIA 27 Apr 1979 EE61
Birth SOLO
7 Jun 1979
Death
[Death by: Unknown means]

62 M 20 Sep 1979 14 7 ALMERIA 20 Sep 1979 EE62
Birth HANSEL
13 Feb 1981
Death
[Death by: Unknown means]

63 F 20 Sep 1979 14 7 ALMERIA 20 Sep 1979 EE63
Birth GRETEL
29 Apr 1981

You created this PDF from an application that is not licensed to print to novaPDF printer (http://www.novapdf.com)

Death
[Death by: Unknown means]

64 M 29 Sep 1979 14 26 ALMERIA 29 Sep 1979 EE64
Birth ABAD
4 Jan 1980
Death
[Death by: Unknown means]

65 M 11 Oct 1979 14 24 ALMERIA 11 Oct 1979 EE65
Birth SN
12 Oct 1979
Death
[Death by: Unknown means]

66 F 13 Oct 1979 14 4 ALMERIA 13 Oct 1979 EE66
Birth ALHAMA
MUNICH 13 Oct 1980 8
Transfer
31 May 1983
Death
[Death by: Unknown means]

67 M 12 Nov 1979 14 5 ALMERIA 12 Nov 1979 EE67
Birth RICO
MUNICH 13 Oct 1980 2
Transfer
13 Jul 1985
Death
[Death by: Unknown means]

68 F 12 Nov 1979 14 5 ALMERIA 12 Nov 1979 EE68
Birth LUISA
MUNICH 13 Oct 1980 7
Transfer
SANDIEGOZ 15 Sep 1987 587314
Transfer
23 May 1988
Death
[Death by: Unknown means]

69 F 22 Nov 1979 14 3 ALMERIA 22 Nov 1979 EE69
Birth BARBARA
30 Sep 1986
Death
[Death by: Unknown means]

70 F 24 Feb 1980 16 28 ALMERIA 24 Feb 1980 EE70
Birth PACA
4 Sep 1980
Death
[Death by: Unknown means]

71 F 24 Feb 1980 16 28 ALMERIA 24 Feb 1980 EE71
Birth FRANCISCA
3 Nov 1992
Death
[Death by: Unknown means]

72 F 27 Mar 1980 17 47 ALMERIA 27 Mar 1980 EE72
Birth MARTIRIO
19 Nov 1984
Death
[Death by: Unknown means]

73 F 3 Apr 1980 16 53 ALMERIA 3 Apr 1980 EE73
Birth KARIN
14 Apr 1983
Death

You created this PDF from an application that is not licensed to print to novaPDF printer (http://www.novapdf.com)

[Death by: Unknown means]

74 F 23 Apr 1980 16 50 ALMERIA 23 Apr 1980 EE74
Birth EMCARNA
27 Aug 1980
Death
[Death by: Unknown means]

75 M 29 May 1980 17 3 ALMERIA 29 May 1980 EE75
Birth GUIRADO
31 Jan 1982
Death
[Death by: Unknown means]

76 M 16 Oct 1980 16 28 ALMERIA 16 Oct 1980 EE76
Birth UNICO
5 May 1981
Death
[Death by: Unknown means]

77 M 22 Feb 1981 16 53 ALMERIA 22 Feb 1981 EE77
Birth FEO
14 Jul 1981
Death
[Death by: Unknown means]

78 M 22 Feb 1981 16 53 ALMERIA 22 Feb 1981 EE78
Birth GUAPO
6 Jan 1982
Death
[Death by: Unknown means]

79 M 18 Mar 1981 17 47 ALMERIA 18 Mar 1981 EE79
Birth ALBACETE
2 Apr 1981
Death
[Death by: Unknown means]

80 F 17 Apr 1981 16 28 ALMERIA 17 Apr 1981 EE80
Birth ENKA
1 Oct 1990
Death
[Death by: Unknown means]

81 F 5 May 1981 17 72 ALMERIA 5 May 1981 EE81
Birth
23 Jul 1981
Death
[Death by: Unknown means]

82 M 29 May 1981 17 4 ALMERIA 29 May 1981 EE82
Birth CEMENTO
17 Jun 1981
Death
[Death by: Unknown means]

83 M 29 May 1981 17 4 ALMERIA 29 May 1981 EE83
Birth HORMIGON
22 Jun 1981
Death
[Death by: Unknown means]

84 F 11 Sep 1981 16 53 ALMERIA 11 Sep 1981 EE84
Birth BALSA
13 Jun 1982
Death
[Death by: Unknown means]

85 F 7 Nov 1981 16 28 ALMERIA 7 Nov 1981 EE85

You created this PDF from an application that is not licensed to print to novaPDF printer (http://www.novapdf.com)

Birth CORDOBA
15 Jul 1991
Death
[Death by: Unknown means]

86 F 7 Nov 1981 16 28 ALMERIA 7 Nov 1981 EE86
Birth SEVILLA
2 Jun 1997
Death
[Death by: Unknown means]

87 F 9 Dec 1981 17 5 ALMERIA 9 Dec 1981 EE87
Birth GRANADA
13 Mar 1983
Death
[Death by: Unknown means]

88 M 24 Feb 1982 16 71 ALMERIA 24 Feb 1982 EE88
Birth JAEN
22 Jun 1982
Death
[Death by: Unknown means]

89 F 24 Feb 1982 16 71 ALMERIA 24 Feb 1982 EE89
Birth MALAGA
19 Apr 1987
Death
[Death by: Unknown means]

90 M 6 Mar 1982 17 72 ALMERIA 6 Mar 1982 EE90
Birth PEDRAJAS
22 Nov 1983
Death
[Death by: Unknown means]

91 M 6 Mar 1982 17 72 ALMERIA 6 Mar 1982 EE91
Birth PRIEGO
15 Oct 1986
Death
[Death by: Unknown means]

92 M 7 Mar 1982 16 73 ALMERIA 7 Mar 1982 EE92
Birth LUGO
11 Dec 1983
Death
[Death by: Unknown means]

93 M 7 Mar 1982 16 73 ALMERIA 7 Mar 1982 EE93
Birth ORENSE
25 Aug 1982
Death
[Death by: Unknown means]

94 F 11 Mar 1982 59 69 ALMERIA 11 Mar 1982 EE94
Birth PONTEVEDRA
17 Jan 1995
Death
[Death by: Unknown means]

95 M 19 Mar 1982 16 53 ALMERIA 19 Mar 1982 EE95
Birth JAVI
25 Nov 1989
Death
[Death by: Unknown means]

96 F 19 Mar 1982 16 53 ALMERIA 19 Mar 1982 EE96
Birth BERTA
5 Dec 1984
Death

You created this PDF from an application that is not licensed to print to novaPDF printer (http://www.novapdf.com)

[Death by: Unknown means]

97 M 7 Jun 1982 16 28 ALMERIA 7 Jun 1982 EE97
Birth CANTANTE
16 Jun 1988
Death
[Death by: Unknown means]

98 M 9 Jun 1982 17 5 ALMERIA 9 Jun 1982 EE98
Birth MOTOR
28 Mar 1987
Death
[Death by: Unknown means]

99 M 9 Jun 1982 17 5 ALMERIA 9 Jun 1982 EE99
Birth POZO
16 Oct 1985
Death
[Death by: Unknown means]

100 M 13 Oct 1982 16 53 ALMERIA 13 Oct 1982 EE100
Birth MINUSVALIDO
14 Oct 1982
Death
[Death by: Unknown means]

101 F 26 Nov 1982 59 69 ALMERIA 26 Nov 1982 EE101
Birth CAPICUA
ALMER.CTY 26 Feb 1987
Death
[Death by: Unknown means]

102 F 14 Feb 1983 59 20 ALMERIA 14 Feb 1983 EE102
Birth LUISA II
2 Jan 1984
Death
[Death by: Unknown means]

103 F 14 Feb 1983 59 20 ALMERIA 14 Feb 1983 EE103
Birth ANTONIA
30 Jul 1990
Death
[Death by: Unknown means]

104 F 23 Feb 1983 16 28 ALMERIA 23 Feb 1983 EE104
Birth SN
28 Feb 1983
Death
[Death by: Unknown means]

105 F 23 Feb 1983 16 28 ALMERIA 23 Feb 1983 EE105
Birth SN
23 Feb 1983
Death
[Death by: Unknown means]

106 M 27 Feb 1983 16 73 ALMERIA 27 Feb 1983 EE106
Birth MATEO I
10 Mar 1983
Death
[Death by: Unknown means]

107 M 27 Feb 1983 16 73 ALMERIA 27 Feb 1983 EE107
Birth SN
28 Feb 1983
Death
[Death by: Unknown means]

108 M 25 Mar 1983 16 71 ALMERIA 25 Mar 1983 EE108

You created this PDF from an application that is not licensed to print to novaPDF printer (http://www.novapdf.com)

Birth JAVIER
27 Jul 1984
Death
[Death by: Unknown means]

109 M 25 Mar 1983 16 71 ALMERIA 25 Mar 1983 EE109
Birth GUSTAVO
12 Sep 1986
Death
[Death by: Unknown means]

110 F 1 Apr 1983 59 80 ALMERIA 1 Apr 1983 EE110
Birth SANTA
30 Jul 1995
Death
[Death by: Unknown means]

111 M 5 Apr 1983 59 85 ALMERIA 5 Apr 1983 EE111
Birth SN
8 Apr 1983
Death
[Death by: Unknown means]

112 M 21 Apr 1983 59 86 ALMERIA 21 Apr 1983 EE112
Birth SN
24 Apr 1983
Death
[Death by: Unknown means]

113 M 26 Apr 1983 60 53 ALMERIA 26 Apr 1983 EE113
Birth MATEO II
5 Jun 1985
Death
[Death by: Unknown means]

114 M 26 Apr 1983 60 53 ALMERIA 26 Apr 1983 EE114
Birth MARCOS
27 Dec 1993
Death
[Death by: Unknown means]

115 M 27 May 1983 17 69 ALMERIA 27 May 1983 EE115
Birth LUCAS
16 Jul 1985
Death
[Death by: Unknown means]

116 M 27 May 1983 17 69 ALMERIA 27 May 1983 EE116
Birth JUAN
15 Feb 1989
Death
[Death by: Unknown means]

117 F 18 Nov 1983 60 53 ALMERIA 18 Nov 1983 EE117
Birth JARA
1 Apr 1984
Death
[Death by: Unknown means]

118 F 18 Nov 1983 60 53 ALMERIA 18 Nov 1983 EE118
Birth ADELFA
29 Jan 1996
Death
[Death by: Unknown means]

119 F 23 Feb 1984 91 71 ALMERIA 23 Feb 1984 EE119
Birth CARPA
20 Mar 1986
Death

You created this PDF from an application that is not licensed to print to novaPDF printer (http://www.novapdf.com)

[Death by: Unknown means]

120 F 29 Feb 1984 91 86 ALMERIA 29 Feb 1984 EE120
Birth SN
6 Mar 1984
Death
[Death by: Unknown means]

121 M 2 Mar 1984 92 69 ALMERIA 2 Mar 1984 EE121
Birth CANDIDO
5 Oct 1985
Death
[Death by: Unknown means]

122 F 2 Mar 1984 92 94 ALMERIA 2 Mar 1984 EE122
Birth CENIZA
25 Feb 1994
Death
[Death by: Unknown means]

123 M 7 Mar 1984 92 10 ALMERIA 7 Mar 1984 EE123
Birth MELITON
8 Mar 1984
Death
[Death by: Unknown means]

124 M 8 Mar 1984 97 20 ALMERIA 8 Mar 1984 EE124
Birth EULOGIO
11 Aug 1985
Death
[Death by: Unknown means]

125 M 8 Mar 1984 97 20 ALMERIA 8 Mar 1984 EE125
Birth ROSENDO
8 Jul 1999
Death
[Death by: Unknown means]

126 M 19 Mar 1984 91 80 ALMERIA 19 Mar 1984 EE126
Birth OLEGARIO
8 Aug 1985
Death
[Death by: Unknown means]

127 F 10 Apr 1984 91 85 ALMERIA 10 Apr 1984 EE127
Birth JOSEFINA
24 Jul 1984
Death
[Death by: Unknown means]

128 M 16 Apr 1984 95 96 ALMERIA 16 Apr 1984 EE128
Birth MANOLO
12 Nov 1985
Death
[Death by: Unknown means]

129 M 16 Apr 1984 97 103 ALMERIA 16 Apr 1984 EE129
Birth SANTI
27 Jul 1991
Death
[Death by: Unknown means]

130 F 23 Apr 1984 95 101 ALMERIA 23 Apr 1984 EE130
Birth BASILISA
22 May 1993
Death
[Death by: Unknown means]

131 F 25 Apr 1984 91 110 ALMERIA 25 Apr 1984 EE131

You created this PDF from an application that is not licensed to print to novaPDF printer (http://www.novapdf.com)

Birth ACACIA
17 Dec 1996
Death
[Death by: Unknown means]

132 M 26 Apr 1984 95 7 ALMERIA 26 Apr 1984 EE132
Birth MARCELINO
11 Nov 1985
Death
[Death by: Unknown means]

133 M 13 May 1984 97 53 ALMERIA 13 May 1984 EE133
Birth LOPEZ
19 Jan 1986
Death
[Death by: Unknown means]

134 M 13 May 1984 97 53 ALMERIA 13 May 1984 EE134
Birth FACAL
18 May 1984
Death
[Death by: Unknown means]

135 F 1 Jun 1984 91 89 ALMERIA 1 Jun 1984 EE135
Birth ARTIFICIAL
5 Jun 1984
Death
[Death by: Unknown means]

136 F 1 Sep 1984 91 71 ALMERIA 1 Sep 1984 EE136
Birth MAXIMA
25 Feb 1986
Death
[Death by: Unknown means]

137 M 18 Sep 1984 91 86 ALMERIA 18 Sep 1984 EE137
Birth PERDIDO
20 Sep 1984
Death
[Death by: Unknown means]

138 M 13 Oct 1984 95 69 ALMERIA 13 Oct 1984 EE138
Birth EDUARDO
20 Jan 1986
Death
[Death by: Unknown means]

139 M 29 Oct 1984 97 20 ALMERIA 29 Oct 1984 EE139
Birth SIMON
30 Jul 1985
Death
[Death by: Unknown means]

140 M 8 Nov 1984 95 94 ALMERIA 8 Nov 1984 EE140
Birth GODOFREDO
26 Aug 1988
Death
[Death by: Unknown means]

141 F 18 Nov 1984 91 80 ALMERIA 18 Nov 1984 EE141
Birth SN
19 Nov 1984
Death
[Death by: Unknown means]

142 F 27 Nov 1984 91 85 ALMERIA 27 Nov 1984 EE142
Birth ANDREA
19 Jul 1991
Death

You created this PDF from an application that is not licensed to print to novaPDF printer (http://www.novapdf.com)

[Death by: Unknown means]

143 F 22 Feb 1985 97 103 ALMERIA 22 Feb 1985 EE143
Birth LEONOR
24 Mar 1985
Death
[Death by: Unknown means]

144 M 26 Feb 1985 97 118 ALMERIA 26 Feb 1985 EE144
Birth JUSTO
28 Feb 1985
Death
[Death by: Unknown means]

145 F 26 Feb 1985 97 118 ALMERIA 26 Feb 1985 EE145
Birth RUFINA
28 Feb 1985
Death
[Death by: Unknown means]

146 M 22 Mar 1985 91 110 ALMERIA 22 Mar 1985 EE146
Birth BIENVENIDO
25 Jun 1986
Death
[Death by: Unknown means]

147 M 23 Mar 1985 95 101 ALMERIA 23 Mar 1985 EE147
Birth TORIBIO
25 May 1987
Death
[Death by: Unknown means]

148 M 24 Mar 1985 95 7 ALMERIA 24 Mar 1985 EE148
Birth AGAPITO
24 Jan 1992
Death
[Death by: Unknown means]

149 F 24 Mar 1985 95 7 ALMERIA 24 Mar 1985 EE149
Birth ANUNCIA
23 May 1989
Death
[Death by: Unknown means]

150 M 28 Mar 1985 97 53 ALMERIA 28 Mar 1985 EE150
Birth BRAULIO
5 Jun 1985
Death
[Death by: Unknown means]

151 M 28 Mar 1985 97 53 ALMERIA 28 Mar 1985 EE151
Birth RUPERTO
8 Jun 1985
Death
[Death by: Unknown means]

152 M 29 Mar 1985 91 71 ALMERIA 29 Mar 1985 EE152
Birth SIXTO
8 Jul 1990
Death
[Death by: Unknown means]

153 M 29 Mar 1985 91 71 ALMERIA 29 Mar 1985 EE153
Birth JONAS
5 Jul 1987
Death
[Death by: Unknown means]

154 M 7 Apr 1985 95 69 ALMERIA 7 Apr 1985 EE154

You created this PDF from an application that is not licensed to print to novaPDF printer (http://www.novapdf.com)

Birth MARCELO
11 Oct 1999
Death
[Death by: Unknown means]

155 M 7 Apr 1985 95 69 ALMERIA 7 Apr 1985 EE155
Birth SN
10 Apr 1985
Death
[Death by: Unknown means]

156 F 15 Apr 1985 95 122 ALMERIA 15 Apr 1985 EE156
Birth BENDITA
13 Apr 1986
Death
[Death by: Unknown means]

157 F 9 May 1985 91 119 ALMERIA 9 May 1985 EE157
Birth SE¥ORA
26 Sep 1985
Death
[Death by: Unknown means]

158 M 16 May 1985 91 80 ALMERIA 16 May 1985 EE158
Birth ESCOS
13 Jul 2001
Death
[Death by: Unknown means]

159 F 16 May 1985 91 80 ALMERIA 16 May 1985 EE159
Birth INMA
6 Aug 1999
Death
[Death by: Unknown means]

160 F 31 May 1985 95 94 ALMERIA 31 May 1985 EE160
Birth PRIMAVERA
14 Oct 1986
Death
[Death by: Unknown means]

161 M 6 Jul 1985 91 86 ALMERIA 6 Jul 1985 EE161
Birth JESUS
9 May 1987
Death
[Death by: Unknown means]

162 M 15 Jul 1985 91 89 ALMERIA 15 Jul 1985 EE162
Birth SN
15 Jul 1985
Death
[Death by: Unknown means]

163 M 1 Nov 1985 91 71 ALMERIA 1 Nov 1985 EE163
Birth GENIO
5 Jun 1988
Death
[Death by: Unknown means]

164 M 7 Nov 1985 97 53 ALMERIA 7 Nov 1985 EE164
Birth FELIPE
17 Mar 1988
Death
[Death by: Unknown means]

165 M 7 Nov 1985 97 53 ALMERIA 7 Nov 1985 EE165
Birth ALFONSO
27 Apr 1989
Death

You created this PDF from an application that is not licensed to print to novaPDF printer (http://www.novapdf.com)

[Death by: Unknown means]

166 F 18 Nov 1985 97 103 ALMERIA 18 Nov 1985 EE166
Birth LLUVIA
17 Apr 1991
Death
[Death by: Unknown means]

167 F 1 Dec 1985 95 101 ALMERIA 1 Dec 1985 EE167
Birth PASTORA
3 Feb 1999
Death
[Death by: Unknown means]

168 M 7 Jan 1986 95 94 ALMERIA 7 Jan 1986 EE168
Birth RAIMUNDO
27 Mar 1989
Death
[Death by: Unknown means]

169 M 14 Feb 1986 91 80 ALMERIA 14 Feb 1986 EE169
Birth VALENTIN
27 Jan 1991
Death
[Death by: Unknown means]

170 F 14 Feb 1986 91 80 ALMERIA 14 Feb 1986 EE170
Birth ENAMORADA
20 Mar 1994
Death
[Death by: Unknown means]

171 M 17 Feb 1986 95 69 ALMERIA 17 Feb 1986 EE171
Birth NEPTUNO
9 Jul 1987
Death
[Death by: Unknown means]

172 M 17 Feb 1986 95 69 ALMERIA 17 Feb 1986 EE172
Birth PLUTON
12 Jun 1987
Death
[Death by: Unknown means]

173 M 17 Feb 1986 95 7 ALMERIA 17 Feb 1986 EE173
Birth SN
17 Feb 1986
Death
[Death by: Unknown means]

174 F 17 Feb 1986 95 7 ALMERIA 17 Feb 1986 EE174
Birth LUNA
26 Nov 1990
Death
[Death by: Unknown means]

175 M 1 Mar 1986 95 122 ALMERIA 1 Mar 1986 EE175
Birth LEO
17 Jun 1989
Death
[Death by: Unknown means]

176 M 1 Mar 1986 95 122 ALMERIA 1 Mar 1986 EE176
Birth TAURO
28 Jul 1987
Death
[Death by: Unknown means]

177 F 5 Mar 1986 17 110 ALMERIA 5 Mar 1986 EE177

You created this PDF from an application that is not licensed to print to novaPDF printer (http://www.novapdf.com)

```
Birth     LIBRA
                                                           14 May 1989
Death
                           [Death by: Unknown means]

       178   F     5 Mar 1986      17      110  ALMERIA     5 Mar 1986  EE178
Birth     VIRGO
                                                           18 Jun 1986
Death
                           [Death by: Unknown means]

       179   M     8 Mar 1986      91       86  ALMERIA     8 Mar 1986  EE179
Birth     CHAPARRON
                                                           10 Mar 1986
Death
                           [Death by: Unknown means]

       180   F    19 Mar 1986      17      119  ALMERIA    19 Mar 1986  EE180
Birth     SN
                                                           20 Mar 1986
Death
                           [Death by: Unknown means]

       181   F    19 Mar 1986      17      119  ALMERIA    19 Mar 1986  EE181
Birth     HUERFANA
                                                           27 Mar 1986
Death
                           [Death by: Unknown means]

       182   F    22 Mar 1986      17      118  ALMERIA    22 Mar 1986  EE182
Birth     ROCIO
                                                           30 Sep 1991
Death
                           [Death by: Unknown means]

       183   F    22 Mar 1986      17      118  ALMERIA    22 Mar 1986  EE183
Birth     MARISMA
                                                MADRID Z    4 Jul 1990    UNK
Transfer
                                                           29 Jul 1990
Death
                           [Death by: Unknown means]

       184   M    12 Apr 1986      95      156  ALMERIA    12 Apr 1986  EE184
Birth     SN
                                                           12 Apr 1986
Death
                           [Death by: Unknown means]

       185   F    12 Apr 1986      95      156  ALMERIA    12 Apr 1986  EE185
Birth     SN
                                                           12 Apr 1986
Death
                           [Death by: Unknown means]

       186   M    12 Apr 1986     125      130  ALMERIA    12 Apr 1986  EE186
Birth     JULIO
                                                           20 Apr 1986
Death
                           [Death by: Unknown means]

       187   F    12 Apr 1986     125      130  ALMERIA    12 Apr 1986  EE187
Birth     JULIA
                                                            2 Aug 1986
Death
                           [Death by: Unknown means]

       188   M    16 Apr 1986      95      149  ALMERIA    16 Apr 1986  EE188
Birth     JULIAN
```


You created this PDF from an application that is not licensed to print to novaPDF printer (http://www.novapdf.com)

```
                                                          18 Apr 1986
Death
                           [Death by: Unknown means]

      189   M    30 Apr 1986      97       53  ALMERIA    30 Apr 1986  EE189
Birth     PIO
                                                           5 Apr 1989
Death
                           [Death by: Unknown means]

      190   F    30 Apr 1986      97       53  ALMERIA    30 Apr 1986  EE190
Birth     PIA
                                                          16 Jul 1986
Death
                           [Death by: Unknown means]

      191   F       ????        WILD     WILD  MARRAKECH     ????        UNK
Capture   MINA
                                               ALMER.CTY     ????        UNK
Transfer
                                                           6 Dec 1985
Death
                           [Death by: Unknown means]

      192   M    21 Mar 1984     UNK     1248  MUNICH     21 Mar 1984     28
Birth     PESAO
                                               SANDIEGOZ  31 Dec 1985 030391
Transfer
                                                          27 Sep 1999
Death
                           [Death by: Unknown means]

      193   M    21 Mar 1984     UNK     1248  MUNICH     21 Mar 1984     27
Birth     MANUEL
                                               SANDIEGOZ  31 Dec 1985 030392
Transfer
                                                          18 Dec 1987
Death
                           [Death by: Unknown means]

      194   F     6 Apr 1984     UNK     1243  MUNICH      6 Apr 1984     29
Birth     CORTINA
                                               SANDIEGOZ  31 Dec 1985 030393
Transfer
                                                          24 Jul 1993
Death
                           [Death by: Unknown means]

      195   F       ????        WILD     WILD  MARRAKECH     ????        UNK
Capture   SN
                                               RABAT      13 Feb 1988
Death
                           [Death by: Unknown means]

      196   F       ????        WILD     WILD  MARRAKECH     ????        UNK
Capture   SN

      197   F     7 Jul 1982      36      191  ALMER.CTY   7 Jul 1982 CASS02
Birth     CASSI02
                                                           1 Jan 1986
Death
                           [Death by: Unknown means]

      198   M     6 Apr 1983      36      191  ALMER.CTY   6 Apr 1983 CASI03
Birth     BATALLON
                                                          25 Feb 1994
Death
                           [Death by: Unknown means]
```


You created this PDF from an application that is not licensed to print to novaPDF printer (http://www.novapdf.com)

199 M 20 Apr 1985 198 197 ALMER.CTY 20 Apr 1985 CASI04
Birth RABAT
ALMERIA 14 May 1996
Death
[Death by: Unknown means]

200 M 20 Apr 1985 198 197 ALMER.CTY 20 Apr 1985 CASI05
Birth TAZA
ALMERIA 20 Dec 1993
Death
[Death by: Unknown means]

201 M 10 May 1986 91 71 ALMERIA 10 May 1986 EE201
Birth RAMON
20 Jun 1988
Death
[Death by: Unknown means]

202 F 10 May 1986 91 71 ALMERIA 10 May 1986 EE202
Birth PILAR
29 Jul 1991
Death
[Death by: Unknown means]

203 M 18 May 1986 97 103 ALMERIA 18 May 1986 EE203
Birth BERNARDO
3 Oct 1998
Death
[Death by: Unknown means]

204 F 27 Jun 1986 95 101 ALMERIA 27 Jun 1986 EE204
Birth PAZ
25 Feb 1994
Death
[Death by: Unknown means]

205 M 10 Aug 1986 125 94 ALMERIA 10 Aug 1986 EE205
Birth LORENZO
14 Nov 1986
Death
[Death by: Unknown means]

206 M 14 Aug 1986 192 194 SANDIEGOZ 14 Aug 1986 031687
Birth JEAN PIERRE
PALM DES 5 Jun 1987 48774
Transfer
10 Feb 1996
Death
[Death by: Other/Unknown + Unknown + Generalized +
Mechanical abnormality]

207 M 25 Sep 1986 91 86 ALMERIA 25 Sep 1986 EE207
Birth AURELIO
3 Dec 1987
Death
[Death by: Unknown means]

208 F 10 Oct 1986 91 80 ALMERIA 10 Oct 1986 EE208
Birth PAQUITA
20 Dec 1999
Death
[Death by: Unknown means]

209 M 30 Oct 1986 95 7 ALMERIA 30 Oct 1986 EE209
Birth ROMEO
5 Nov 1986
Death
[Death by: Unknown means]

You created this PDF from an application that is not licensed to print to novaPDF printer (http://www.novapdf.com)

```
        210   F    30 Oct 1986      95       7  ALMERIA    30 Oct 1986  EE210
Birth      JULIETA
                                                            1 Sep 1989
Death
                            [Death by: Unknown means]

        211   F    12 Dec 1986      98     110  ALMERIA    12 Dec 1986  EE211
Birth      CELOSA
                                                           10 Mar 1987
Death
                            [Death by: Unknown means]

        212   M    29 Jan 1987      91      71  ALMERIA    29 Jan 1987  EE212
Birth      YUSUF
                                                           17 Mar 1997
Death
                            [Death by: Unknown means]

        213   F    29 Jan 1987      91      71  ALMERIA    29 Jan 1987  EE213
Birth      ALANA
                                                           10 Mar 1987
Death
                            [Death by: Unknown means]

        214   M     4 Feb 1987      97     103  ALMERIA     4 Feb 1987  EE214
Birth      SIDI
                                                           27 Nov 1987
Death
                            [Death by: Unknown means]

        215   F     4 Feb 1987      97     103  ALMERIA     4 Feb 1987  EE215
Birth      NAMNA
                                                           24 Mar 1996
Death
                            [Death by: Unknown means]

        216   m    30 Nov 1986      95     122  ALMER.CTY  30 Nov 1986 CASI06
Birth      SN
                                                            5 Mar 1988
Death
                            [Death by: Unknown means]

        217   F    30 Nov 1986      95     122  ALMER.CTY  30 Nov 1986 CASI07
Birth      SN
                                                           30 Nov 1986
Death
                            [Death by: Unknown means]

        218   M     2 Feb 1987      95     101  ALMER.CTY   2 Feb 1987 CASI08
Birth      SN
                                                            4 Feb 1987
Death
                            [Death by: Unknown means]

        219   F     2 Feb 1987      95     101  ALMER.CTY   2 Feb 1987 CASI09
Birth      SN
                                                           25 Feb 1989
Death
                            [Death by: Unknown means]

        220   M    12 Mar 1987      95     149  ALMERIA    12 Mar 1987  EE220
Birth      LASHEN
                                                           29 Mar 1990
Death
                            [Death by: Unknown means]

        221   F    12 Mar 1987      95     149  ALMERIA    12 Mar 1987  EE221
Birth      AISHA
                                                           24 Mar 1998
```


You created this PDF from an application that is not licensed to print to novaPDF printer (http://www.novapdf.com)

Death
[Death by: Unknown means]

222 F 13 Mar 1987 97 166 ALMERIA 13 Mar 1987 EE222
Birth SN
17 Mar 1987
Death
[Death by: Unknown means]

223 M 13 Mar 1987 97 166 ALMERIA 13 Mar 1987 EE223
Birth SN
16 Mar 1987
Death
[Death by: Unknown means]

224 F 16 Mar 1987 98 118 ALMERIA 16 Mar 1987 EE224
Birth FANIDA
11 Nov 1993
Death
[Death by: Unknown means]

225 F 16 Mar 1987 98 118 ALMERIA 16 Mar 1987 EE225
Birth SN
17 Mar 1987
Death
[Death by: Unknown means]

226 M 25 Mar 1987 95 174 ALMERIA 25 Mar 1987 EE226
Birth BASHIR
3 Jan 1990
Death
[Death by: Unknown means]

227 F 30 Mar 1987 98 177 ALMERIA 30 Mar 1987 EE227
Birth SAINABU
18 Mar 1996
Death
[Death by: Unknown means]

228 F 31 Mar 1987 125 131 ALMERIA 31 Mar 1987 EE228
Birth SAGUIRA
16 Apr 2001
Death
[Death by: Unknown means]

229 F 31 Mar 1987 125 131 ALMERIA 31 Mar 1987 EE229
Birth SGUYERA
30 Mar 1992
Death
[Death by: Unknown means]

230 M 5 Apr 1987 95 167 ALMERIA 5 Apr 1987 EE230
Birth HAMMUDI
31 May 1989
Death
[Death by: Unknown means]

231 F 6 Apr 1987 98 183 ALMERIA 6 Apr 1987 EE231
Birth FATIMA
MADRID Z 4 Jul 1990 UNK
Transfer
6 Feb 1994
Death
[Death by: Unknown means]

232 M 22 Apr 1987 199 86 ALMERIA 22 Apr 1987 EE232
Birth SAKEN
16 Dec 1996
Death

You created this PDF from an application that is not licensed to print to novaPDF printer (http://www.novapdf.com)

[Death by: Unknown means]

233 F 27 Apr 1987 98 182 ALMERIA 27 Apr 1987 EE233
Birth NANNUHA
15 Jul 1991
Death
[Death by: Unknown means]

234 M 1 May 1987 199 142 ALMERIA 1 May 1987 EE234
Birth SN
4 May 1987
Death
[Death by: Unknown means]

235 M 11 May 1987 199 80 ALMERIA 11 May 1987 EE235
Birth SALEK
4 Jun 1988
Death
[Death by: Unknown means]

236 F 11 May 1987 199 80 ALMERIA 11 May 1987 EE236
Birth SALKA
5 May 1995
Death
[Death by: Unknown means]

237 F 19 May 1987 95 7 ALMERIA 19 May 1987 EE237
Birth GRARA
9 Apr 2000
Death
[Death by: Unknown means]

238 F 19 May 1987 95 7 ALMERIA 19 May 1987 EE238
Birth SEBKA
31 Jul 1992
Death
[Death by: Unknown means]

239 M 8 Jun 1987 98 110 ALMERIA 8 Jun 1987 EE239
Birth SN
10 Jun 1987
Death
[Death by: Unknown means]

240 F 8 Jun 1987 98 110 ALMERIA 8 Jun 1987 EE240
Birth MEDINA
17 Jun 1987
Death
[Death by: Unknown means]

241 F 5 Jun 1987 198 122 ALMER.CTY 5 Jun 1987 CASI10
Birth SN
25 Feb 1994
Death
[Death by: Unknown means]

242 F 18 Aug 1987 97 103 ALMERIA 18 Aug 1987 EE242
Birth AGBA
29 Mar 1995
Death
[Death by: Unknown means]

243 M 18 Feb 1987 192 194 SANDIEGOZ 18 Feb 1987 587020
Birth
SD-WAP 17 Sep 1987 587020
Transfer
21 Sep 1997
Death
[Death by: Unknown means]

You created this PDF from an application that is not licensed to print to novaPDF printer (http://www.novapdf.com)

244 F 18 Feb 1987 192 194 SANDIEGOZ 18 Feb 1987 587021
Birth COLETTE
PALM DES 17 Nov 1987 487114
Loan to
OR WILDLF 3 Jun 1994 87290
Loan to
18 Dec 2002
Death
[Death by: Unknown means]

245 M 28 Aug 1987 192 194 SANDIEGOZ 28 Aug 1987 587294
Birth
HOLIDAY 18 Apr 1988 ______
Transfer
10 Jan 1990
Death
[Death by: Unknown means]

246 F 28 Aug 1987 192 194 SANDIEGOZ 28 Aug 1987 587295
Birth
HOLIDAY 18 Apr 1988 ______
Transfer
13 Nov 1988
Death
[Death by: Unknown means]

247 F 9 Oct 1987 200 118 ALMERIA 9 Oct 1987 EE247
Birth TUAMA
6 Mar 1992
Death
[Death by: Unknown means]

248 M 10 Oct 1981 UNK 66 MUNICH 10 Oct 1981 12
Birth MANOLO
SANDIEGOZ 15 Sep 1987 587313
Transfer
22 Mar 1989
Death
[Death by: Unknown means]

249 F 8 Sep 1983 UNK 68 MUNICH 8 Sep 1983 25
Birth CARMEN
SANDIEGOZ 15 Sep 1987 587315
Transfer
ST LOUIS 5 Oct 1989 089137
Transfer
27 May 1992
Death
[Death by: Unknown means]

250 M 20 Jul 1985 248 68 MUNICH 20 Jul 1985 UNK
Birth LUCIANO
SANDIEGOZ 15 Sep 1987 587312
Transfer
ST LOUIS 5 Oct 1989 089136
Transfer
8 Aug 1991
Death
[Death by: Unknown means]

251 F 28 Feb 1986 UNK 22 MUNICH 28 Feb 1986 UNK
Birth ROSALIA
SANDIEGOZ 15 Sep 1987 587317
Transfer
4 Aug 1994
Death
[Death by: Euthanasia (medical) + Unknown + Reproductive + Mechanical abnormality]

You created this PDF from an application that is not licensed to print to novaPDF printer (http://www.novapdf.com)

252 F 14 Dec 1986 248 1243 MUNICH 14 Dec 1986 UNK
Birth CHARLOTTA
SANDIEGOZ 15 Sep 1987 587316
Transfer
13 Jan 1999
Death
[Death by: Unknown means]

253 F 16 Nov 1987 199 166 ALMERIA 16 Nov 1987 EE253
Birth HASSENA
25 Jul 1995
Death
[Death by: Unknown means]

254 F 29 Nov 1987 200 183 ALMERIA 29 Nov 1987 EE254
Birth TAAMAT
MADRID Z 4 Jul 1990 UNK
Transfer
22 Dec 1991
Death
[Death by: Unknown means]

255 F 19 Feb 1988 200 177 ALMERIA 19 Feb 1988 EE255
Birth ALCAZABA
26 Nov 1999
Death
[Death by: Unknown means]

256 M 2 Mar 1988 199 103 ALMERIA 2 Mar 1988 EE256
Birth TASSILI
9 Jul 2003
Death
[Death by: Unknown means]

257 F 12 Jan 1988 198 122 ALMER.CTY 12 Jan 1988 CASI11
Birth SN
25 Feb 1989
Death
[Death by: Unknown means]

258 F 12 Jan 1988 198 122 ALMER.CTY 12 Jan 1988 CASI12
Birth SN
4 Jun 1999
Death
[Death by: Unknown means]

259 M ???? UNK UNK MARRAKECH ???? MA259
Birth SN

260 M 21 Mar 1988 199 215 ALMERIA 21 Mar 1988 EE260
Birth ARBOL
25 Mar 1988
Death
[Death by: Unknown means]

261 F 8 Apr 1988 200 224 ALMERIA 8 Apr 1988 EE261
Birth TANGA
25 Oct 1997
Death
[Death by: Unknown means]

262 M 18 Apr 1988 200 231 ALMERIA 18 Apr 1988 EE262
Birth DJEBEL
10 Apr 1989
Death
[Death by: Unknown means]

263 F 18 Apr 1988 200 231 ALMERIA 18 Apr 1988 EE263

You created this PDF from an application that is not licensed to print to novaPDF printer (http://www.novapdf.com)

Birth DJEBAL
MADRID Z 4 Jul 1990 UNK
Transfer
4 Oct 1991
Death
[Death by: Unknown means]

264 M 10 May 1988 199 166 ALMERIA 10 May 1988 EE264
Birth JALIL
MADRID Z 4 Jul 1990 UNK
Transfer
ALMERIA ???? EE264
Transfer
15 Mar 1995
Death
[Death by: Unknown means]

265 F 10 May 1988 199 166 ALMERIA 10 May 1988 EE265
Birth JALINA
25 Jan 1996
Death
[Death by: Unknown means]

266 F 13 May 1988 200 110 ALMERIA 13 May 1988 EE266
Birth NURIA
28 May 1996
Death
[Death by: Unknown means]

267 F 15 May 1988 200 227 ALMERIA 15 May 1988 EE267
Birth ALIMA
26 Dec 1989
Death
[Death by: Unknown means]

268 F 30 May 1988 200 182 ALMERIA 30 May 1988 EE268
Birth NIBLA
17 Oct 1997
Death
[Death by: Unknown means]

269 M 7 Sep 1988 200 183 ALMERIA 7 Sep 1988 EE269
Birth MEMBRILLO
24 Sep 1993
Death
[Death by: Unknown means]

270 F 20 Sep 1988 200 177 ALMERIA 20 Sep 1988 EE270
Birth ALBA
6 Mar 1995
Death
[Death by: Unknown means]

271 F 25 Sep 1988 199 103 ALMERIA 25 Sep 1988 EE271
Birth GUERA
9 May 2001
Death
[Death by: Unknown means]

272 M 28 Jan 1989 200 110 ALMERIA 28 Jan 1989 EE272
Birth JOAQUIN
19 Aug 1998
Death
[Death by: Unknown means]

273 F 1 Feb 1989 200 182 ALMERIA 1 Feb 1989 EE273
Birth ANGELA
2 Nov 1997
Death

You created this PDF from an application that is not licensed to print to novaPDF printer (http://www.novapdf.com)

[Death by: Unknown means]

274 F 1 Feb 1989 200 182 ALMERIA 1 Feb 1989 EE274
Birth ROSA
29 Jun 1995
Death
[Death by: Unknown means]

275 M 16 Mar 1988 UNK UNK RABAT 16 Mar 1988 RA275
Birth SN

276 F 16 Mar 1988 UNK UNK RABAT 16 Mar 1988 RA276
Birth SN

277 M 18 Jul 1988 UNK UNK RABAT 18 Jul 1988 RA277
Birth SN

278 M 24 Feb 1989 199 215 ALMERIA 24 Feb 1989 EE278
Birth TEMPORAL
31 Mar 2002
Death
[Death by: Unknown means]

279 F 24 Feb 1989 199 215 ALMERIA 24 Feb 1989 EE279
Birth TORMENTA
26 Jan 1995
Death
[Death by: Unknown means]

280 F 13 Mar 1989 199 242 ALMERIA 13 Mar 1989 EE280
Birth HADYA
13 Nov 2003
Death
[Death by: Unknown means]

281 F 22 Mar 1989 199 253 ALMERIA 22 Mar 1989 EE281
Birth ALBAHACA
2 Feb 1993
Death
[Death by: Unknown means]

282 F 22 Mar 1989 199 253 ALMERIA 22 Mar 1989 EE282
Birth SIRA
10 Jun 1990
Death
[Death by: Unknown means]

283 F 26 Mar 1989 199 103 ALMERIA 26 Mar 1989 EE283
Birth NEBKA
4 Jan 1990
Death
[Death by: Unknown means]

284 F 26 Mar 1989 199 103 ALMERIA 26 Mar 1989 EE284
Birth GRAD
2 Oct 1997
Death
[Death by: Unknown means]

285 M 1 May 1989 199 265 ALMERIA 1 May 1989 EE285
Birth OBRERO
4 May 1989
Death
[Death by: Unknown means]

286 M 20 Jun 1988 198 204 ALMER.CTY 20 Jun 1988 CASI13
Birth SN
17 Sep 1989
Death

You created this PDF from an application that is not licensed to print to novaPDF printer (http://www.novapdf.com)

[Death by: Unknown means]

287 F 8 Aug 1988 198 122 ALMER.CTY 8 Aug 1988 CASI14
Birth SN
18 Aug 1989
Death
[Death by: Unknown means]

288 F 1 Jan 1988 198 204 ALMER.CTY 1 Jan 1988 CASI15
Birth LAGARTIJA
25 Feb 1994
Death
[Death by: Unknown means]

289 M 16 Mar 1989 198 122 ALMER.CTY 16 Mar 1989 CASI16
Birth SN
25 Feb 1994
Death
[Death by: Unknown means]

290 M 16 Mar 1989 198 122 ALMER.CTY 16 Mar 1989 CASI17
Birth SN
ALMERIA 20 Aug 1997
Death
[Death by: Unknown means]

291 F 9 Apr 1989 198 204 ALMER.CTY 9 Apr 1989 CASI18
Birth TEMPRANA
25 Feb 1994
Death
[Death by: Unknown means]

292 M 2 May 1989 198 258 ALMER.CTY 2 May 1989 CASI19
Birth SN
25 Feb 1994
Death
[Death by: Unknown means]

293 F 9 Apr 1989 198 204 ALMERIA 9 Apr 1989 CASI20
Birth IMPROVISA
25 Feb 1994
Death
[Death by: Unknown means]

294 M 2 Oct 1989 199 166 ALMERIA 2 Oct 1989 EE294
Birth ILIF
11 Oct 1989
Death
[Death by: Unknown means]

295 F 2 Oct 1989 199 166 ALMERIA 2 Oct 1989 EE295
Birth ILIFA
4 Oct 1989
Death
[Death by: Unknown means]

296 M 6 Oct 1989 199 215 ALMERIA 6 Oct 1989 EE296
Birth SN
7 Oct 1989
Death
[Death by: Unknown means]

297 M 6 Oct 1989 199 215 ALMERIA 6 Oct 1989 EE297
Birth SN
9 Oct 1989
Death
[Death by: Unknown means]

298 M 12 Nov 1990 264 166 ALMERIA 12 Nov 1990 EE298

You created this PDF from an application that is not licensed to print to novaPDF printer (http://www.novapdf.com)

Birth TINO
17 Sep 1995
Death
[Death by: Unknown means]

299 F 2 Feb 1991 114 202 ALMERIA 2 Feb 1991 EE299
Birth SN
2 Feb 1991
Death
[Death by: Unknown means]

300 M 5 Feb 1989 UNK UNK RABAT 5 Feb 1989 RA300
Birth

301 F 5 Feb 1989 UNK UNK RABAT 5 Feb 1989 RA301
Birth

302 F 29 Mar 1989 UNK UNK RABAT 29 Mar 1989 RA302
Birth

303 F 29 Mar 1989 UNK UNK RABAT 29 Mar 1989 RA303
Birth

304 F 11 Mar 1990 UNK UNK RABAT 11 Mar 1990 RA304
Birth
31 Aug 1990
Death
[Death by: Unknown means]

305 F 30 Apr 1990 UNK UNK RABAT 30 Apr 1990 RA305
Birth

306 F 11 Feb 1991 114 85 ALMERIA 11 Feb 1991 EE306
Birth CAZORLA
18 Aug 1991
Death
[Death by: Unknown means]

307 M 19 Feb 1991 114 208 ALMERIA 19 Feb 1991 EE307
Birth SN
20 Feb 1991
Death
[Death by: Unknown means]

308 M 19 Feb 1991 114 208 ALMERIA 19 Feb 1991 EE308
Birth COSME
24 Oct 1996
Death
[Death by: Unknown means]

309 M 22 Feb 1991 114 86 ALMERIA 22 Feb 1991 EE309
Birth LOBITO
13 Nov 1991
Death
[Death by: Unknown means]

310 F 4 Mar 1991 114 159 ALMERIA 4 Mar 1991 EE310
Birth GUIDA
10 Jul 1991
Death
[Death by: Unknown means]

311 M 4 Mar 1991 114 159 ALMERIA 4 Mar 1991 EE311
Birth SN
7 Mar 1991
Death
[Death by: Unknown means]

312 F 7 Mar 1991 129 221 ALMERIA 7 Mar 1991 EE312

You created this PDF from an application that is not licensed to print to novaPDF printer (http://www.novapdf.com)

Birth SN
8 Mar 1991
Death
[Death by: Unknown means]

313 M 10 Mar 1991 114 170 ALMERIA 10 Mar 1991 EE313
Birth COLICO
17 Jul 1998
Death
[Death by: Unknown means]

314 M 18 Mar 1991 129 229 ALMERIA 18 Mar 1991 EE314
Birth SEBAS
20 Jun 1992
Death
[Death by: Unknown means]

315 M 19 Mar 1991 129 228 ALMERIA 19 Mar 1991 EE315
Birth JOSE
22 Jul 1991
Death
[Death by: Unknown means]

316 M 19 Mar 1991 129 228 ALMERIA 19 Mar 1991 EE316
Birth SN
20 Mar 1991
Death
[Death by: Unknown means]

317 M 22 Mar 1991 114 236 ALMERIA 22 Mar 1991 EE317
Birth ANDRES
15 Nov 1995
Death
[Death by: Unknown means]

318 F 22 Mar 1991 114 236 ALMERIA 22 Mar 1991 EE318
Birth SN
27 Mar 1991
Death
[Death by: Unknown means]

319 M 3 May 1991 114 142 ALMERIA 3 May 1991 EE319
Birth SN
4 May 1991
Death
[Death by: Unknown means]

320 M 13 Jul 1991 129 237 ALMERIA 13 Jul 1991 EE320
Birth SN
13 Jul 1991
Death
[Death by: Unknown means]

321 F 28 Jul 1991 129 167 ALMERIA 28 Jul 1991 EE321
Birth ENARA
13 Jun 2004
Death
[Death by: Unknown means]

322 F 1 Aug 1991 129 131 ALMERIA 1 Aug 1991 EE322
Birth ZAIDA
BOUKORNIN 13 Dec 1999 EE322
Transfer
18 Nov 2002
Death
[Death by: Unknown means]

323 F 1 Aug 1991 129 131 ALMERIA 1 Aug 1991 EE323
Birth ZORAIDA

You created this PDF from an application that is not licensed to print to novaPDF printer (http://www.novapdf.com)

21 Mar 2002
Death
[Death by: Unknown means]

324 F 6 Sep 1991 129 130 ALMERIA 6 Sep 1991 EE324
Birth SN
8 Sep 1991
Death
[Death by: Unknown means]

325 M 3 Dec 1991 114 170 ALMERIA 3 Dec 1991 EE325
Birth SN
5 Dec 1991
Death
[Death by: Unknown means]

326 M 23 Jan 1992 269 215 ALMERIA 23 Jan 1992 EE326
Birth ANDRESO
4 Jul 1998
Death
[Death by: Unknown means]

327 F 17 Feb 1992 269 280 ALMERIA 17 Feb 1992 EE327
Birth TERESA
2 Jun 2000
Death
[Death by: Unknown means]

328 F 17 Feb 1992 269 280 ALMERIA 17 Feb 1992 EE328
Birth ABAIGAR
19 May 1998
Death
[Death by: Unknown means]

329 F 18 Feb 1992 269 242 ALMERIA 18 Feb 1992 EE329
Birth OLGA
11 Apr 1998
Death
[Death by: Unknown means]

330 M 18 Feb 1992 269 242 ALMERIA 18 Feb 1992 EE330
Birth DELGADO
12 Feb 2002
Death
[Death by: Unknown means]

331 F 24 Feb 1992 278 261 ALMERIA 24 Feb 1992 EE331
Birth ISABEL
21 May 1996
Death
[Death by: Unknown means]

332 F 24 Feb 1992 278 261 ALMERIA 24 Feb 1992 EE332
Birth SN
26 Feb 1992
Death
[Death by: Unknown means]

333 M 25 Feb 1992 269 284 ALMERIA 25 Feb 1992 EE333
Birth ANTONIO
11 Jul 1996
Death
[Death by: Unknown means]

334 M 25 Feb 1992 269 284 ALMERIA 25 Feb 1992 EE334
Birth AGUIRRE
1 Mar 1995
Death
[Death by: Unknown means]

You created this PDF from an application that is not licensed to print to novaPDF printer (http://www.novapdf.com)

335 F 25 Feb 1992 278 224 ALMERIA 25 Feb 1992 EE335
Birth PAQUITA
28 Nov 2001
Death
[Death by: Unknown means]

336 M 25 Feb 1992 269 271 ALMERIA 25 Feb 1992 EE336
Birth POLIFEMO
19 Jan 1998
Death
[Death by: Unknown means]

337 F 25 Feb 1992 269 271 ALMERIA 25 Feb 1992 EE337
Birth GALATEA
13 Dec 1992
Death
[Death by: Unknown means]

338 M 27 Feb 1992 269 279 ALMERIA 27 Feb 1992 EE338
Birth MIGUEL
1 Apr 1998
Death
[Death by: Unknown means]

339 M 27 Feb 1992 269 279 ALMERIA 27 Feb 1992 EE339
Birth CUETO
29 Jul 2000
Death
[Death by: Unknown means]

340 M 4 Mar 1992 278 247 ALMERIA 4 Mar 1992 EE340
Birth SN
4 Mar 1992
Death
[Death by: Unknown means]

341 F 4 Mar 1992 278 255 ALMERIA 4 Mar 1992 EE341
Birth PILAR
9 Jun 2000
Death
[Death by: Unknown means]

342 M 4 Mar 1992 278 270 ALMERIA 4 Mar 1992 EE342
Birth SN
25 Mar 1992
Death
[Death by: Unknown means]

343 F 4 Mar 1992 278 270 ALMERIA 4 Mar 1992 EE343
Birth NURIYA
4 Jul 1993
Death
[Death by: Unknown means]

344 M 6 Mar 1992 278 227 ALMERIA 6 Mar 1992 EE344
Birth SN
6 Mar 1992
Death
[Death by: Unknown means]

345 M 6 Mar 1992 278 227 ALMERIA 6 Mar 1992 EE345
Birth JORGE
25 Jan 1996
Death
[Death by: Unknown means]

346 M 26 May 1992 154 253 ALMERIA 26 May 1992 EE346
Birth JUAN

You created this PDF from an application that is not licensed to print to novaPDF printer (http://www.novapdf.com)

22 Oct 2003
Death
[Death by: Unknown means]

347 F 26 May 1992 154 253 ALMERIA 26 May 1992 EE347
Birth JULIA
17 Apr 2004
Death
[Death by: Unknown means]

348 F 11 Aug 1992 269 215 ALMERIA 11 Aug 1992 EE348
Birth TATI
30 Dec 1996
Death
[Death by: Unknown means]

349 F 11 Aug 1992 269 215 ALMERIA 11 Aug 1992 EE349
Birth NAZA
24 Dec 1998
Death
[Death by: Unknown means]

350 F 31 Aug 1992 125 273 ALMERIA 31 Aug 1992 E350
Birth RAMONA
27 Nov 1997
Death
[Death by: Unknown means]

351 F 31 Aug 1992 125 273 ALMERIA 31 Aug 1992 EE351
Birth JUNY
24 May 2000
Death
[Death by: Unknown means]

352 F 25 Jan 1991 264 263 MADRID Z 25 Jan 1991 MA352
Birth DJEBAL
28 Mar 1991
Death
[Death by: Unknown means]

353 F 8 Apr 1991 264 254 MADRID Z 8 Apr 1991 MA353
Birth TAAMAT
10 Apr 1991
Death
[Death by: Unknown means]

354 F 7 May 1991 264 231 MADRID Z 7 May 1991 MA354
Birth SHEREZADE
2 Feb 1996
Death
[Death by: Unknown means]

355 M 17 Jan 1992 264 231 MADRID Z 17 Jan 1992 MA355
Birth HASSAN
22 Jun 2001
Death
[Death by: Unknown means]

356 F 17 Jan 1992 264 231 MADRID Z 17 Jan 1992 MA356
Birth SORAYA
21 Sep 1996
Death
[Death by: Unknown means]

357 M 5 Sep 1992 125 268 ALMERIA 5 Sep 1992 EE357
Birth SN
5 Sep 1992
Death
[Death by: Unknown means]

You created this PDF from an application that is not licensed to print to novaPDF printer (http://www.novapdf.com)

358 F 7 Sep 1992 125 274 ALMERIA 7 Sep 1992 EE358
Birth SN
7 Sep 1992
Death
[Death by: Unknown means]

359 F 10 Sep 1992 256 228 ALMERIA 10 Sep 1992 EE359
Birth SAIOA
BOUKORNIN 13 Dec 1999 EE359
Transfer
4 Jul 2002
Death
[Death by: Unknown means]

360 F 10 Sep 1992 256 228 ALMERIA 10 Sep 1992 EE360
Birth IDOIA
BOUKORNIN 13 Dec 1999 UNK
Transfer
23 Jul 2000
Death
[Death by: Unknown means]

361 F 16 Sep 1992 125 266 ALMERIA 16 Sep 1992 EE361
Birth REMADA
5 Sep 2005
Death
[Death by: Unknown means]

362 F 16 Sep 1992 125 266 ALMERIA 16 Sep 1992 EE362
Birth DHEBA
16 Mar 1998
Death
[Death by: Unknown means]

363 F 21 Sep 1992 256 131 ALMERIA 21 Sep 1992 EE363
Birth DJERBA
BOUKORNIN 13 Dec 1999 EE363
Transfer
15 Nov 2002
Death
[Death by: Unknown means]

364 F 21 Sep 1992 256 131 ALMERIA 21 Sep 1992 EE364
Birth ZERZIS
7 Jan 2008
Death
[Death by: Old age + Incinerate + No necropsy
planned]

365 M 28 Oct 1992 154 236 ALMERIA 28 Oct 1992 EE365
Birth BOLERO
1 Oct 1996
Death
[Death by: Unknown means]

366 M 10 Dec 1992 256 237 ALMERIA 10 Dec 1992 EE366
Birth SN
11 Dec 1992
Death
[Death by: Unknown means]

367 M 30 Sep 1992 264 231 MADRID Z 30 Sep 1992 MA367
Birth MOHAMED
1 Mar 1993
Death
[Death by: Unknown means]

368 F 7 Mar 1993 256 322 ALMERIA 7 Mar 1993 EE368

You created this PDF from an application that is not licensed to print to novaPDF printer (http://www.novapdf.com)

Birth SN
7 Mar 1993
Death
[Death by: Unknown means]

369 M 7 Mar 1993 256 322 ALMERIA 7 Mar 1993 EE369
Birth SN
7 Mar 1993
Death
[Death by: Unknown means]

370 M 17 Mar 1993 125 268 ALMERIA 17 Mar 1993 EE370
Birth IDEADO
25 Dec 2000
Death
[Death by: Unknown means]

371 F 17 Mar 1993 125 268 ALMERIA 17 Mar 1993 EE371
Birth NIEVES
BOUKORNIN 13 Dec 1999 UNK
Transfer
7 Jul 2000
Death
[Death by: Unknown means]

372 M 18 Mar 1993 256 323 ALMERIA 18 Mar 1993 EE372
Birth SN
21 Mar 1993
Death
[Death by: Unknown means]

373 F 18 Mar 1993 256 323 ALMERIA 18 Mar 1993 EE373
Birth SN
22 Mar 1993
Death
[Death by: Unknown means]

374 M 31 Mar 1993 125 273 ALMERIA 31 Mar 1993 EE374
Birth CARLOS
11 Nov 1995
Death
[Death by: Unknown means]

375 M 31 Mar 1993 125 273 ALMERIA 31 Mar 1993 EE375
Birth ALEJANDRO
6 Dec 1997
Death
[Death by: Unknown means]

376 F 5 Apr 1993 125 266 ALMERIA 5 Apr 1993 EE376
Birth MEDEA
BOUKORNIN 13 Dec 1999 UNK
Transfer
18 Jun 2000
Death
[Death by: Unknown means]

377 F 5 Apr 1993 125 266 ALMERIA 5 Apr 1993 EE377
Birth ELECTRA
24 Dec 1997
Death
[Death by: Unknown means]

378 F 13 May 1993 256 228 ALMERIA 13 May 1993 EE378
Birth AITANA
25 Jul 2000
Death
[Death by: Unknown means]

You created this PDF from an application that is not licensed to print to novaPDF printer (http://www.novapdf.com)

379 M 15 May 1993 256 131 ALMERIA 15 May 1993 EE379
Birth APOLO
28 Nov 1996
Death
[Death by: Unknown means]

380 F 15 May 1993 256 131 ALMERIA 15 May 1993 EE380
Birth EGLETA
15 Dec 2003
Death
[Death by: Unknown means]

381 F 21 Jun 1993 125 274 ALMERIA 21 Jun 1993 EE381
Birth SN
21 Jun 1993
Death
[Death by: Unknown means]

382 M 21 Jun 1993 125 274 ALMERIA 21 Jun 1993 EE382
Birth SN
21 Jun 1993
Death
[Death by: Unknown means]

383 M 19 Jul 1993 199 159 ALMERIA 19 Jul 1993 EE383
Birth MAXIMO
30 Jul 1993
Death
[Death by: Unknown means]

384 M 19 Jul 1993 199 159 ALMERIA 19 Jul 1993 EE384
Birth SEGUNDO
25 Jul 1993
Death
[Death by: Unknown means]

385 F 27 Jul 1993 272 167 ALMERIA 27 Jul 1993 EE385
Birth NATALIA
7 Jun 2007
Death
[Death by: Old age + Incinerate + Generalized +
Bacterial]

386 F 27 Jul 1993 272 167 ALMERIA 27 Jul 1993 EE386
Birth JULIANA
12 May 1995
Death
[Death by: Unknown means]

387 F 11 Aug 1993 199 170 ALMERIA 11 Aug 1993 EE387
Birth ANUKE
23 Jun 2003
Death
[Death by: Unknown means]

388 F 12 Aug 1993 272 321 ALMERIA 12 Aug 1993 EE388
Birth MAYA
17 Apr 1994
Death
[Death by: Unknown means]

389 M 24 Aug 1993 199 110 ALMERIA 24 Aug 1993 EE389
Birth SN
6 Sep 1993
Death
[Death by: Unknown means]

390 F 22 Oct 1993 256 323 ALMERIA 22 Oct 1993 EE390
Birth SN

You created this PDF from an application that is not licensed to print to novaPDF printer (http://www.novapdf.com)

13 Mar 2004
Death
[Death by: Unknown means]

391 F 25 Oct 1993 256 237 ALMERIA 25 Oct 1993 EE391
Birth ARIANA
BOUKORNIN 13 Dec 1999 EE391
Transfer
28 Aug 2002
Death
[Death by: Unknown means]

392 F 25 Oct 1993 256 237 ALMERIA 25 Oct 1993 EE392
Birth AOUSIA
30 Apr 2008
Death
[Death by: Old age + Incinerate + Unknown (after necropsy)]

393 F 28 Oct 1993 256 322 ALMERIA 28 Oct 1993 EE393
Birth SN
28 Oct 1993
Death
[Death by: Unknown means]

394 M 25 Apr 1993 264 231 MADRID Z 25 Apr 1993 MA394
Birth ALI BABA
26 Nov 1995
Death
[Death by: Unknown means]

395 F 13 May 1993 264 354 MADRID Z 13 May 1993 MA395
Birth FARAH
15 May 1993
Death
[Death by: Unknown means]

396 M 16 Dec 1993 264 231 MADRID Z 16 Dec 1993 MA396
Birth YUSEF
6 Apr 1995
Death
[Death by: Unknown means]

397 F 18 Feb 1994 114 270 ALMERIA 18 Feb 1994 EE397
Birth SN
13 Dec 2000
Death
[Death by: Unknown means]

398 M 22 Feb 1994 272 167 ALMERIA 22 Feb 1994 EE398
Birth NABEUL
BOUKORNIN 13 Dec 1999 UNK
Transfer
14 Jan 2000
Death
[Death by: Unknown means]

399 M 22 Feb 1994 272 167 ALMERIA 22 Feb 1994 EE399
Birth NABINO
29 Dec 2001
Death
[Death by: Unknown means]

400 F 1 Mar 1994 199 110 ALMERIA 1 Mar 1994 EE400
Birth SN
12 Mar 1994
Death
[Death by: Unknown means]

You created this PDF from an application that is not licensed to print to novaPDF printer (http://www.novapdf.com)

401 F 1 Mar 1994 199 110 ALMERIA 1 Mar 1994 EE401
Birth SN
11 Mar 1994
Death
[Death by: Unknown means]

402 F 25 Apr 1987 248 22 MUNICH 25 Apr 1987 UNK
Birth RACHA
SANDIEGOZ 16 Nov 1988 588432
Transfer
9 Oct 1997
Death
[Death by: Unknown means]

403 M 5 Feb 2008 650 647 LA LAJITA 5 Feb 2008 UNK
Birth
7 Feb 2009
Death
[Death by: Unknown means]

404 M 4 Aug 1994 UNK 251 SANDIEGOZ 4 Aug 1994 594363
Birth
4 Aug 1994
Death
[Death by: Stillbirth + Incinerate + Generalized +
Trauma]

405 M 15 Oct 1994 192 430 SANDIEGOZ 15 Oct 1994 594440
Birth
16 Oct 1994
Death
[Death by: Unknown means]

406 M 9 Nov 1994 192 402 SANDIEGOZ 9 Nov 1994 594455
Birth
9 Nov 1994
Death
[Death by: Unknown means]

407 ? 27 Nov 1994 662 421 SANDIEGOZ 27 Nov 1994 594480
Birth
27 Nov 1994
Death
[Death by: Premature birth + Incinerate + Unknown
(after necropsy)]

408 M 15 Jul 1985 248 1243 MUNICH 15 Jul 1985 UNK
Birth APARECIDO
7 Jun 1986
Death
[Death by: Unknown means]

409 M 1 Mar 1988 192 194 SANDIEGOZ 1 Mar 1988 588086
Birth
4 Mar 1988
Death
[Death by: Unknown means]

410 F 1 Mar 1988 192 194 SANDIEGOZ 1 Mar 1988 588087
Birth
SD-WAP 10 Jan 1989 588087
Transfer
13 Aug 1992
Death
[Death by: Unknown means]

411 M 8 Mar 1988 192 244 PALM DES 8 Mar 1988 48818
Birth
10 Mar 1988

You created this PDF from an application that is not licensed to print to novaPDF printer (http://www.novapdf.com)

Death
[Death by: Unknown means]

412 F 8 Mar 1988 192 244 PALM DES 8 Mar 1988 48817
Birth GISELE
OR WILDLF 5 Dec 1996 88428
Loan to
31 Dec 1996 88428
Transfer
5 Apr 1998
Death
[Death by: Unknown means]

413 F 17 May 1988 192 68 SANDIEGOZ 17 May 1988 588211
Birth
17 May 1988
Death
[Death by: Stillbirth + Unknown + No necropsy
planned]

414 F 17 Jun 1988 192 252 SANDIEGOZ 17 Jun 1988 588241
Birth
19 Jun 1988
Death
[Death by: Unknown means]

415 M 5 Sep 1988 192 194 SANDIEGOZ 5 Sep 1988 588350
Birth ELVIS
ST LOUIS 5 Oct 1989 089135
Transfer
6 Oct 2001
Death
[Death by: Euthanasia (medical) + Unknown + Unknown
(after necropsy)]

416 F 5 Sep 1988 192 194 SANDIEGOZ 5 Sep 1988 588351
Birth
SD-WAP 7 Mar 1989 588351
Transfer
26 Aug 1994
Death
[Death by: Unknown means]

417 M 24 Nov 1988 42 249 SANDIEGOZ 24 Nov 1988 588438
Birth
9 Dec 1988
Death
[Death by: Unknown means]

418 M 24 Nov 1988 42 249 SANDIEGOZ 24 Nov 1988 588439
Birth
12 Dec 1988
Death
[Death by: Unknown means]

419 F 13 Feb 1989 192 194 SANDIEGOZ 13 Feb 1989 589032
Birth
13 Feb 1989
Death
[Death by: Stillbirth + Unknown + No necropsy
planned]

420 F 13 Feb 1989 192 194 SANDIEGOZ 13 Feb 1989 589033
Birth
13 Feb 1989
Death
[Death by: Stillbirth + Unknown + No necropsy
planned]

You created this PDF from an application that is not licensed to print to novaPDF printer (http://www.novapdf.com)

421 F 18 Feb 1989 192 251 SANDIEGOZ 18 Feb 1989 589035
Birth
22 Nov 1998
Death
[Death by: Euthanasia (medical) ┼ Incinerate ┼
Generalized ┼ Bacterial]

422 F 18 Feb 1989 192 251 SANDIEGOZ 18 Feb 1989 589036
Birth PRISCILLA
ST LOUIS 5 Oct 1989 089138
Transfer
2 Sep 1993
Death
[Death by: Unknown means]

423 M 22 Feb 1989 192 252 SANDIEGOZ 22 Feb 1989 589038
Birth
10 Mar 1989
Death
[Death by: Unknown means]

424 F 22 Feb 1989 192 252 SANDIEGOZ 22 Feb 1989 589039
Birth
10 Mar 1989
Death
[Death by: Unknown means]

425 M 4 Oct 1989 192 252 SANDIEGOZ 4 Oct 1989 589366
Birth
6 Oct 1989
Death
[Death by: Unknown means]

426 M 27 Jan 1990 206 244 PALM DES 27 Jan 1990 490001
Birth JEAN CLAUDE
SANDIEGOZ 12 Jun 1990 590298
Transfer
1 Mar 1992
Death
[Death by: Unknown means]

427 M 27 Jan 1990 206 244 PALM DES 27 Jan 1990 490002
Birth ROBAIRE
SANDIEGOZ 12 Jun 1990 590297
Transfer
CINCINNAT 23 Nov 1990 190251
Transfer
WILDS 5 Jun 1992 920601
Loan to
14 Apr 2000
Death
[Death by: Euthanasia (medical) ┼ Bury ┼ Generalized ┼
Bacterial]

428 F 6 Feb 1990 42 194 SANDIEGOZ 6 Feb 1990 590032
Birth
CINCINNAT 23 Nov 1990 190252
Transfer
WILDS 5 Jun 1992 920602
Loan to
10 Sep 1994
Death
[Death by: Unknown means]

429 F 6 Feb 1990 42 194 SANDIEGOZ 6 Feb 1990 590033
Birth GLORIA
CINCINNAT 23 Nov 1990 190253
Transfer
WILDS 5 Jun 1992 920603

You created this PDF from an application that is not licensed to print to novaPDF printer (http://www.novapdf.com)

Loan to
2 Aug 1997
Death
[Death by: Unknown means]

430 F 17 Feb 1990 42 251 SANDIEGOZ 17 Feb 1990 590040
Birth
PALM DES 15 Aug 1995 495028
Loan to
2 Sep 1995
Death
[Death by: Unknown means]

431 F 7 Mar 1990 243 416 SD-WAP 7 Mar 1990 690088
Birth
2 Jul 1990
Death
[Death by: Unknown means]

432 F 7 Mar 1990 243 416 SD-WAP 7 Mar 1990 690089
Birth
13 Oct 1995
Death
[Death by: Unknown means]

433 F 25 Mar 1990 206 412 PALM DES 25 Mar 1990 490006
Birth
7 Jun 1990
Death
[Death by: Unknown means]

434 M 16 Apr 1990 250 249 ST LOUIS 16 Apr 1990 090033
Birth
16 Apr 1990
Death
[Death by: Unknown means]

435 M 16 Apr 1990 250 249 ST LOUIS 16 Apr 1990 090034
Birth
16 Apr 1990
Death
[Death by: Unknown means]

436 M 22 Apr 1990 42 252 SANDIEGOZ 22 Apr 1990 590157
Birth
SD-WAP 18 Mar 1994 590157
Transfer
27 Sep 1995
Death
[Death by: Euthanasia (medical) | Unknown | No necropsy planned]

437 M 22 Apr 1990 42 252 SANDIEGOZ 22 Apr 1990 590158
Birth
HOLIDAY 1 Nov 1991 ______
Transfer
15 Mar 1992
Death
[Death by: Unknown means]

438 M 29 Apr 1990 42 421 SANDIEGOZ 29 Apr 1990 590185
Birth
29 Apr 1990
Death
[Death by: Stillbirth | Unknown | No necropsy planned]

439 F 20 May 1990 243 410 SD-WAP 20 May 1990 690285
Birth

You created this PDF from an application that is not licensed to print to novaPDF printer (http://www.novapdf.com)

1 Jul 1993
Death
[Death by: Unknown means]

440 F 27 Oct 1990 42 251 SANDIEGOZ 27 Oct 1990 590451
Birth QUEEN
HOLIDAY 1 Nov 1991 ______
Loan to
MEMPHIS 22 Feb 1992 12685
Transfer
26 Sep 1999
Death
[Death by: Euthanasia (medical) + Incinerate + Generalized + Mechanical abnormality]

441 M 27 Oct 1990 192 402 SANDIEGOZ 27 Oct 1990 590452
Birth
29 Oct 1990
Death
[Death by: Unknown means]

442 M 4 Nov 1990 192 22 SANDIEGOZ 4 Nov 1990 590457
Birth
17 May 1991
Death
[Death by: Unknown means]

443 M 21 Nov 1990 243 416 SD-WAP 21 Nov 1990 690693
Birth
3 Feb 1991
Death
[Death by: Unknown means]

444 M 21 Nov 1990 243 416 SD-WAP 21 Nov 1990 690694
Birth KING
HOLIDAY 1 Nov 1991 ______
Loan to
MEMPHIS 22 Feb 1992 12684
Transfer
17 Sep 2001
Death
[Death by: Euthanasia (medical) + Incinerate + Generalized + Mechanical abnormality]

445 F 13 Jan 1991 243 410 SD-WAP 13 Jan 1991 691021
Birth
EVANSVLLE 26 May 1993 193043
Loan to
29 Jun 1995
Death
[Death by: Unknown means]

446 F 13 Jan 1991 243 410 SD-WAP 13 Jan 1991 691022
Birth
EVANSVLLE 26 May 1993 193044
Loan to
25 Jul 1996
Death
[Death by: Unknown means]

447 F 1 Mar 1991 415 422 ST LOUIS 1 Mar 1991 910306
Birth
4 Mar 1991
Death
[Death by: Unknown means]

448 F 2 Mar 1991 42 421 SANDIEGOZ 2 Mar 1991 591096
Birth
JOHNSONLA 11 Jan 1993 ______

You created this PDF from an application that is not licensed to print to novaPDF printer (http://www.novapdf.com)

Loan to
~ Oct 1994
Death
[Death by: Unknown means]

449 M 5 Mar 1991 42 252 SANDIEGOZ 5 Mar 1991 591105
Birth
4 Jun 1992
Death
[Death by: Unknown means]

450 F 5 Mar 1991 42 252 SANDIEGOZ 5 Mar 1991 591106
Birth
JOHNSONLA 11 Jan 1993 ______
Loan to
~ Feb 1996 AZA504
ltf

451 F 20 Mar 1991 42 194 SANDIEGOZ 20 Mar 1991 591138
Birth
19 Oct 2001
Death
[Death by: Unknown means]

452 M 19 Apr 1991 192 402 SANDIEGOZ 19 Apr 1991 591234
Birth
19 Apr 1991
Death
[Death by: Unknown means]

453 F 28 Apr 1991 192 251 SANDIEGOZ 28 Apr 1991 591243
Birth
HOLIDAY 1 Nov 1991 ______
Transfer
29 Jan 1992
Death
[Death by: Unknown means]

454 F 3 May 1991 243 439 SD-WAP 3 May 1991 691233
Birth
30 May 1991
Death
[Death by: Unknown means]

455 M 3 Jun 1991 243 416 SD-WAP 3 Jun 1991 691377
Birth
22 Feb 1994
Death
[Death by: Unknown means]

456 M 21 Jun 1991 243 432 SD-WAP 21 Jun 1991 691446
Birth
23 Jul 1993
Death
[Death by: Unknown means]

457 F 12 Aug 1991 243 410 SD-WAP 12 Aug 1991 691539
Birth
JOHNSONLA 11 Apr 1994 ______
Loan to
5 Oct 1995
Death
[Death by: Unknown means]

458 M 2 Sep 1991 206 244 PALM DES 2 Sep 1991 491025
Birth
28 Sep 1991
Death
[Death by: Unknown means]

You created this PDF from an application that is not licensed to print to novaPDF printer (http://www.novapdf.com)

459 F 21 Sep 1991 42 194 SANDIEGOZ 21 Sep 1991 591489
Birth
21 Sep 1991
Death
[Death by: Unknown means]

460 F 16 Oct 1991 206 412 PALM DES 16 Oct 1991 491029
Birth
18 Oct 1991
Death
[Death by: Unknown means]

461 F 23 Oct 1991 192 402 SANDIEGOZ 23 Oct 1991 591530
Birth
5 Aug 1999
Death
[Death by: Unknown means]

462 F 31 Oct 1991 427 429 CINCINNAT 31 Oct 1991 191242
Birth
WILDS 5 Jun 1992 920604
Loan to
21 Mar 1995
Death
[Death by: Unknown means]

463 F 5 Nov 1991 243 439 SD-WAP 5 Nov 1991 691697
Birth
EVANSVLLE 26 May 1993 193045
Loan to
21 Sep 1993
Death
[Death by: Unknown means]

464 F 17 Nov 1991 42 252 SANDIEGOZ 17 Nov 1991 591546
Birth PRINCESS
MEMPHIS 12 Jun 1992 13046
Transfer
19 Apr 2002
Death
[Death by: Unknown means]

465 F 10 Dec 1991 427 428 CINCINNAT 10 Dec 1991 191303
Birth SPREADHORN
WILDS 5 Jun 1992 920605
Loan to
29 Mar 2000
Death
[Death by: Euthanasia (medical) + Bury + Generalized +
Metabolism]

466 F 16 Dec 1991 243 416 SD-WAP 16 Dec 1991 691768
Birth
JOHNSONLA 11 Apr 1994 ______
ltf

467 M 16 Dec 1991 243 416 SD-WAP 16 Dec 1991 691769
Birth
EVANSVLLE 18 Feb 1994 194010
Loan to
SHADOW 8 Oct 1997 ______
ltf

468 F 10 Feb 1992 243 432 SD-WAP 10 Feb 1992 692056
Birth
4 Jun 1992
Death
[Death by: Unknown means]

You created this PDF from an application that is not licensed to print to novaPDF printer (http://www.novapdf.com)

469 F 17 Feb 1992 243 410 SD-WAP 17 Feb 1992 692063
Birth
24 Feb 1997
Death
[Death by: Unknown means]

470 F 17 Feb 1992 243 410 SD-WAP 17 Feb 1992 692064
Birth
JOHNSONLA 11 Apr 1994 ______
Loan to
~ Feb 1996 ______
ltf

471 F 1 Mar 1992 42 251 SANDIEGOZ 1 Mar 1992 592057
Birth
LANGLEY 2 Nov 1994 H94014
Transfer
17 Nov 1997
Death
[Death by: Unknown means]

472 F 1 Mar 1992 42 251 SANDIEGOZ 1 Mar 1992 592058
Birth
ST LOUIS 25 Aug 1992 920850
Loan to
28 Jan 1995
Death
[Death by: Unknown means]

473 M 10 Mar 1992 192 421 SANDIEGOZ 10 Mar 1992 592072
Birth
JOHNSONLA 11 Jan 1993 ______
Loan to
~ Jul 1996 ______
ltf

474 M 15 Mar 1992 42 430 SANDIEGOZ 15 Mar 1992 592089
Birth
3 Feb 1993
Death
[Death by: Unknown means]

475 F 15 Mar 1992 42 430 SANDIEGOZ 15 Mar 1992 592090
Birth
ST LOUIS 25 Aug 1992 920849
Transfer
7 Sep 1997
Death
[Death by: Unknown means]

476 F 26 Mar 1992 243 445 SD-WAP 26 Mar 1992 692118
Birth
26 Mar 1992
Death
[Death by: Unknown means]

477 F 28 Mar 1992 206 244 PALM DES 28 Mar 1992 492014
Birth
28 Mar 1992
Death
[Death by: Environ/Behav conditions ┼ Unknown ┼ Respiratory ┼ Unknown after necropsy]

478 M 6 Apr 1992 243 446 SD-WAP 6 Apr 1992 692141
Birth
8 Apr 1992
Death
[Death by: Unknown means]

You created this PDF from an application that is not licensed to print to novaPDF printer (http://www.novapdf.com)

```
        479   F    10 Apr 1992     415      422  ST LOUIS   10 Apr 1992 920444
Birth      MARIE
                                                 JOHNSONLA   4 Feb 1993 _______
ltf

        480   F    17 Apr 1992     UNK      402  SANDIEGOZ  17 Apr 1992 592157
Birth      CONNY
                                                 LANGLEY     2 Nov 1994 H94015
Transfer
                                                             2 Nov 1997
Death
                           [Death by: Unknown means]

        481   F     2 May 1992     243      439  SD-WAP      2 May 1992 692198
Birth
                                                 EVANSVLLE  26 May 1993 193046
Loan to
                                                            29 Mar 1995
Death
                           [Death by: Unknown means]

        482   M     2 May 1992     243      439  SD-WAP      2 May 1992 692199
Birth
                                                            20 Jun 1992
Death
                           [Death by: Unknown means]

        483   M     4 May 1992     192      194  SANDIEGOZ   4 May 1992 592177
Birth
                                                            30 Oct 1996
Death
                           [Death by: Unknown means]

        484   M    10 May 1992     427      428  CINCINNAT  10 May 1992 192087
Birth
                                                 WILDS       5 Jun 1992 920607
Loan to
                                                            17 May 1996
Death
                           [Death by: Unknown means]

        485   F    10 May 1992     427      428  CINCINNAT  10 May 1992 192088
Birth
                                                 WILDS       5 Jun 1992 920606
Loan to
                                                            29 May 1994
Death
                           [Death by: Unknown means]

        486   F    13 May 1992     192      252  SANDIEGOZ  13 May 1992 592198
Birth
                                                 JOHNSONLA  11 Jan 1993 _______
Loan to
                                                             ~ Jan 1993
Death
                           [Death by: Unknown means]

        487   F    13 May 1992     192      252  SANDIEGOZ  13 May 1992 592199
Birth
                                                            15 May 1992
Death
                           [Death by: Unknown means]

        488   M    26 May 1992     206      412  PALM DES   26 May 1992 492020
Birth
                                                            26 May 1992
Death
                  [Death by: Environ/Behav conditions + Unknown + Respiratory + Unknown
```


You created this PDF from an application that is not licensed to print to novaPDF printer (http://www.novapdf.com)

after necropsy]

489 F 26 May 1992 206 412 PALM DES 26 May 1992 492021
Birth
13 Sep 1992
Death
[Death by: Infection associated + Unknown + Respiratory + Unknown after necropsy]

490 F 24 Jun 1992 243 416 SD-WAP 24 Jun 1992 692393
Birth
27 Dec 1994
Death
[Death by: Unknown means]

491 M 24 Jun 1992 243 416 SD-WAP 24 Jun 1992 692394
Birth
25 Nov 1993
Death
[Death by: Unknown means]

492 M 5 Sep 1992 192 251 SANDIEGOZ 5 Sep 1992 592374
Birth
12 Oct 1992
Death
[Death by: Unknown means]

493 M 18 Sep 1992 243 445 SD-WAP 18 Sep 1992 692518
Birth
29 Jan 1993
Death
[Death by: Unknown means]

494 F 22 Oct 1992 243 445 SD-WAP 22 Oct 1992 692598
Birth
28 Oct 1992
Death
[Death by: Unknown means]

495 F 3 Nov 1992 243 439 SD-WAP 3 Nov 1992 692615
Birth
24 Apr 1997
Death
[Death by: Unknown means]

496 F 3 Nov 1992 243 439 SD-WAP 3 Nov 1992 692616
Birth
23 Nov 1992
Death
[Death by: Unknown means]

497 M 26 Nov 1992 206 244 PALM DES 26 Nov 1992 492040
Birth
SANDIEGOZ 26 May 1993 593070
Transfer
EVANSVLLE 27 May 1993 193042
Loan to
31 Oct 1993
Death
[Death by: Unknown means]

498 M 31 Mar 1994 272 321 ALMERIA 31 Mar 1994 EE498
Birth SN
10 Dec 1997
Death
[Death by: Unknown means]

499 M 31 Mar 1994 272 321 ALMERIA 31 Mar 1994 EE499
Birth SN

You created this PDF from an application that is not licensed to print to novaPDF printer (http://www.novapdf.com)

6 Feb 1997
Death
[Death by: Unknown means]

500 M 1 Apr 1994 114 341 ALMERIA 1 Apr 1994 EE500
Birth SN
30 Sep 1994
Death
[Death by: Unknown means]

501 F 1 Jul 1994 125 255 ALMERIA 1 Jul 1994 EE501
Birth CHERGA
BOUKORNIN 13 Dec 1999 UNK
Transfer
15 Dec 2000
Death
[Death by: Unknown means]

502 M 13 Jul 1994 199 159 ALMERIA 13 Jul 1994 EE502
Birth CLARA
8 Dec 2002
Death
[Death by: Unknown means]

503 F 13 Jul 1994 199 159 ALMERIA 13 Jul 1994 EE503
Birth RUBIA
BOUKORNIN 13 Dec 1999 EE503
Transfer
27 Mar 2003
Death
[Death by: Unknown means]

504 M 27 Aug 1994 272 167 ALMERIA 27 Aug 1994 EE504
Birth SN
28 Sep 1994
Death
[Death by: Unknown means]

505 F 27 Aug 1994 272 167 ALMERIA 27 Aug 1994 EE505
Birth SN
17 Apr 1995
Death
[Death by: Unknown means]

506 F 6 Sep 1994 199 110 ALMERIA 6 Sep 1994 EE506
Birth SONIA
15 Nov 2001
Death
[Death by: Unknown means]

507 F 6 Sep 1994 199 110 ALMERIA 6 Sep 1994 EE507
Birth TERE
25 Nov 1995
Death
[Death by: Unknown means]

508 F 15 Sep 1994 125 270 ALMERIA 15 Sep 1994 EE508
Birth SIDINA
3 Oct 2002
Death
[Death by: Unknown means]

509 M 8 Dec 1994 365 261 ALMERIA 8 Dec 1994 EE509
Birth SN
27 Apr 1997
Death
[Death by: Unknown means]

510 M 8 Dec 1994 365 261 ALMERIA 8 Dec 1994 EE510

You created this PDF from an application that is not licensed to print to novaPDF printer (http://www.novapdf.com)

Birth SN
11 Oct 1996
Death
[Death by: Unknown means]

511 F 13 Dec 1994 125 341 ALMERIA 13 Dec 1994 EE511
Birth SN
10 Jul 1997
Death
[Death by: Unknown means]

512 M 21 Dec 1994 365 335 ALMERIA 21 Dec 1994 EE512
Birth SN
14 Aug 1996
Death
[Death by: Unknown means]

513 M 21 Feb 1994 264 356 MADRID Z 21 Feb 1994 MA513
Birth ABDUL
22 Feb 1994
Death
[Death by: Unknown means]

514 F 3 Jun 1994 264 354 MADRID Z 3 Jun 1994 MA514
Birth CLEOPATRA
24 Aug 1994
Death
[Death by: Unknown means]

515 M 4 Oct 1994 264 356 MADRID Z 4 Oct 1994 MA515
Birth MAHDI
3 Nov 1997
Death
[Death by: Unknown means]

516 M 7 Mar 1995 125 255 ALMERIA 7 Mar 1995 EE516
Birth
13 Apr 2004
Death
[Death by: Unknown means]

517 M 19 Mar 1995 365 331 ALMERIA 19 Mar 1995 EE517
Birth
21 Mar 1995
Death
[Death by: Unknown means]

518 F 19 Mar 1995 365 331 ALMERIA 19 Mar 1995 EE518
Birth
31 Aug 1997
Death
[Death by: Unknown means]

519 F 22 May 1995 125 397 ALMERIA 22 May 1995 EE519
Birth
19 Apr 2002
Death
[Death by: Unknown means]

520 M 15 Jul 1995 365 335 ALMERIA 15 Jul 1995 EE520
Birth RETBET
18 Jul 1995
Death
[Death by: Unknown means]

521 M 15 Jul 1995 365 335 ALMERIA 15 Jul 1995 EE521
Birth JEDID
BOUKORNIN 13 Dec 1999 UNK
Transfer

You created this PDF from an application that is not licensed to print to novaPDF printer (http://www.novapdf.com)

16 Jan 2001
Death
[Death by: Unknown means]

522 M 17 Sep 1995 374 376 ALMERIA 17 Sep 1995 EE522
Birth
21 Sep 1995
Death
[Death by: Unknown means]

523 M 21 Sep 1995 374 371 ALMERIA 21 Sep 1995 EE523
Birth MONASTIR
29 Dec 1999
Death
[Death by: Unknown means]

524 M 30 Oct 1995 125 255 ALMERIA 30 Oct 1995 EE524
Birth HERNAN
5 May 2005
Death
[Death by: Unknown means]

525 F 30 Oct 1995 125 255 ALMERIA 30 Oct 1995 EE525
Birth
9 May 2004
Death
[Death by: Unknown means]

526 M 25 Nov 1995 365 331 ALMERIA 25 Nov 1995 EE526
Birth
16 Nov 1996
Death
[Death by: Unknown means]

527 M 10 Mar 1996 365 335 ALMERIA 10 Mar 1996 EE527
Birth BARDO
BOUKORNIN 13 Dec 1999 UNK
Transfer
5 Apr 2000
Death
[Death by: Unknown means]

528 M 10 Mar 1996 365 335 ALMERIA 10 Mar 1996 EE528
Birth DRIGO
19 Oct 1997
Death
[Death by: Unknown means]

529 M 14 Apr 1995 264 356 MADRID Z 14 Apr 1995 MA529
Birth BEN-JALIL
ALMERIA 3 Dec 1997 EE529
Transfer
27 Jun 2000
Death
[Death by: Unknown means]

530 M 14 May 1995 264 354 MADRID Z 14 May 1995 MA530
Birth YAZID
29 Jun 1995
Death
[Death by: Unknown means]

531 M 15 Nov 1993 198 288 ALMERIA 15 Nov 1993 CASI21
Birth
2 Mar 1999
Death
[Death by: Unknown means]

532 F 15 Sep 1996 531 258 ALMERIA 15 Sep 1996 CASI22

You created this PDF from an application that is not licensed to print to novaPDF printer (http://www.novapdf.com)

Birth
31 Mar 2000
Death
[Death by: Unknown means]

533 F 14 May 1997 290 380 ALMERIA 14 May 1997 EE533
Birth
22 Sep 2010
Death
[Death by: Old age + Incinerate + Generalized +
Unknown after necropsy]

534 M 15 Apr 1997 290 364 ALMERIA 15 Apr 1997 EE534
Birth
11 Oct 2002
Death
[Death by: Unknown means]

535 F 16 May 1997 290 322 ALMERIA 16 May 1997 EE535
Birth
20 May 1997
Death
[Death by: Unknown means]

536 F 16 May 1997 290 322 ALMERIA 16 May 1997 EE536
Birth
17 May 1997
Death
[Death by: Unknown means]

537 M 25 May 1997 154 511 ALMERIA 25 May 1997 EE537
Birth
22 Dec 2001
Death
[Death by: Unknown means]

538 F 25 May 1997 154 511 ALMERIA 25 May 1997 EE538
Birth
17 Aug 2003
Death
[Death by: Unknown means]

539 F 16 Sep 1997 346 273 ALMERIA 16 Sep 1997 EE539
Birth
18 Apr 2004
Death
[Death by: Unknown means]

540 M 18 Sep 1997 346 377 ALMERIA 18 Sep 1997 EE540
Birth
5 Sep 2006
Death
[Death by: Unknown means]

541 M 20 Sep 1997 346 371 ALMERIA 20 Sep 1997 EE541
Birth
4 Oct 2002
Death
[Death by: Unknown means]

542 M 21 Sep 1997 154 329 ALMERIA 21 Sep 1997 EE542
Birth
15 Oct 1997
Death
[Death by: Unknown means]

543 M 21 Sep 1997 154 329 ALMERIA 21 Sep 1997 EE543
Birth
26 Nov 1997

You created this PDF from an application that is not licensed to print to novaPDF printer (http://www.novapdf.com)

Death
[Death by: Unknown means]

544 M 30 Sep 1997 346 376 ALMERIA 30 Sep 1997 EE544
Birth
2 Oct 1997
Death
[Death by: Unknown means]

545 M 6 Oct 1997 346 361 ALMERIA 6 Oct 1997 EE545
Birth
7 Oct 1997
Death
[Death by: Unknown means]

546 M 9 Oct 1997 290 363 ALMERIA 9 Oct 1997 EE546
Birth
24 Sep 2006
Death
[Death by: Unknown means]

547 F 14 Oct 1997 290 323 ALMERIA 14 Oct 1997 EE547
Birth
3 Jun 2003
Death
[Death by: Unknown means]

548 M 30 Oct 1997 290 391 ALMERIA 30 Oct 1997 EE548
Birth
2 Nov 1997
Death
[Death by: Unknown means]

549 M 13 Nov 1997 290 378 ALMERIA 13 Nov 1997 EE549
Birth

550 M 18 Nov 1997 290 359 ALMERIA 18 Nov 1997 EE550
Birth
20 Nov 1997
Death
[Death by: Unknown means]

551 F 18 Nov 1997 290 359 ALMERIA 18 Nov 1997 EE551
Birth
25 Nov 1997
Death
[Death by: Unknown means]

552 M 30 Nov 1997 290 322 ALMERIA 30 Nov 1997 EE552
Birth
10 Dec 1997
Death
[Death by: Unknown means]

553 F 2 Dec 1997 290 390 ALMERIA 2 Dec 1997 EE553
Birth
1 May 2010
Death
[Death by: Unknown means]

554 M 12 Dec 1997 290 228 ALMERIA 12 Dec 1997 EE554
Birth
16 Dec 1997
Death
[Death by: Unknown means]

555 F 16 Mar 1998 346 351 ALMERIA 16 Mar 1998 EE555
Birth
10 Jul 2006

You created this PDF from an application that is not licensed to print to novaPDF printer (http://www.novapdf.com)

Death
[Death by: Self-inflicted injuries ┼ Incinerate ┼ Generalized ┼ Trauma]

556 M 30 Mar 1998 346 371 ALMERIA 30 Mar 1998 EE556
Birth KSOUR
19 Dec 2002
Death
[Death by: Unknown means]

557 F 10 Apr 1998 346 376 ALMERIA 10 Apr 1998 EE557
Birth
9 Jul 1998
Death
[Death by: Unknown means]

558 M 22 Apr 1998 336 385 ALMERIA 22 Apr 1998 EE558
Birth PACOGO
BOUKORNIN 13 Dec 1999 UNK
Transfer
26 Dec 2000
Death
[Death by: Unknown means]

559 M 27 Apr 1998 370 335 ALMERIA 27 Apr 1998 EE559
Birth EMILIO
BOUKORNIN 13 Dec 1999 UNK
Transfer
25 Jul 2000
Death
[Death by: Unknown means]

560 F 27 Apr 1998 370 335 ALMERIA 27 Apr 1998 EE560
Birth LORA
2 Aug 2004
Death
[Death by: Unknown means]

561 M 27 Apr 1998 370 506 ALMERIA 27 Apr 1998 EE561
Birth TAMAYO
18 Feb 2008
Death
[Death by: Old age ┼ Incinerate ┼ Urinary ┼ Toxicity]

562 F 27 Apr 1998 370 506 ALMERIA 27 Apr 1998 EE562
Birth
21 May 1998
Death
[Death by: Unknown means]

563 F 28 Apr 1998 370 387 ALMERIA 28 Apr 1998 EE563
Birth HAMMA
BOUKORNIN 13 Dec 1999 UNK
Transfer
6 May 2000
Death
[Death by: Unknown means]

564 F 28 Apr 1998 370 387 ALMERIA 28 Apr 1998 EE564
Birth HADDA
14 May 1998
Death
[Death by: Unknown means]

565 M 19 Jun 1998 370 503 ALMERIA 19 Jun 1998 EE565
Birth KALAAT
3 Oct 2006
Death
[Death by: Unknown means]

You created this PDF from an application that is not licensed to print to novaPDF printer (http://www.novapdf.com)

566 F 19 Jun 1998 370 503 ALMERIA 19 Jun 1998 EE566
Birth SENAN
22 Sep 2006
Death
[Death by: Injury from predator | Incinerate | No necropsy planned]

567 M 22 Jun 1998 370 159 ALMERIA 22 Jun 1998 EE567
Birth
3 Jul 1998
Death
[Death by: Unknown means]

568 F 22 Jul 1998 256 361 ALMERIA 22 Jul 1998 EE568
Birth
11 Aug 1998
Death
[Death by: Unknown means]

569 M 28 Jul 1998 158 349 ALMERIA 28 Jul 1998 EE569
Birth
3 Feb 2006
Death
[Death by: Unknown means]

570 F 12 Aug 1998 502 347 ALMERIA 12 Aug 1998 EE570
Birth
24 Oct 2012
Death
[Death by: Unknown means]

571 F 25 Aug 1998 256 322 ALMERIA 25 Aug 1998 EE571
Birth LEDESMA
19 Oct 2010
Death
[Death by: Unknown means]

572 F 31 Aug 1998 256 323 ALMERIA 31 Aug 1998 EE572
Birth TRIANA
18 Jul 2012
Death
[Death by: Unknown means]

573 F 6 Sep 1998 256 228 ALMERIA 6 Sep 1998 EE573
Birth MUNA
BOUKORNIN 13 Dec 1999 UNK
Transfer
25 Sep 2000
Death
[Death by: Unknown means]

574 F 6 Sep 1998 256 228 ALMERIA 6 Sep 1998 EE574
Birth MOIRA
BOUKORNIN 13 Dec 1999 UNK
Transfer
9 Oct 2000
Death
[Death by: Unknown means]

575 F 16 Jan 1999 502 385 ALMERIA 16 Jan 1999 EE575
Birth
17 Mar 1999
Death
[Death by: Unknown means]

576 F 17 Mar 1999 256 322 ALMERIA 17 Mar 1999 EE576
Birth
22 Apr 2004

You created this PDF from an application that is not licensed to print to novaPDF printer (http://www.novapdf.com)

Death								
[Death by: Unknown means]								
Birth	577	F	31 Mar 1999	256	323	ALMERIA	31 Mar 1999	EE577
Birth	578	F	22 Jun 1999	502	347	ALMERIA	22 Jun 1999	EE578
Death							12 Oct 2012	
[Death by: Unknown means]								
Birth	579	M	17 Jul 1999	502	385	ALMERIA	17 Jul 1999	EE579
Death							24 Sep 2001	
[Death by: Unknown means]								
Birth	580	M	17 Jul 1999	502	385	ALMERIA	17 Jul 1999	EE580
Transfer						TABERNAS	8 Nov 2007	0353
Death							25 Aug 2010	
[Death by: Unknown means]								
Birth	581	F	23 Aug 1999	502	321	ALMERIA	23 Aug 1999	EE581
Death							2 Mar 2008	
[Death by: Unknown means]								
Birth	582	M	23 Feb 2000	502	347	ALMERIA	23 Feb 2000	EE582
Birth	583	M	28 Feb 2000	502	385	ALMERIA	28 Feb 2000	EE583
Transfer						TABERNAS	8 Nov 2007	UNK
Death							3 Nov 2010	
[Death by: Unknown means]								
Birth	584	M	28 Feb 2000	502	385	ALMERIA	28 Feb 2000	EE584
Transfer						TABERNAS	8 Nov 2007	0352
Birth	585	M	26 Mar 2000	502	321	ALMERIA	26 Mar 2000	EE585
Death							6 Dec 2003	
[Death by: Unknown means]								
Birth	586	F	26 Mar 2000	502	321	ALMERIA	26 Mar 2000	EE586
Death							1 Jan 2001	
[Death by: Unknown means]								
Birth	587	M	7 Apr 2000	546	555	ALMERIA	7 Apr 2000	EE587
Death							2 Jan 2002	
[Death by: Unknown means]								
Birth	588	F	7 Apr 2000	546	555	ALMERIA	7 Apr 2000	EE588

You created this PDF from an application that is not licensed to print to novaPDF printer (http://www.novapdf.com)

589 F 23 Jun 2000 546 361 ALMERIA 23 Jun 2000 EE589
Birth
29 Jun 2000
Death
[Death by: Unknown means]

590 M 10 Jul 2000 370 547 ALMERIA 10 Jul 2000 EE590
Birth
TABERNAS 10 Jul 2001 EE590
Transfer
24 Mar 2004
Death
[Death by: Unknown means]

591 F 15 Jul 2000 370 364 ALMERIA 15 Jul 2000 EE591
Birth

592 F 18 Jul 2000 370 380 ALMERIA 18 Jul 2000 EE592
Birth
21 Oct 2007
Death
[Death by: Euthanasia (medical) + Incinerate + Digestive + Unknown after necropsy]

593 M 20 Jul 2000 534 508 ALMERIA 20 Jul 2000 EE593
Birth
12 Mar 2004
Death
[Death by: Unknown means]

594 M 20 Jul 2000 534 508 ALMERIA 20 Jul 2000 EE594
Birth
TABERNAS 8 Nov 2007 0351
Transfer
19 Nov 2007
Death
[Death by: Infection associated + Incinerate + Generalized + Unknown after necropsy]

595 M 22 Jul 2000 534 538 ALMERIA 22 Jul 2000 EE595
Birth
22 Sep 2003
Death
[Death by: Unknown means]

596 F 22 Jul 2000 534 538 ALMERIA 22 Jul 2000 EE596
Birth
6 Apr 2004
Death
[Death by: Unknown means]

597 M 22 Jul 2000 370 553 ALMERIA 22 Jul 2000 EE597
Birth LENTISCO
TABERNAS 22 Jul 2001 EE597
Transfer
13 Sep 2003
Death
[Death by: Unknown means]

598 M 25 Jul 2000 370 533 ALMERIA 25 Jul 2000 EE598
Birth TOMILLO
TABERNAS 25 Jul 2001 EE598
Transfer
ALMERIA 8 Nov 2007 EE598
Transfer

599 M 25 Jul 2000 370 533 ALMERIA 25 Jul 2000 EE599
Birth ROMERO
TABERNAS 25 Jul 2001 EE599

You created this PDF from an application that is not licensed to print to novaPDF printer (http://www.novapdf.com)

```
Transfer
                                                           25 Mar 2004
Death
                            [Death by: Unknown means]

       600   M    30 Jul 2000     534     525  ALMERIA     30 Jul 2000  EE600
Birth
                                                           25 Oct 2003
Death
                            [Death by: Unknown means]

       601   F    31 Jul 2000     534     397  ALMERIA     31 Jul 2000  EE601
Birth     OREJA
                                                           24 Sep 2003
Death
                            [Death by: Unknown means]

       602   M    27 Mar 2000     546     376  BOUKORNIN   27 Mar 2000  BO602
Birth     GERARDO

       603   M    24 May 2000     398     391  BOUKORNIN   24 May 2000     BO
Birth     RAMON
                                                           11 Oct 2002
Death
                            [Death by: Unknown means]

       604   F    26 May 2000     398     359  BOUKORNIN   26 May 2000  BO604
Birth     TERESA
                                                            9 Jun 2000
Death
                            [Death by: Unknown means]

       605   F    27 May 2000     398     573  BOUKORNIN   27 May 2000  BO605
Birth     MAR
                                                            8 Jun 2000
Death
                            [Death by: Unknown means]

       606   F     8 Jun 2000     398     503  BOUKORNIN    8 Jun 2000  BO606
Birth
                                                            8 Jun 2000
Death
                            [Death by: Unknown means]

       607   M     4 Sep 2000     546     539  ALMERIA      4 Sep 2000  EE607
Birth     PALMITO
                                               TABERNAS     4 Sep 2001  EE607
Transfer
                                                            9 Jan 2004
Death
                            [Death by: Unknown means]

       608   F     8 Jan 2001     546     361  ALMERIA      8 Jan 2001  EE608
Birth

       609   F     8 Jan 2001     546     361  ALMERIA      8 Jan 2001  EE609
Birth
                                                           21 Jun 2012
Death
                            [Death by: Unknown means]

       610   M    23 Mar 2001     546     539  ALMERIA     23 Mar 2001  EE610
Birth
                                                            3 Mar 2011
Death
                            [Death by: Unknown means]

       611   M    23 Mar 2001     546     539  ALMERIA     23 Mar 2001  EE611
Birth
```


You created this PDF from an application that is not licensed to print to novaPDF printer (http://www.novapdf.com)

30 Mar 2010
Death
[Death by: Unknown means]

612 M 31 Mar 2001 546 555 ALMERIA 31 Mar 2001 EE612
Birth
15 Sep 2006
Death
[Death by: Unknown means]

613 M 31 Mar 2001 546 555 ALMERIA 31 Mar 2001 EE613
Birth
4 Nov 2006
Death
[Death by: Injury from predator | Incinerate | No necropsy planned]

614 F 14 Mar 2001 558 503 BOUKORNIN 14 Mar 2001 BO614
Birth EZZAHRA

615 M 23 Mar 2001 558 391 BOUKORNIN 23 Mar 2001 BO615
Birth LYS
4 May 2003
Death
[Death by: Unknown means]

616 F 24 Mar 2001 558 363 BOUKORNIN 24 Mar 2001 TU616
Birth MONTASSER

617 F 15 Apr 2001 546 588 ALMERIA 15 Apr 2001 EE617
Birth
24 Nov 2002
Death
[Death by: Unknown means]

618 M 17 Sep 2001 534 566 ALMERIA 17 Sep 2001 EE618
Birth
25 Oct 2005
Death
[Death by: Unknown means]

619 F 28 Sep 2001 524 506 ALMERIA 28 Sep 2001 EE619
Birth
28 Sep 2001
Death
[Death by: Unknown means]

620 F 29 Sep 2001 524 578 ALMERIA 29 Sep 2001 EE620
Birth
27 Aug 2009
Death
[Death by: Unknown means]

621 M 18 Jan 2002 534 560 ALMERIA 18 Jan 2002 EE621
Birth
18 Jan 2002
Death
[Death by: Unknown means]

622 M 18 Jan 2002 534 560 ALMERIA 18 Jan 2002 EE622
Birth
LUGO 20 Feb 2013 UNK
Transfer

623 F 2 Apr 2002 534 566 ALMERIA 2 Apr 2002 EE623
Birth
27 Jun 2004
Death
[Death by: Unknown means]

You created this PDF from an application that is not licensed to print to novaPDF printer (http://www.novapdf.com)

624 M 29 Jul 2002 534 560 ALMERIA 29 Jul 2002 EE624
Birth
11 May 2009
Death
[Death by: Unknown means]

625 F 9 Apr 2002 602 503 BOUKORNIN 9 Apr 2002 BO625
Birth

626 M 13 Apr 2002 602 391 BOUKORNIN 13 Apr 2002 BO626
Birth

627 M 14 Apr 2002 602 363 BOUKORNIN 14 Apr 2002 BO627
Birth
26 Jun 2002
Death
[Death by: Unknown means]

628 M 12 Sep 2002 582 508 ALMERIA 12 Sep 2002 EE628
Birth
20 Sep 2002
Death
[Death by: Unknown means]

629 M 22 Sep 2002 516 533 ALMERIA 22 Sep 2002 EE629
Birth
20 Apr 2005
Death
[Death by: Injury from predator | Incinerate | Necropsy planned later | Toxicity]

630 M 26 Sep 2002 516 578 ALMERIA 26 Sep 2002 EE630
Birth
2 Oct 2002
Death
[Death by: Unknown means]

631 M 26 Sep 2002 516 578 ALMERIA 26 Sep 2002 EE631
Birth
28 Sep 2002
Death
[Death by: Unknown means]

632 F 26 Sep 2002 565 385 ALMERIA 26 Sep 2002 EE632
Birth
10 Nov 2008
Death
[Death by: Unknown means]

633 F 12 Oct 2002 565 347 ALMERIA 12 Oct 2002 EE633
Birth
24 Apr 2007
Death
[Death by: Unknown means]

634 M 2 Dec 2002 534 566 ALMERIA 2 Dec 2002 EE634
Birth
14 Dec 2002
Death
[Death by: Unknown means]

635 M 4 Jan 2003 565 321 ALMERIA 4 Jan 2003 EE635
Birth TORETE
12 Sep 2005
Death
[Death by: Unknown means]

636 F 4 Mar 2003 534 560 ALMERIA 4 Mar 2003 EE636

You created this PDF from an application that is not licensed to print to novaPDF printer (http://www.novapdf.com)

Birth MARIA JOSE
13 Sep 2004
Death
[Death by: Unknown means]

637 F 30 Mar 2003 565 385 ALMERIA 30 Mar 2003 EE637
Birth
4 Apr 2003
Death
[Death by: Unknown means]

638 M 30 Mar 2003 565 385 ALMERIA 30 Mar 2003 EE638
Birth
28 Aug 2007
Death
[Death by: Unknown means]

639 F 12 Apr 2003 565 347 ALMERIA 12 Apr 2003 EE639
Birth
18 Apr 2003
Death
[Death by: Unknown means]

640 F 1 May 2003 524 596 ALMERIA 1 May 2003 EE640
Birth
26 Dec 2011
Death
[Death by: Unknown means]

641 F 1 May 2003 524 596 ALMERIA 1 May 2003 EE641
Birth
19 Jan 2013
Death
[Death by: Unknown means]

642 M 4 May 2003 584 364 ALMERIA 4 May 2003 EE642
Birth
ESTEPONA 13 Jul 2010 UNK
Transfer

643 M 10 May 2003 594 609 ALMERIA 10 May 2003 EE643
Birth
6 Aug 2011
Death
[Death by: Unknown means]

644 M 10 May 2003 594 609 ALMERIA 10 May 2003 EE644
Birth
13 May 2003
Death
[Death by: Unknown means]

645 M 12 May 2003 584 592 ALMERIA 12 May 2003 EE645
Birth
ESTEPONA 13 Jul 2010 UNK
Transfer

646 M 12 May 2003 584 592 ALMERIA 12 May 2003 EE646
Birth
ESTEPONA 13 Jul 2010 UNK
Transfer

647 F 15 May 2003 594 588 ALMERIA 15 May 2003 EE647
Birth
LA LAJITA 27 Nov 2006 LA647
Transfer

648 M 17 May 2003 584 591 ALMERIA 17 May 2003 EE648
Birth

You created this PDF from an application that is not licensed to print to novaPDF printer (http://www.novapdf.com)

```
                                              ESTEPONA   13 Jul 2010    UNK
Transfer
       649    M    17 May 2003     584     591  ALMERIA    17 May 2003  EE649
Birth
                                              ESTEPONA   13 Jul 2010    UNK
Transfer
       650    M    19 May 2003     561     576  ALMERIA    19 May 2003  EE650
Birth
                                              LA LAJITA  27 Nov 2006  LA650
Transfer
       651    M     5 Jul 1992     427     429  WILDS       5 Jul 1992 920701
Birth
                                                          6 Jun 1995
Death
                           [Death by: Unknown means]
       652    F     5 Jul 1992     427     429  WILDS       5 Jul 1992 920702
Birth
                                                         14 Feb 1996
Death
                           [Death by: Unknown means]
       653    F    30 Nov 1992     427     428  WILDS      30 Nov 1992 921101
Birth
                                                         26 Sep 1997
Death
                           [Death by: Unknown means]
       654    ?    24 Jan 1993     444     464  MEMPHIS    24 Jan 1993  H0617
Birth
                                                         24 Jan 1993
Death
                           [Death by: Unknown means]
       655    M    16 Feb 1993     243     416  SD-WAP     16 Feb 1993 693066
Birth
                                                         26 Oct 1993
Death
                           [Death by: Unknown means]
       656    M    16 Feb 1993     243     416  SD-WAP     16 Feb 1993 693067
Birth
                                              OR WILDLF   7 Dec 1993  93252
Loan to
                                                          5 May 2000
Death
                           [Death by: Injury from exhibit mate + Bury + No
necropsy planned]
       657    F    20 Feb 1993     192     194  SANDIEGOZ  20 Feb 1993 593042
Birth
                                                         20 Feb 1993
Death
                           [Death by: Unknown means]
       658    F    20 Feb 1993     192     430  SANDIEGOZ  20 Feb 1993 593043
Birth
                                                         20 Feb 1993
Death
                           [Death by: Unknown means]
       659    M    20 Feb 1993     192     430  SANDIEGOZ  20 Feb 1993 593044
Birth
                                              JOHNSONLA   1 Dec 1993 ______
Loan to
                                                          ~ Jan 1994
```


You created this PDF from an application that is not licensed to print to novaPDF printer (http://www.novapdf.com)

Death
[Death by: Unknown means]

660 F 21 Feb 1993 243 432 SD-WAP 21 Feb 1993 693073
Birth
JOHNSONLA 21 Aug 1998 ______
ltf

661 M 21 Feb 1993 243 432 SD-WAP 21 Feb 1993 693074
Birth
OR WILDLF 7 Dec 1993 93253
Loan to
30 Mar 1997
Death
[Death by: Unknown means]

662 M 10 Mar 1993 436 402 SANDIEGOZ 10 Mar 1993 593060
Birth
27 Aug 1997
Death
[Death by: Unknown means]

663 F 13 Mar 1993 192 252 SANDIEGOZ 13 Mar 1993 593066
Birth CORINNE
LANGLEY 2 Nov 1994 H94016
Transfer
UARAB EM 24 Apr 2005 UNK
ltf

664 ? 14 Mar 1993 436 421 SANDIEGOZ 14 Mar 1993 593067
Birth
14 Mar 1993
Death
[Death by: Unknown means]

665 M 17 Mar 1993 243 445 SD-WAP 17 Mar 1993 693100
Birth
JOHNSONLA 21 Aug 1998 ______
ltf

666 M 19 Mar 1993 206 412 PALM DES 19 Mar 1993 493017
Birth DUNCAN
SANDIEGOZ 22 Nov 1993 593353
Transfer
OR WILDLF 7 Dec 1993 93250
Loan to
4 May 1996
Death
[Death by: Unknown means]

667 M 19 Mar 1993 206 412 PALM DES 19 Mar 1993 493018
Birth
SANDIEGOZ 22 Nov 1993 593354
Transfer
OR WILDLF 7 Dec 1993 93251
Loan to
8 Mar 1999
Death
[Death by: Injury from exhibit mate ┼ Bury ┼ No
necropsy planned]

668 M 21 Mar 1993 243 457 SD-WAP 21 Mar 1993 693108
Birth
19 Feb 1995
Death
[Death by: Unknown means]

669 M 21 Mar 1993 243 457 SD-WAP 21 Mar 1993 693109
Birth

You created this PDF from an application that is not licensed to print to novaPDF printer (http://www.novapdf.com)

14 May 1993
Death
[Death by: Unknown means]

670 M 30 Mar 1993 243 466 SD-WAP 30 Mar 1993 693135
Birth
3 Apr 1993
Death
[Death by: Unknown means]

671 F 12 Apr 1993 444 440 MEMPHIS 12 Apr 1993 13601
Birth BELLE
5 Feb 1995
Death
[Death by: Unknown means]

672 F 12 Apr 1993 444 440 MEMPHIS 12 Apr 1993 13602
Birth ESTELLE
2 Apr 2005
Death
[Death by: Injury from predator | Incinerate |
Generalized | Trauma]

673 M 15 Apr 1993 243 446 SD-WAP 15 Apr 1993 693165
Birth
15 Apr 1993
Death
[Death by: Unknown means]

674 F 15 Apr 1993 243 446 SD-WAP 15 Apr 1993 693166
Birth
JOHNSONLA 11 Apr 1994 ______
ltf

675 F 20 Apr 1993 427 429 WILDS 20 Apr 1993 930401
Birth
27 Jul 1993
Death
[Death by: Injury from predator | Bury | No necropsy
planned]

676 M 20 Apr 1993 427 429 WILDS 20 Apr 1993 930402
Birth
23 Apr 1993
Death
[Death by: Unknown means]

677 M 29 Apr 1993 415 422 ST LOUIS 29 Apr 1993 930460
Birth MAC
JOHNSONLA 15 Nov 1993 ______
Loan to
6 Feb 1997
Death
[Death by: Unknown means]

678 M 29 Apr 1993 415 422 ST LOUIS 29 Apr 1993 930461
Birth RINGO
OR WILDLF 2 Jun 1994 93286
Loan to
18 Jul 1996
Death
[Death by: Unknown means]

679 M 3 Jun 1993 192 251 SANDIEGOZ 3 Jun 1993 593235
Birth
LANGLEY 2 Nov 1994 H94017
Transfer
10 Aug 1996
Death

You created this PDF from an application that is not licensed to print to novaPDF printer (http://www.novapdf.com)

[Death by: Unknown means]

680 M 8 Jun 1993 436 451 SANDIEGOZ 8 Jun 1993 593254
Birth COOPER
LANGLEY 2 Nov 1994 H94018
Transfer
ALDERGROV 3 Sep 1998 _______
Transfer
~ 1999
Death
[Death by: Unknown means]

681 M 11 Jun 1993 427 428 WILDS 11 Jun 1993 930601
Birth
5 Oct 1995
Death
[Death by: Unknown means]

682 M 29 Jun 1993 243 439 SD-WAP 29 Jun 1993 693427
Birth LARRY
WILDS 10 Aug 1995 950801
Loan to
10 Dec 1999
Death
[Death by: Unknown means]

683 M 20 Jul 1993 192 194 SANDIEGOZ 20 Jul 1993 593291
Birth
20 Jul 1993
Death
[Death by: Unknown means]

684 M 1 Sep 1993 206 412 PALM DES 1 Sep 1993 493032
Birth DRAKE
26 Jan 1994 493032
Transfer
OR WILDLF 29 Mar 1994 93278
Transfer
22 Sep 1996
Death
[Death by: Unknown means]

685 M 14 Sep 1993 444 464 MEMPHIS 14 Sep 1993 14002
Birth PRINCE
25 Mar 1994
Death
[Death by: Unknown means]

686 F 16 Sep 1993 243 432 SD-WAP 16 Sep 1993 693561
Birth
JOHNSONLA 21 Aug 1998 _______
ltf

687 F 16 Sep 1993 243 432 SD-WAP 16 Sep 1993 693562
Birth
JOHNSONLA 21 Aug 1998 _______
ltf

688 M 24 Sep 1993 243 457 SD-WAP 24 Sep 1993 5132
Birth
28 Oct 1993
Death
[Death by: Unknown means]

689 F 19 Oct 1993 192 430 SANDIEGOZ 19 Oct 1993 593389
Birth
8 Jul 2001
Death
[Death by: Unknown means]

You created this PDF from an application that is not licensed to print to novaPDF printer (http://www.novapdf.com)

690 F 24 Oct 1993 436 421 SANDIEGOZ 24 Oct 1993 593395
Birth
PALM DES 15 Aug 1995 495029
Loan to
OR WILDLF 5 Dec 1996 93430
Loan to
14 Mar 2004
Death
[Death by: Unknown means]

691 M 14 Nov 1993 415 472 ST LOUIS 14 Nov 1993 931105
Birth CLAUDE
OR WILDLF 2 Jun 1994 93287
Loan to
11 May 1999
Death
[Death by: Injury from exhibit mate + Unknown + No
necropsy planned]

692 F 14 Nov 1993 415 472 ST LOUIS 14 Nov 1993 931106
Birth CLAUDIA
OR WILDLF 2 Jun 1994 93288
Transfer
13 Sep 1994
Death
[Death by: Unknown means]

693 F 1 Dec 1993 243 416 SD-WAP 1 Dec 1993 693677
Birth
24 Apr 1997
Death
[Death by: Unknown means]

694 F 21 Jan 1994 243 466 SD-WAP 21 Jan 1994 694038
Birth
27 Jan 1994
Death
[Death by: Unknown means]

695 M 21 Jan 1994 243 466 SD-WAP 21 Jan 1994 694037
Birth
19 Jun 1995
Death
[Death by: Unknown means]

696 M 1 Feb 1994 192 251 SANDIEGOZ 1 Feb 1994 594039
Birth
1 Feb 1994
Death
[Death by: Euthanasia (medical) + Unknown + No
necropsy planned]

697 M 1 Feb 1994 192 251 SANDIEGOZ 1 Feb 1994 594040
Birth
1 Feb 1994
Death
[Death by: Unknown means]

698 M 2 Feb 1994 436 402 SANDIEGOZ 2 Feb 1994 594042
Birth
6 Jun 1994
Death
[Death by: Unknown means]

699 M 2 Feb 1994 436 402 SANDIEGOZ 2 Feb 1994 594043
Birth
4 Jun 2002
Death

You created this PDF from an application that is not licensed to print to novaPDF printer (http://www.novapdf.com)

[Death by: Unknown means]

700 M 12 Feb 1994 192 252 SANDIEGOZ 12 Feb 1994 594045
Birth
PEACE RV 19 Nov 2004 UNK
Transfer

701 M 25 Feb 1994 243 490 SD-WAP 25 Feb 1994 694082
Birth
1 Apr 1994
Death
[Death by: Unknown means]

702 M 9 Mar 1994 243 470 SD-WAP 9 Mar 1994 694098
Birth
8 Aug 1997
Death
[Death by: Unknown means]

703 M 10 Mar 1994 444 440 MEMPHIS 10 Mar 1994 14286
Birth DUKE
JOHNSONLA 20 Nov 1998 ______
ltf

704 F 10 Mar 1994 444 440 MEMPHIS 10 Mar 1994 14287
Birth DUTCHESS

705 M 11 Mar 1994 206 244 PALM DES 11 Mar 1994 494007
Birth
13 Mar 1994
Death
[Death by: Unknown means]

706 F 20 Mar 1994 483 451 SANDIEGOZ 20 Mar 1994 594098
Birth
27 May 2007
Death
[Death by: Unknown means]

707 F 20 Mar 1994 483 451 SANDIEGOZ 20 Mar 1994 594099
Birth
25 Apr 1994
Death
[Death by: Euthanasia (medical) | Unknown | No
necropsy planned]

708 F 21 Mar 1994 243 457 SD-WAP 21 Mar 1994 694121
Birth
2 Jun 1994
Death
[Death by: Unknown means]

709 F 25 Mar 1994 243 432 SD-WAP 25 Mar 1994 694142
Birth
JOHNSONLA 21 Aug 1998 ______
ltf

710 M 27 Mar 1994 483 480 SANDIEGOZ 27 Mar 1994 594116
Birth
27 Mar 1994
Death
[Death by: Euthanasia (medical) | Unknown | No
necropsy planned]

711 F 27 Mar 1994 483 480 SANDIEGOZ 27 Mar 1994 594117
Birth
16 Mar 1998
Death
[Death by: Infection associated | Incinerate |

You created this PDF from an application that is not licensed to print to novaPDF printer (http://www.novapdf.com)

Generalized ┼ Bacterial]

712 M 27 Mar 1994 483 471 SANDIEGOZ 27 Mar 1994 594118
Birth
27 Mar 1994
Death
[Death by: Euthanasia (medical) ┼ Unknown ┼ No
necropsy planned]

713 F 27 Mar 1994 483 471 SANDIEGOZ 27 Mar 1994 594119
Birth
21 Oct 2001
Death
[Death by: Injury from exhibit mate ┼ Incinerate ┼ Unknown (after
necropsy) ┼ Trauma]

714 M 15 Apr 1994 444 464 MEMPHIS 15 Apr 1994 14457
Birth
19 Nov 1997
Death
[Death by: Unknown means]

715 F 15 Apr 1994 444 464 MEMPHIS 15 Apr 1994 14458
Birth
5 Dec 1994
Death
[Death by: Unknown means]

716 M 15 Apr 1994 192 430 SANDIEGOZ 15 Apr 1994 594155
Birth
15 Apr 1994
Death
[Death by: Unknown means]

717 M 19 Apr 1994 243 469 SD-WAP 19 Apr 1994 694179
Birth
30 Jul 1998
Death
[Death by: Euthanasia (medical) ┼ Incinerate ┼
Unknown (after necropsy)]

718 M 28 Apr 1994 436 421 SANDIEGOZ 28 Apr 1994 594179
Birth
28 Apr 1994
Death
[Death by: Euthanasia (medical) ┼ Unknown ┼ No
necropsy planned]

719 M 29 Apr 1994 436 461 SANDIEGOZ 29 Apr 1994 594181
Birth
29 Apr 1994
Death
[Death by: Euthanasia (medical) ┼ Unknown ┼ No
necropsy planned]

720 F 20 Aug 1994 415 472 ST LOUIS 20 Aug 1994 940823
Birth VIRGIE
OR WILDLF 5 May 1995 94336
Transfer
10 Dec 1996
Death
[Death by: Unknown means]

721 M 20 Aug 1994 415 472 ST LOUIS 20 Aug 1994 940824
Birth
OR WILDLF 5 May 1995 94337
Loan to
26 Apr 1996
Death

You created this PDF from an application that is not licensed to print to novaPDF printer (http://www.novapdf.com)

[Death by: Unknown means]

```
        722    M     25 Aug 1994      243      416   SD-WAP     25 Aug 1994 694547
Birth
                                                                25 Aug 1994
Death
                            [Death by: Unknown means]

        723    F     25 Aug 1994      243      416   SD-WAP     25 Aug 1994 694548
Birth
                                                                25 Aug 1994
Death
                            [Death by: Unknown means]

        724    F     22 Sep 1994      415      475   ST LOUIS   22 Sep 1994 940909
Birth
                                                                22 Sep 1994
Death
                            [Death by: Unknown means]

        725    F     22 Sep 1994      415      475   ST LOUIS   22 Sep 1994 940910
Birth     SASSY
                                                     OR WILDLF   5 May 1995  94335
Transfer
                                                                29 Dec 2003
Death
                            [Death by: Unknown means]

        726    M     23 Sep 1994      467      446   EVANSVLLE  23 Sep 1994 194087
Birth     TIPPY
                                                     SHADOW      8 Oct 1997 ______
ltf

        727    ?     23 Sep 1994      467      446   EVANSVLLE  23 Sep 1994 194088
Birth
                                                                23 Sep 1994
Death
                            [Death by: Unknown means]

        728    F     28 Sep 1994      436      490   SD-WAP     28 Sep 1994 694587
Birth
                                                                 1 Oct 1994
Death
                            [Death by: Unknown means]

        729    M      9 Oct 1994      243      432   SD-WAP      9 Oct 1994 694600
Birth
                                                                29 Aug 1995
Death
                            [Death by: Unknown means]

        730    M      9 Oct 1994      243      432   SD-WAP      9 Oct 1994 694601
Birth
                                                                28 Sep 1995
Death
                            [Death by: Unknown means]

        731    F     11 Oct 1994      467      445   EVANSVLLE  11 Oct 1994 194091
Birth     CANOE
                                                                16 Nov 1996
Death
                            [Death by: Unknown means]

        732    F     15 Sep 1994      192      252   SANDIEGOZ  15 Sep 1994 594408
Birth
                                                                26 Nov 2003
Death
                            [Death by: Unknown means]
```


You created this PDF from an application that is not licensed to print to novaPDF printer (http://www.novapdf.com)

733 F 5 Dec 1994 192 451 SANDIEGOZ 5 Dec 1994 594481
Birth
PALM DES 18 Feb 1997 497003
Loan to
24 Mar 1997
Death
[Death by: Anesth/Restraint assoc + Incinerate + Generalized + Unknown after necropsy]

734 F 26 Jan 1995 436 469 SD-WAP 26 Jan 1995 695016
Birth
8 Jun 1995
Death
[Death by: Euthanasia (medical) + Unknown + No necropsy planned]

735 F 26 Jan 1995 436 469 SD-WAP 26 Jan 1995 695017
Birth
26 Jan 1995
Death
[Death by: Stillbirth + Unknown + No necropsy planned]

736 M 28 Jan 1995 415 472 ST LOUIS 28 Jan 1995 950107
Birth
28 Jan 1995
Death
[Death by: Unknown means]

737 M 28 Jan 1995 415 472 ST LOUIS 28 Jan 1995 950108
Birth
28 Jan 1995
Death
[Death by: Unknown means]

738 ? 29 Jan 1995 436 432 SD-WAP 29 Jan 1995 695022
Birth
29 Jan 1995
Death
[Death by: Stillbirth + Unknown + No necropsy planned]

739 ? 30 Jan 1995 436 432 SD-WAP 30 Jan 1995 695025
Birth
30 Jan 1995
Death
[Death by: Stillbirth + Unknown + No necropsy planned]

740 M 26 Feb 1995 661 244 OR WILDLF 26 Feb 1995 95322
Birth
27 Aug 1999
Death
[Death by: Euthanasia (medical) + Unknown + Generalized + Bacterial]

741 M 28 Feb 1995 206 412 PALM DES 28 Feb 1995 495002
Birth GARTH
OR WILDLF 19 Apr 1996 95387
Loan to
4 Nov 2002
Death
[Death by: Unknown means]

742 M 9 Mar 1995 680 480 LANGLEY 9 Mar 1995 H95004
Birth MV4E
12 Aug 1996
Death
[Death by: Unknown means]

You created this PDF from an application that is not licensed to print to novaPDF printer (http://www.novapdf.com)

743 M 16 Mar 1995 680 471 LANGLEY 16 Mar 1995 H95006
Birth
ALDERGROV 3 Sep 1998 ______
Transfer
~ 1999
Death
[Death by: Unknown means]

744 F 16 Mar 1995 192 252 SANDIEGOZ 16 Mar 1995 595092
Birth TAZA
PALM DES 18 Feb 1997 497004
Loan to

745 M 27 Mar 1995 436 687 SD-WAP 27 Mar 1995 695080
Birth
JOHNSONLA 21 Aug 1998 ______
ltf

746 M 28 Mar 1995 467 481 EVANSVLLE 28 Mar 1995 195011
Birth
31 Mar 1995
Death
[Death by: Unknown means]

747 F 28 Mar 1995 467 481 EVANSVLLE 28 Mar 1995 195012
Birth
30 Mar 1995
Death
[Death by: Unknown means]

748 M 13 Apr 1995 436 686 SD-WAP 13 Apr 1995 695115
Birth
13 Apr 1995
Death
[Death by: Stillbirth | Unknown | No necropsy
planned]

749 M 17 Apr 1995 662 430 SANDIEGOZ 17 Apr 1995 595159
Birth
17 Apr 1995
Death
[Death by: Unknown means]

750 M 19 Apr 1995 415 475 ST LOUIS 19 Apr 1995 950422
Birth
19 Apr 1995
Death
[Death by: Unknown means]

751 F 26 Apr 1995 436 660 SD-WAP 26 Apr 1995 695136
Birth
JOHNSONLA 21 Aug 1998 UNK
ltf

752 F 1 May 1995 467 446 EVANSVLLE 1 May 1995 195028
Birth MARA
27 May 1995
Death
[Death by: Unknown means]

753 F 1 May 1995 467 446 EVANSVLLE 1 May 1995 195029
Birth KIFFA
SHADOW 8 Oct 1997 ______
ltf

754 F 10 May 1995 192 402 SANDIEGOZ 10 May 1995 595220
Birth
10 May 1995

You created this PDF from an application that is not licensed to print to novaPDF printer (http://www.novapdf.com)

Death
[Death by: Infection associated | Unknown | No necropsy planned]

755 F 18 May 1995 680 663 LANGLEY 18 May 1995 H95012
Birth CHELSEA
20 Aug 2001
Death
[Death by: Unknown means]

756 M 25 May 1995 192 461 SANDIEGOZ 25 May 1995 595238
Birth
26 May 1995
Death
[Death by: Unknown means]

757 F 3 Jun 1995 662 421 SANDIEGOZ 3 Jun 1995 595254
Birth
OR WILDLF 10 Dec 1995 95347
Loan to
21 Jan 2005
Death
[Death by: Unknown means]

758 F 3 Jun 1995 662 421 SANDIEGOZ 3 Jun 1995 595255
Birth
OR WILDLF 10 Dec 1995 95346
Loan to
22 May 2001
Death
[Death by: Infection associated | Unknown | Respiratory | Bacterial]

759 M 11 Jun 1995 467 445 EVANSVLLE 11 Jun 1995 195037
Birth
11 Jun 1995
Death
[Death by: Stillbirth | Unknown | No necropsy planned]

760 F 24 Jun 1995 192 451 SANDIEGOZ 24 Jun 1995 595295
Birth JOSEPHINE
ST LOUIS 20 Nov 1996 961114
Loan to

761 F 14 Aug 1995 436 432 SD-WAP 14 Aug 1995 695451
Birth
14 Aug 1995
Death
[Death by: Stillbirth | Unknown | No necropsy planned]

762 F 14 Aug 1995 436 432 SD-WAP 14 Aug 1995 695452
Birth
14 Aug 1995
Death
[Death by: Stillbirth | Unknown | No necropsy planned]

763 F 5 Oct 1995 680 471 LANGLEY 5 Oct 1995 H95016
Birth CAROLINE
21 Jun 2004
Death
[Death by: Unknown means]

764 F 12 Oct 1995 680 480 LANGLEY 12 Oct 1995 H95017
Birth CHRISTY
2 Mar 2002
Death

You created this PDF from an application that is not licensed to print to novaPDF printer (http://www.novapdf.com)

[Death by: Unknown means]

765 F 12 Oct 1995 680 480 LANGLEY 12 Oct 1995 H95018
Birth CATHY
29 Sep 1998
Death
[Death by: Unknown means]

766 M 31 Oct 1995 415 475 ST LOUIS 31 Oct 1995 951043
Birth
18 Mar 1996
Death
[Death by: Unknown means]

767 F 31 Oct 1995 415 475 ST LOUIS 31 Oct 1995 951044
Birth TREAT
OR WILDLF 30 Mar 1996 95382
Loan to
26 Jan 2002
Death
[Death by: Unknown means]

768 M 2 Dec 1995 192 461 SANDIEGOZ 2 Dec 1995 595482
Birth
12 Mar 1998
Death
[Death by: Other/Unknown ┼ Incinerate ┼ Musculoskeletal ┼ Mechanical abnormality]

769 M ~ Jan 1996 473 470 JOHNSONLA ~ Jan 1996 ______
Birth
30 Jun 2004 UNK
ltf

770 F ~ Jan 1996 473 450 JOHNSONLA ~ Jan 1996 ______
Birth
30 Jun 2004 UNK
ltf

771 F ~ Jan 1996 473 450 JOHNSONLA ~ Jan 1996 ______
Birth
30 Jun 2004 UNK
ltf

772 F 1 Jan 1996 680 663 LANGLEY 1 Jan 1996 H96001
Birth MV1F
1 Jan 1996
Death
[Death by: Unknown means]

773 M 27 Jan 1996 192 402 SANDIEGOZ 27 Jan 1996 596018
Birth
2 Feb 1996
Death
[Death by: Unknown means]

774 F 27 Jan 1996 192 402 SANDIEGOZ 27 Jan 1996 596019
Birth ROTA
PALM DES 18 Feb 1997 497005
Loan to

775 F 6 Mar 1996 682 429 WILDS 6 Mar 1996 960306
Birth CRAZY 8
HOGLE 30 May 2002 5224
Transfer

776 M 6 Mar 1996 682 429 WILDS 6 Mar 1996 960307
Birth SKIP
HOGLE 30 May 2002 U02049

You created this PDF from an application that is not licensed to print to novaPDF printer (http://www.novapdf.com)

```
Transfer
                                                               24 May 2005
Death
                           [Death by: Unknown means]

       777   M    14 Mar 1996     662     689  SANDIEGOZ  14 Mar 1996 596072
Birth
                                                               25 May 2000
Death
                           [Death by: Euthanasia (medical) | Incinerate |
Unknown (after necropsy)]

       778   M    15 Mar 1996     206     412  PALM DES   15 Mar 1996 496013
Birth     PIERRE
                                                               11 Feb 2004
Death
                           [Death by: Unknown means]

       779   F    15 Mar 1996     206     412  PALM DES   15 Mar 1996 496014
Birth     JEANNE
                                               OR WILDLF  13 Dec 1996  96429
Loan to

       780   F    23 Mar 1996     682     465  WILDS      23 Mar 1996 960323
Birth
                                                           2 Jan 2001
Death
                           [Death by: Other/Unknown | Bury | Generalized |
Trauma]

       781   F    23 Mar 1996     662     421  SANDIEGOZ  23 Mar 1996 596096
Birth
                                                          23 Mar 1996
Death
                           [Death by: Unknown means]

       782   F    12 Apr 1996     192     252  SANDIEGOZ  12 Apr 1996 596127
Birth
                                                          21 Apr 1996
Death
                           [Death by: Unknown means]

       783   M    13 Apr 1996     682     653  WILDS      13 Apr 1996 960401
Birth     ROCK
                                               JOHNSONLA  21 Dec 1997 ______
ltf

       784   F     7 May 1996     680     471  LANGLEY     7 May 1996 H96017
Birth
                                                          27 Sep 1996
Death
                           [Death by: Unknown means]

       785   F     1 Jun 1996     192     451  SANDIEGOZ   1 Jun 1996 596226
Birth
                                               ST LOUIS   20 Nov 1996 961115
Loan to
                                                          25 Oct 1999
Death
                           [Death by: Unknown means]

       786   F    20 Jul 1996     656     244  OR WILDLF  20 Jul 1996   5235
Birth
                                                          31 Jul 2001
Death
                           [Death by: Unknown means]

       787   M    26 Jul 1996     679     663  LANGLEY    26 Jul 1996 H96016
Birth     CAD
```


You created this PDF from an application that is not licensed to print to novaPDF printer (http://www.novapdf.com)

17 Mar 2000
Death
[Death by: Self-inflicted injuries ┼ Mounted or Preserved: UNK ┼ No necropsy planned]

788 M 30 Jul 1996 656 725 OR WILDLF 30 Jul 1996 96422
Birth
25 Sep 2001
Death
[Death by: Unknown means]

789 F 3 Sep 1996 656 720 OR WILDLF 3 Sep 1996 96420
Birth CINDY
27 Nov 1996
Death
[Death by: Unknown means]

790 F 2 Oct 1996 682 429 WILDS 2 Oct 1996 961001
Birth
3 Oct 1996
Death
[Death by: Unknown means]

791 F 2 Oct 1996 682 429 WILDS 2 Oct 1996 961002
Birth BENTHORN
HOGLE 30 May 2002 5240
Transfer
23 Dec 2002
Death
[Death by: Unknown means]

792 M 18 Oct 1996 682 653 WILDS 18 Oct 1996 961003
Birth
19 Oct 1996
Death
[Death by: Unknown means]

793 F 20 Oct 1996 192 402 SANDIEGOZ 20 Oct 1996 596496
Birth
SHADOW 27 Aug 1998 ______
Loan to
12 Sep 1998
Death
[Death by: Unknown means]

794 M 14 Nov 1996 682 465 WILDS 14 Nov 1996 961107
Birth BLAZE
3 Dec 1997 961107
Transfer
JOHNSONLA 21 Dec 1997 ______
ltf

795 F 23 Nov 1996 679 471 LANGLEY 23 Nov 1996 H96024
Birth CELINE
23 Jan 1997
Death
[Death by: Unknown means]

796 F 27 Nov 1996 415 475 ST LOUIS 27 Nov 1996 961126
Birth CRANBERRY
OR WILDLF 13 May 1997 96432
Loan to
1 Oct 2006
Death
[Death by: Euthanasia (medical) ┼ Rendered ┼ Generalized ┼ Bacterial]

797 M 15 Jan 1997 741 758 OR WILDLF 15 Jan 1997 97433
Birth

You created this PDF from an application that is not licensed to print to novaPDF printer (http://www.novapdf.com)

14 Jun 2005
Death
[Death by: Injury from exhibit mate + Unknown +
Generalized + Trauma]

798 F 21 Jan 1997 662 689 SANDIEGOZ 21 Jan 1997 597015
Birth
21 Jan 1997
Death
[Death by: Stillbirth + Unknown + No necropsy
planned]

799 F 21 Jan 1997 662 689 SANDIEGOZ 21 Jan 1997 597016
Birth
21 Jan 1997
Death
[Death by: Stillbirth + Unknown + No necropsy
planned]

800 M 20 Feb 1997 662 421 SANDIEGOZ 20 Feb 1997 597060
Birth
20 Feb 1997
Death
[Death by: Stillbirth + Unknown + No necropsy
planned]

801 F 20 Feb 1997 662 421 SANDIEGOZ 20 Feb 1997 597061
Birth
SHADOW 27 Aug 1998 _______
ltf

802 F 1 Mar 1997 741 244 OR WILDLF 1 Mar 1997 97434
Birth
31 Dec 2007
Death
[Death by: Unknown means]

803 M 1 Mar 1997 741 244 OR WILDLF 1 Mar 1997 97435
Birth
PEACE RV 22 Apr 2000 200003
Transfer

804 F 11 Mar 1997 741 725 OR WILDLF 11 Mar 1997 97436
Birth
2 Jan 2007
Death
[Death by: Infection associated + Incinerate +
Generalized + Bacterial]

805 M 11 Mar 1997 741 725 OR WILDLF 11 Mar 1997 97437
Birth
3 Jul 2005
Death
[Death by: Infection associated + Rendered +
Generalized + Bacterial]

806 F 16 Mar 1997 699 252 SANDIEGOZ 16 Mar 1997 597085
Birth
SHADOW 27 Aug 1998 _______
ltf

807 F 17 Mar 1997 741 767 OR WILDLF 17 Mar 1997 97438
Birth
16 Apr 2003
Death
[Death by: Unknown means]

808 M 21 Mar 1997 662 713 SANDIEGOZ 21 Mar 1997 597099
Birth

You created this PDF from an application that is not licensed to print to novaPDF printer (http://www.novapdf.com)

26 Mar 1997
Death
[Death by: Unknown means]

809 F 24 Mar 1997 UNK 733 PALM DES 24 Mar 1997 497009
Birth
24 Mar 1997
Death
[Death by: Unknown means]

810 M 24 Mar 1997 662 706 SANDIEGOZ 24 Mar 1997 597105
Birth
24 Mar 1997
Death
[Death by: Stillbirth + Unknown + No necropsy
planned]

811 M 24 Mar 1997 699 732 SANDIEGOZ 24 Mar 1997 597112
Birth
4 Jul 1997
Death
[Death by: Unknown means]

812 F 28 Mar 1997 699 461 SANDIEGOZ 28 Mar 1997 597126
Birth
SHADOW 27 Aug 1998 UNKK
ltf

813 F 8 Apr 1997 699 711 SANDIEGOZ 8 Apr 1997 597146
Birth

814 M 11 Apr 1997 192 451 SANDIEGOZ 11 Apr 1997 597149
Birth
19 May 2003
Death
[Death by: Unknown means]

815 F 11 Apr 1997 192 451 SANDIEGOZ 11 Apr 1997 597150
Birth
15 Mar 2005
Death
[Death by: Unknown means]

816 M 12 Apr 1997 680 663 LANGLEY 12 Apr 1997 H97006
Birth CANE
27 Aug 1999
Death
[Death by: Other/Unknown + Incinerate + No necropsy
planned]

817 F 12 Apr 1997 680 663 LANGLEY 12 Apr 1997 H97007
Birth CANDY
5 Jan 1998
Death
[Death by: Unknown means]

818 M 10 May 1997 192 402 SANDIEGOZ 10 May 1997 597195
Birth
SHADOW 27 Aug 1998 UNK
ltf

819 M 10 May 1997 192 402 SANDIEGOZ 10 May 1997 597196
Birth
SHADOW 27 Aug 1998 UNK
ltf

820 M 17 May 1997 743 480 LANGLEY 17 May 1997 H97011
Birth CASPER
UARAB EM 24 Apr 2005 UNK

You created this PDF from an application that is not licensed to print to novaPDF printer (http://www.novapdf.com)

821 M 17 May 1997 743 480 LANGLEY 17 May 1997 H97012
Birth CODY
30 Jan 2002
Death
[Death by: Unknown means]

822 M 13 Jun 1997 415 475 ST LOUIS 13 Jun 1997 970625
Birth JINX
1 Dec 2005
Death
[Death by: Unknown means]

823 F 27 Jun 1997 656 412 OR WILDLF 27 Jun 1997 97448
Birth
19 Dec 2006
Death
[Death by: Unknown means]

824 F 27 Jun 1997 656 412 OR WILDLF 27 Jun 1997 97449
Birth
PEACE RV 22 Apr 2000 200005
Transfer

825 M 8 Jul 1997 741 690 OR WILDLF 8 Jul 1997 97450
Birth
PEACE RV 22 Apr 2000 200004
Transfer

826 M 10 Jul 1997 743 471 LANGLEY 10 Jul 1997 H97020
Birth CHARLIE
22 Oct 2001
Death
[Death by: Infection associated ┼ Unknown ┼ Unknown
(after necropsy)]

827 M 10 Jul 1997 743 471 LANGLEY 10 Jul 1997 H97021
Birth
15 Jul 1997
Death
[Death by: Unknown means]

828 F 1 Aug 1997 427 429 WILDS 1 Aug 1997 970801
Birth
1 Aug 1997
Death
[Death by: Unknown means]

829 F 25 Aug 1997 682 465 WILDS 25 Aug 1997 970848
Birth
16 Oct 1997
Death
[Death by: Unknown means]

830 M 27 Aug 1997 682 653 WILDS 27 Aug 1997 970849
Birth
16 Oct 1997
Death
[Death by: Unknown means]

831 F 27 Aug 1997 682 653 WILDS 27 Aug 1997 970850
Birth
27 Aug 1997
Death
[Death by: Unknown means]

832 F 29 Sep 1997 743 763 LANGLEY 29 Sep 1997 H97027
Birth CRITTER

You created this PDF from an application that is not licensed to print to novaPDF printer (http://www.novapdf.com)

13 Apr 2003
Death
[Death by: Unknown means]

833 M 29 Sep 1997 743 763 LANGLEY 29 Sep 1997 H97028
Birth
29 Sep 1997
Death
[Death by: Unknown means]

834 M 8 Oct 1997 743 764 LANGLEY 8 Oct 1997 H97029
Birth
8 Oct 1997
Death
[Death by: Unknown means]

835 M 8 Oct 1997 743 764 LANGLEY 8 Oct 1997 H97030
Birth
8 Oct 1997
Death
[Death by: Unknown means]

836 F 14 Nov 1997 662 421 SANDIEGOZ 14 Nov 1997 597424
Birth
22 Nov 2001
Death
[Death by: Injury from exhibit mate + Incinerate +
Unknown (after necropsy)]

837 F 17 Nov 1997 743 765 LANGLEY 17 Nov 1997 H97034
Birth
17 Nov 1997
Death
[Death by: Unknown means]

838 M 23 Dec 1997 743 663 LANGLEY 23 Dec 1997 H97037
Birth CLAUS
5 Jan 2005
Death
[Death by: Unknown means]

839 M 14 Feb 1998 777 713 SANDIEGOZ 14 Feb 1998 598021
Birth
15 Feb 1998
Death
[Death by: Unknown means]

840 M 14 Feb 1998 192 451 SANDIEGOZ 14 Feb 1998 598023
Birth
15 Feb 1998
Death
[Death by: Unknown means]

841 M 14 Feb 1998 192 451 SANDIEGOZ 14 Feb 1998 598024
Birth
15 Feb 1998
Death
[Death by: Unknown means]

842 F 14 Feb 1998 777 713 SANDIEGOZ 14 Feb 1998 598022
Birth
11 Sep 2001
Death
[Death by: Euthanasia (medical) + Incinerate +
Unknown (after necropsy)]

843 F 16 Feb 1998 444 440 MEMPHIS 16 Feb 1998 17456
Birth
16 Feb 1998

You created this PDF from an application that is not licensed to print to novaPDF printer (http://www.novapdf.com)

Death
[Death by: Unknown means]

844 M 9 Mar 1998 699 252 SANDIEGOZ 9 Mar 1998 598045
Birth
10 Mar 1998
Death
[Death by: Unknown means]

845 M 14 Mar 1998 699 711 SANDIEGOZ 14 Mar 1998 598057
Birth
14 Mar 1998
Death
[Death by: Unknown means]

846 M 18 Mar 1998 699 812 SANDIEGOZ 18 Mar 1998 598067
Birth
18 Mar 1998
Death
[Death by: Other/Unknown ┼ Incinerate ┼ No necropsy planned]

847 F 30 Mar 1998 699 732 SANDIEGOZ 30 Mar 1998 598074
Birth
27 Jul 2003
Death
[Death by: Unknown means]

848 F 30 Mar 1998 699 732 SANDIEGOZ 30 Mar 1998 598075
Birth
6 Sep 2003
Death
[Death by: Unknown means]

849 M 8 Apr 1998 787 764 LANGLEY 8 Apr 1998 H98005
Birth CALVIN
2 Jun 2001
Death
[Death by: Unknown means]

850 M 19 Apr 1998 680 755 LANGLEY 19 Apr 1998 H98006
Birth CHRIS
ALDERGROV 3 Sep 1998 ______
Transfer
~ 1999
Death
[Death by: Unknown means]

851 M 19 Apr 1998 680 755 LANGLEY 19 Apr 1998 H98007
Birth CROSS
EDWARDS 3 Sep 1998 ______
Transfer
15 Sep 1999
Death
[Death by: Unknown means]

852 F 27 Apr 1998 787 763 LANGLEY 27 Apr 1998 H98011
Birth CASSIE
30 Dec 2001
Death
[Death by: Unknown means]

853 F 27 Apr 1998 787 763 LANGLEY 27 Apr 1998 H98012
Birth CUE
7 May 1999
Death
[Death by: Injury from exhibit mate ┼ Mounted or Preserved: ┼ No necropsy planned]

You created this PDF from an application that is not licensed to print to novaPDF printer (http://www.novapdf.com)

```
       854   F     6 May 1998     778     774  PALM DES    6 May 1998 498013
Birth     THUNDER
                                               PEACE RV   31 Oct 2000 200050
Transfer

       855   F     9 May 1998     778     744  PALM DES    9 May 1998 498016
Birth     PIE
                                               PEACE RV   31 Oct 2000 200051
Transfer
                                                           2 Aug 2001
Death
                           [Death by: Environ/Behav conditions + Bury +
Digestive + Toxicity]

       856   F    31 May 1998     777     421  SANDIEGOZ  31 May 1998 598197
Birth
                                               PEACE RV   19 Nov 2004    UNK
Transfer

       857   F    31 May 1998     777     421  SANDIEGOZ  31 May 1998 598198
Birth
                                               PEACE RV   19 Nov 2004    UNK
Transfer

       858   F    10 Jun 1998     699     461  SANDIEGOZ  10 Jun 1998 598219
Birth
                                               JOHNSONLA  22 Aug 2007    UNK
ltf

       859   F    26 Jun 1998     788     757  OR WILDLF  26 Jun 1998  98494
Birth
                                                           5 May 2003
Death
                           [Death by: Unknown means]

       860   F    26 Jun 1998     788     757  OR WILDLF  26 Jun 1998  98495
Birth
                                               PEACE RV   22 Apr 2000 200007
Transfer

       861   F    28 Jun 1998     788     690  OR WILDLF  28 Jun 1998  98493
Birth
                                               PEACE RV   22 Apr 2000 200006
Transfer

       862   F     5 Jul 1998     656     244  OR WILDLF   5 Jul 1998  98498
Birth
                                               PEACE RV   22 Apr 2000 200008
Transfer
                                                          20 May 2000
Death
                           [Death by: Other/Unknown + Bury + Unknown (after
necropsy)]

       863   M     5 Jul 1998     656     758  OR WILDLF   5 Jul 1998  98499
Birth
                                                          16 Jul 1998
Death
                           [Death by: Unknown means]

       864   F     5 Jul 1998     656     758  OR WILDLF   5 Jul 1998  98500
Birth
                                                           9 Jun 2002
Death
                           [Death by: Unknown means]

       865   F     8 Jul 1998     656     767  OR WILDLF   8 Jul 1998  98529
Birth
                                                          10 Jul 1998
```


You created this PDF from an application that is not licensed to print to novaPDF printer (http://www.novapdf.com)

Death
[Death by: Unknown means]

866 M 12 Jul 1998 680 663 LANGLEY 12 Jul 1998 H98023
Birth COBY
7 Nov 2002
Death
[Death by: Unknown means]

867 F 23 Jul 1998 777 706 SANDIEGOZ 23 Jul 1998 598058
Birth
24 Jul 1998
Death
[Death by: Injury from exhibit mate + Incinerate +
Generalized + Trauma]

868 M 19 Aug 1998 777 713 SANDIEGOZ 19 Aug 1998 598337
Birth
20 Aug 1998
Death
[Death by: Unknown means]

869 F 19 Aug 1998 777 713 SANDIEGOZ 19 Aug 1998 598338
Birth
SHADOW 30 Apr 2000 ______
ltf

870 F 15 Nov 1998 192 451 SANDIEGOZ 15 Nov 1998 598420
Birth
20 Feb 2002
Death
[Death by: Unknown means]

871 F 8 Feb 1998 415 760 ST LOUIS 8 Feb 1998 980207
Birth SOPHIE
PROVIDNCE 12 May 1999 991052
Transfer
PALM DES 26 Apr 2006 406007
Transfer

872 F 6 Dec 1998 415 785 ST LOUIS 6 Dec 1998 981222
Birth NIC
PEACE RV 22 Oct 1999 990073
Transfer

873 F 6 Jan 1999 777 706 SANDIEGOZ 6 Jan 1999 599005
Birth
SHADOW 10 Apr 2000 ______
ltf

874 M 7 Jan 1999 777 689 SANDIEGOZ 7 Jan 1999 599006
Birth
5 Apr 2002
Death
[Death by: Unknown means]

875 M 14 Feb 1999 787 764 LANGLEY 14 Feb 1999 H99003
Birth CALUM
18 May 2001
Death
[Death by: Unknown means]

876 F 14 Feb 1999 787 764 LANGLEY 14 Feb 1999 H99004
Birth CELESTE
28 Apr 2006
Death
[Death by: Unknown means]

877 ? 17 Feb 1999 778 774 PALM DES 17 Feb 1999 499009

You created this PDF from an application that is not licensed to print to novaPDF printer (http://www.novapdf.com)

Birth
17 Feb 1999
Death
[Death by: Premature birth + Incinerate + Generalized + Unknown after necropsy]

878 F 19 Feb 1999 UNK 732 SANDIEGOZ 19 Feb 1999 599032
Birth
SHADOW 30 Apr 2000 ______
ltf

879 F 19 Feb 1999 UNK 732 SANDIEGOZ 19 Feb 1999 599033
Birth
SHADOW 30 Apr 2000 ______
ltf

880 M 20 Feb 1999 787 763 LANGLEY 20 Feb 1999 H99006
Birth CONRAD
28 Jan 2000
Death
[Death by: Injury from exhibit mate + Mounted or Preserved: + No necropsy planned]

881 F 18 Mar 1999 699 461 SANDIEGOZ 18 Mar 1999 599068
Birth
ST LOUIS 31 May 2000 100255
Transfer
PEACE RV 8 Nov 2002 202085
Transfer

882 F 22 Mar 1999 787 663 LANGLEY 22 Mar 1999 H99007
Birth
UARAB EM 24 Apr 2005 UNK
ltf

883 ? 5 Apr 1999 444 440 MEMPHIS 5 Apr 1999 18424
Birth
5 Apr 1999
Death
[Death by: Premature birth + Unknown + Unknown (after necropsy)]

884 M 13 Apr 1999 787 853 LANGLEY 13 Apr 1999 H99010
Birth
15 Apr 1999
Death
[Death by: Other/Unknown + Incinerate + No necropsy planned]

885 M 23 Apr 1999 699 815 SANDIEGOZ 23 Apr 1999 599119
Birth
25 Apr 1999
Death
[Death by: Unknown means]

886 M 3 May 1999 778 744 PALM DES 3 May 1999 499033
Birth RUDY/VAUGH
PROVIDNCE 26 Apr 2000 101200
Transfer
PALM DES 26 Apr 2006 499033
Transfer
7 Aug 2006
Death
[Death by: Injury from predator + Incinerate + Generalized + Unknown after necropsy]

887 F 6 May 1999 427 775 WILDS 6 May 1999 990501
Birth
10 Jul 2000

You created this PDF from an application that is not licensed to print to novaPDF printer (http://www.novapdf.com)

Death
[Death by: Other/Unknown + Bury + Generalized + Unknown after necropsy]

888 M 6 May 1999 427 780 WILDS 6 May 1999 990502
Birth
9 May 1999
Death
[Death by: Euthanasia (medical) + Unknown + Unknown (after necropsy)]

889 F 6 May 1999 427 780 WILDS 6 May 1999 990503
Birth
8 May 1999
Death
[Death by: Unknown means]

890 M 7 May 1999 415 760 ST LOUIS 7 May 1999 990512
Birth
SHADOW 2 Jun 2000 ______
ltf

891 F 9 Jun 1999 797 824 OR WILDLF 9 Jun 1999 99543
Birth ROSIE
PEACE RV 22 Apr 2000 200009
Transfer

892 F 9 Jun 1999 797 824 OR WILDLF 9 Jun 1999 99544
Birth
PEACE RV 22 Apr 2000 200010
Transfer
2 Aug 2001
Death
[Death by: Environ/Behav conditions + Bury + Digestive + Toxicity]

893 M 10 Jun 1999 797 807 OR WILDLF 10 Jun 1999 99545
Birth
27 Apr 2005
Death
[Death by: Unknown means]

894 M 10 Jun 1999 797 807 OR WILDLF 10 Jun 1999 99546
Birth
19 Oct 2004
Death
[Death by: Unknown means]

895 M 13 Jun 1999 797 802 OR WILDLF 13 Jun 1999 99547
Birth
2 Aug 2003
Death
[Death by: Unknown means]

896 F 13 Jun 1999 797 802 OR WILDLF 13 Jun 1999 99548
Birth
11 May 2004
Death
[Death by: Unknown means]

897 F 15 Jun 1999 797 796 OR WILDLF 15 Jun 1999 99549
Birth
15 May 2002
Death
[Death by: Unknown means]

898 F 15 Jun 1999 797 796 OR WILDLF 15 Jun 1999 99550
Birth

You created this PDF from an application that is not licensed to print to novaPDF printer (http://www.novapdf.com)

899 F 18 Jun 1999 797 823 OR WILDLF 18 Jun 1999 99551
Birth
6 Oct 2006
Death
[Death by: Infection associated + Incinerate +
Generalized + Bacterial]

900 M 18 Jun 1999 797 823 OR WILDLF 18 Jun 1999 99552
Birth
3 Nov 2003
Death
[Death by: Unknown means]

901 M 29 Jun 1999 797 779 OR WILDLF 29 Jun 1999 99555
Birth
23 Feb 2002
Death
[Death by: Unknown means]

902 F 1 Aug 1999 797 804 OR WILDLF 1 Aug 1999 99578
Birth
30 Aug 2005
Death
[Death by: Injury from predator + Rendered +
Generalized + Trauma]

903 M 1 Aug 1999 797 804 OR WILDLF 1 Aug 1999 99579
Birth
3 Dec 2003
Death
[Death by: Unknown means]

904 M 20 Sep 1999 787 755 LANGLEY 20 Sep 1999 H99016
Birth CAMARL
UARAB EM 24 Apr 2005 UNK
ltf

905 M 11 Oct 1999 816 832 LANGLEY 11 Oct 1999 H99017
Birth CRICKET
17 Jan 2000
Death
[Death by: Injury from exhibit mate + Mounted or Preserved: UNK + No
necropsy planned]

906 M 16 Oct 1999 787 764 LANGLEY 16 Oct 1999 H99018
Birth COBALI
~15 Nov 2000
Death
[Death by: Unknown means]

907 F 16 Oct 1999 787 764 LANGLEY 16 Oct 1999 H99019
Birth CHALEEN
23 Aug 2003
Death
[Death by: Unknown means]

908 M 25 Oct 1999 415 785 ST LOUIS 25 Oct 1999 991041
Birth
25 Oct 1999
Death
[Death by: Premature birth + Unknown + Unknown (after
necropsy)]

909 F 25 Oct 1999 415 785 ST LOUIS 25 Oct 1999 991042
Birth
25 Oct 1999
Death
[Death by: Premature birth + Unknown + Unknown (after
necropsy)]

You created this PDF from an application that is not licensed to print to novaPDF printer (http://www.novapdf.com)

910 F 8 Dec 1999 820 663 LANGLEY 8 Dec 1999 H99023
Birth CHLOE
23 Mar 2001
Death
[Death by: Euthanasia (medical) + Unknown + Unknown (after necropsy)]

911 M 8 Dec 1999 820 663 LANGLEY 8 Dec 1999 H99024
Birth CALE
3 Sep 2001
Death
[Death by: Unknown means]

912 M ~ 1998 UNK UNK BULVERDE ~ 1998 ______
Birth
PEACE RV 20 Apr 1999 990013
Transfer
13 Aug 1999
Death
[Death by: Infection associated + Bury + Digestive + Unknown after necropsy]

913 F ~ 1998 UNK UNK BULVERDE ~ 1998 ______
Birth
PEACE RV 20 Apr 1999 990014
Transfer
16 Jun 1999
Death
[Death by: Unknown means]

914 F ~ 1998 UNK UNK BULVERDE ~ 1998 ______
Birth
PEACE RV 20 Apr 1999 990015
Transfer

915 F 11 Jan 2000 787 763 LANGLEY 11 Jan 2000 H00001
Birth
12 Jan 2000
Death
[Death by: Other/Unknown + Mounted or Preserved: UNK + No necropsy planned]

916 F 14 Mar 2000 778 744 PALM DES 14 Mar 2000 400007
Birth
18 Mar 2000
Death
[Death by: Other/Unknown + Incinerate + Generalized + Metabolism]

917 F 14 Mar 2000 778 744 PALM DES 14 Mar 2000 400008
Birth BRANDY
PEACE RV 31 Oct 2000 200052
Transfer

918 F 14 Mar 2000 778 774 PALM DES 14 Mar 2000 400010
Birth ADJANA
PEACE RV 31 Oct 2000 200053
Transfer

919 F 5 Apr 2000 787 755 LANGLEY 5 Apr 2000 H00005
Birth CARLILL
~15 Oct 2000
Death
[Death by: Unknown means]

920 F 5 Apr 2000 787 755 LANGLEY 5 Apr 2000 H00006
Birth CARMEN
~ Jan 2003

You created this PDF from an application that is not licensed to print to novaPDF printer (http://www.novapdf.com)

Death
[Death by: Unknown means]

921 M 6 Apr 2000 415 760 ST LOUIS 6 Apr 2000 100116
Birth
PEACE RV 8 Nov 2002 202084
Transfer

922 F 19 Apr 2000 849 832 LANGLEY 19 Apr 2000 H00010
Birth
19 Apr 2000
Death
[Death by: Stillbirth ┼ Mounted or Preserved: UNK ┼
No necropsy planned]

923 F 19 Apr 2000 849 832 LANGLEY 19 Apr 2000 H00011
Birth
8 Jul 2004
Death
[Death by: Unknown means]

924 F 2 May 2000 787 764 LANGLEY 2 May 2000 H00012
Birth CAILEY
13 Jan 2002
Death
[Death by: Unknown means]

925 M 20 May 2000 UNK 824 PEACE RV 20 May 2000 200023
Birth
12 Mar 2002
Death
[Death by: Unknown means]

926 F 20 May 2000 UNK 824 PEACE RV 20 May 2000 200024
Birth

927 F 30 May 2000 UNK 860 PEACE RV 30 May 2000 200038
Birth

928 F 2 Jun 2000 797 823 OR WILDLF 2 Jun 2000 200620
Birth
31 May 2006
Death
[Death by: Unknown means]

929 M 2 Jun 2000 797 823 OR WILDLF 2 Jun 2000 200621
Birth
9 Jan 2003
Death
[Death by: Unknown means]

930 F 16 Jun 2000 797 767 OR WILDLF 16 Jun 2000 200623
Birth
27 Dec 2002
Death
[Death by: Unknown means]

931 F 21 Jun 2000 797 725 OR WILDLF 21 Jun 2000 200624
Birth SUMMER
3 Jul 2000
Death
[Death by: Other/Unknown ┼ Unknown ┼ Generalized ┼
Nutrition]

932 M 21 Jun 2000 797 725 OR WILDLF 21 Jun 2000 200625
Birth SOLSTICE
8 Jul 2000
Death
[Death by: Other/Unknown ┼ Unknown ┼ Generalized ┼

You created this PDF from an application that is not licensed to print to novaPDF printer (http://www.novapdf.com)

Nutrition]

933 M 24 Jun 2000 820 663 LANGLEY 24 Jun 2000 H00014
Birth CADEN
31 Aug 2002
Death
[Death by: Unknown means]

934 M 24 Jun 2000 820 663 LANGLEY 24 Jun 2000 H00015
Birth CINSI
17 Mar 2002
Death
[Death by: Unknown means]

935 F 4 Jul 2000 797 690 OR WILDLF 4 Jul 2000 200631
Birth
29 Jan 2001
Death
[Death by: Other/Unknown + Unknown + Digestive +
Unknown after necropsy]

936 M 4 Jul 2000 797 690 OR WILDLF 4 Jul 2000 200632
Birth
25 Feb 2002
Death
[Death by: Unknown means]

937 F 20 Jul 2000 427 775 WILDS 20 Jul 2000 MM0709
Birth AMY
HOGLE 30 May 2002 5386
Transfer

938 F ~15 Sep 2000 821 763 LANGLEY ~15 Sep 2000 H00018
Birth
~ 5 Dec 2000
Death
[Death by: Unknown means]

939 F 19 Oct 2000 820 832 LANGLEY 19 Oct 2000 H00021
Birth
~10 Nov 2000
Death
[Death by: Unknown means]

940 M 12 Dec 2000 825 872 PEACE RV 12 Dec 2000 200055
Birth
23 Jul 2001
Death
[Death by: Environ/Behav conditions + Bury +
Digestive + Toxicity]

941 M 11 Jan 2001 849 663 LANGLEY 11 Jan 2001 H01002
Birth
12 Jan 2001
Death
[Death by: Unknown means]

942 F 11 Jan 2001 849 663 LANGLEY 11 Jan 2001 H01003
Birth
12 Jan 2001
Death
[Death by: Unknown means]

943 F 14 Jan 2001 821 764 LANGLEY 14 Jan 2001 H01004
Birth
17 Jan 2001
Death
[Death by: Unknown means]

You created this PDF from an application that is not licensed to print to novaPDF printer (http://www.novapdf.com)

944 F 14 Jan 2001 821 764 LANGLEY 14 Jan 2001 H01005
Birth CARLA
12 Feb 2004
Death
[Death by: Unknown means]

945 M 17 Jan 2001 803 891 PEACE RV 17 Jan 2001 201001
Birth

946 F 23 Jan 2001 803 860 PEACE RV 23 Jan 2001 201002
Birth

947 M 25 Jan 2001 821 755 LANGLEY 25 Jan 2001 H01007
Birth
25 Jan 2001
Death
[Death by: Unknown means]

948 F 25 Jan 2001 821 755 LANGLEY 25 Jan 2001 H01008
Birth CALAIS
11 Mar 2002
Death
[Death by: Unknown means]

949 M 30 Mar 2001 886 871 PROVIDNCE 30 Mar 2001 100106
Birth ZACK
PALM DES 26 Apr 2006 406006
Transfer
31 Mar 2007
Death
[Death by: Unknown means]

950 F 16 Apr 2001 821 763 LANGLEY 16 Apr 2001 H01011
Birth CLARA
14 Jul 2005
Death
[Death by: Unknown means]

951 F 20 Apr 2001 699 857 SANDIEGOZ 20 Apr 2001 501110
Birth
28 Aug 2005
Death
[Death by: Unknown means]

952 M 25 May 2001 UNK 725 OR WILDLF 25 May 2001 201671
Birth
22 Apr 2003
Death
[Death by: Unknown means]

953 F 25 May 2001 UNK 725 OR WILDLF 25 May 2001 201672
Birth

954 F 31 May 2001 825 861 PEACE RV 31 May 2001 201032
Birth
14 Jul 2001
Death
[Death by: Environ/Behav conditions + Bury +
Digestive + Toxicity]

955 M 1 Jun 2001 825 854 PEACE RV 1 Jun 2001 201033
Birth

956 M 1 Jun 2001 825 854 PEACE RV 1 Jun 2001 201034
Birth

957 F 13 Jul 2001 820 832 LANGLEY 13 Jul 2001 H01016
Birth CLARISSE

You created this PDF from an application that is not licensed to print to novaPDF printer (http://www.novapdf.com)

	958	F	16 Jul 2001	825	872	PEACE RV	16 Jul 2001	201037
Birth								
	959	M	11 Aug 2001	825	860	PEACE RV	11 Aug 2001	201041
Birth								
							14 Jun 2003	
Death								
			[Death by: Unknown means]					
	960	F	11 Aug 2001	825	860	PEACE RV	11 Aug 2001	201042
Birth								
	961	F	17 Aug 2001	849	882	LANGLEY	17 Aug 2001	H01020
Birth								
							19 Aug 2001	
Death								
			[Death by: Unknown means]					
	962	F	21 Oct 2001	849	663	LANGLEY	21 Oct 2001	H01034
Birth								
							12 May 2002	
Death								
			[Death by: Unknown means]					
	963	F	24 Oct 2001	821	763	LANGLEY	24 Oct 2001	H01035
Birth								
							24 Oct 2001	
Death								
			[Death by: Unknown means]					
	964	M	19 May 2003	561	576	ALMERIA	19 May 2003	EE964
Birth								
							5 Dec 2005	
Death								
			[Death by: Unknown means]					
	965	M	19 May 2003	594	555	ALMERIA	19 May 2003	EE965
Birth								
							17 May 2004	
Death								
			[Death by: Unknown means]					
	966	F	19 May 2003	594	555	ALMERIA	19 May 2003	EE966
Birth								
							24 Sep 2006	
Death								
			[Death by: Unknown means]					
	967	M	20 May 2003	561	577	ALMERIA	20 May 2003	EE967
Birth								
							28 May 2003	
Death								
			[Death by: Unknown means]					
	968	M	20 May 2003	561	577	ALMERIA	20 May 2003	EE968
Birth								
							27 May 2003	
Death								
			[Death by: Unknown means]					
	969	M	22 May 2003	584	547	ALMERIA	22 May 2003	EE969
Birth								
							24 May 2003	
Death								
			[Death by: Unknown means]					
	970	M	28 May 2003	594	539	ALMERIA	28 May 2003	EE970
Birth								
						LUGO	20 Feb 2013	UNK

You created this PDF from an application that is not licensed to print to novaPDF printer (http://www.novapdf.com)

Transfer

971 F 28 May 2003 594 539 ALMERIA 28 May 2003 EE971
Birth

972 F 27 Jun 2003 256 566 ALMERIA 27 Jun 2003 EE972
Birth
22 Sep 2006
Death
[Death by: Injury from predator | Incinerate | No
necropsy planned]

973 ? 16 Jan 2002 821 764 LANGLEY 16 Jan 2002 H02001
Birth
16 Jan 2002
Death
[Death by: Unknown means]

974 ? 16 Jan 2002 821 764 LANGLEY 16 Jan 2002 H02002
Birth
18 Jan 2002
Death
[Death by: Unknown means]

975 F 2 Mar 2002 820 832 LANGLEY 2 Mar 2002 H02004
Birth
2 Mar 2002
Death
[Death by: Unknown means]

976 M 2 Mar 2002 820 832 LANGLEY 2 Mar 2002 H02005
Birth CHUCK

977 M 9 Mar 2002 699 857 SANDIEGOZ 9 Mar 2002 502041
Birth
27 Jul 2004
Death
[Death by: Unknown means]

978 M 18 Mar 2002 778 774 PALM DES 18 Mar 2002 402004
Birth
21 Mar 2002
Death
[Death by: Unknown means]

979 F 8 May 2002 821 907 LANGLEY 8 May 2002 H02010
Birth
11 Jan 2005
Death
[Death by: Unknown means]

980 F 8 May 2002 821 907 LANGLEY 8 May 2002 H02011
Birth CANDY
9 Apr 2005
Death
[Death by: Unknown means]

981 M 25 May 2002 820 923 LANGLEY 25 May 2002 H02014
Birth
25 May 2002
Death
[Death by: Unknown means]

982 M 12 Jun 2002 825 861 PEACE RV 12 Jun 2002 202060
Birth

983 M 11 Aug 2002 822 760 ST LOUIS 11 Aug 2002 101865
Birth PRESLEY
PEACE RV 13 Jun 2003 203039

You created this PDF from an application that is not licensed to print to novaPDF printer (http://www.novapdf.com)

Transfer

984 F 11 Aug 2002 822 760 ST LOUIS 11 Aug 2002 101866

Birth LISA MARIE

PEACE RV 12 Jun 2003 203040

Transfer

985 F 11 Sep 2002 820 832 LANGLEY 11 Sep 2002 H02022

Birth CELSEY

28 Jun 2006

Death

[Death by: Unknown means]

986 M 18 Sep 2002 825 891 PEACE RV 18 Sep 2002 202070

Birth

20 Sep 2002

Death

[Death by: Unknown means]

987 M 26 Sep 2002 904 663 LANGLEY 26 Sep 2002 H02023

Birth

18 Jul 2003

Death

[Death by: Unknown means]

988 M 9 Oct 2002 933 763 LANGLEY 9 Oct 2002 H02025

Birth

11 Oct 2002

Death

[Death by: Unknown means]

989 M 1 Dec 2002 814 706 SANDIEGOZ 1 Dec 2002 502334

Birth CHUPEPICO

JOHNSONLA 14 May 2007 UNK

ltf

990 F 23 Apr 2003 602 614 BOUKORNIN 23 Apr 2003 BO990

Birth

991 F 25 Oct 2003 565 321 ALMERIA 25 Oct 2003 EE991

Birth INES

992 M 7 Nov 2003 565 385 ALMERIA 7 Nov 2003 EE992

Birth

20 Nov 2003

Death

[Death by: Unknown means]

993 M 25 Dec 2003 565 347 ALMERIA 25 Dec 2003 EE993

Birth

18 Jan 2004

Death

[Death by: Unknown means]

994 M 5 Jan 2004 584 592 ALMERIA 5 Jan 2004 EE994

Birth MANRESANO

12 Sep 2008

Death

[Death by: Unknown means]

995 F 5 Jan 2004 584 592 ALMERIA 5 Jan 2004 EE995

Birth ISABEL

996 F 14 Jan 2004 584 591 ALMERIA 14 Jan 2004 EE996

Birth YOLANDA

997 M 8 Feb 2004 584 390 ALMERIA 8 Feb 2004 EE997

Birth JESUS

26 Feb 2004

You created this PDF from an application that is not licensed to print to novaPDF printer (http://www.novapdf.com)

Death
[Death by: Unknown means]

998 M 23 Feb 2004 583 576 ALMERIA 23 Feb 2004 EE998
Birth JULI
4 Feb 2008
Death
[Death by: Unknown means]

999 F 23 Feb 2004 583 576 ALMERIA 23 Feb 2004 EE
Birth RODRI
12 Mar 2008
Death
[Death by: Other/Unknown + Unknown + Reproductive + Unknown after necropsy]

1000 M 1 Mar 2004 594 588 ALMERIA 1 Mar 2004 EE1000
Birth JOAQUI
LA LAJITA 27 Nov 2006 LA1000
Transfer
18 May 2011
Death
[Death by: Unknown means]

1001 M 1 Mar 2004 594 555 ALMERIA 1 Mar 2004 EE1001
Birth ISABELO
11 Sep 2009
Death
[Death by: Unknown means]

1002 M 1 Mar 2004 594 555 ALMERIA 1 Mar 2004 EE1002
Birth ALFONSO
LUGO 20 Feb 2013 UNK
Transfer

1003 M 1 Mar 2004 594 609 ALMERIA 1 Mar 2004 EE1003
Birth CHUSPU
29 Jul 2008
Death
[Death by: Injury from exhibit mate + Incinerate + Musculoskeletal + Trauma]

1004 M 1 Mar 2004 594 609 ALMERIA 1 Mar 2004 EE1004
Birth ALVARO
22 Apr 2008
Death
[Death by: Other/Unknown + Incinerate + Urinary + Metabolism]

1005 M 15 Mar 2004 594 539 ALMERIA 15 Mar 2004 EE1005
Birth
20 Mar 2004
Death
[Death by: Unknown means]

1006 F 15 Mar 2004 594 539 ALMERIA 15 Mar 2004 EE1006
Birth laura
29 Mar 2004
Death
[Death by: Unknown means]

1007 F 16 Mar 2004 583 577 ALMERIA 16 Mar 2004 EE1007
Birth VIDEO
16 Mar 2004
Death
[Death by: Unknown means]

1008 F 21 Mar 2004 584 364 ALMERIA 21 Mar 2004 EE1008
Birth LEIRE

You created this PDF from an application that is not licensed to print to novaPDF printer (http://www.novapdf.com)

30 Mar 2004
Death
[Death by: Unknown means]

1009 M 9 Apr 2004 585 633 ALMERIA 9 Apr 2004 EE1009
Birth
18 Apr 2004
Death
[Death by: Unknown means]

1010 M 27 Apr 2004 546 623 ALMERIA 27 Apr 2004 EE1010
Birth
19 Sep 2006
Death
[Death by: Unknown means]

1011 F 30 Apr 2004 584 647 ALMERIA 30 Apr 2004 EE1011
Birth
3 May 2004
Death
[Death by: Unknown means]

1012 F 7 May 2004 546 560 ALMERIA 7 May 2004 EE1012
Birth
LA LAJITA 27 Nov 2006 LA1012
Transfer

1013 F 7 May 2004 546 566 ALMERIA 7 May 2004 EE1013
Birth
LA LAJITA 27 Nov 2006 LA1013
Transfer

1014 M 18 Feb 2003 814 732 SANDIEGOZ 18 Feb 2003 503041
Birth
FOUTS J 28 Aug 2007 UNK
Transfer

1015 M 18 Feb 2003 814 732 SANDIEGOZ 18 Feb 2003 503040
Birth
18 Feb 2003
Death
[Death by: Unknown means]

1016 M 1 Mar 2003 700 951 SANDIEGOZ 1 Mar 2003 503046
Birth
OR WILDLF 9 Nov 2004 203919
Transfer
26 Mar 2006
Death
[Death by: Injury from exhibit mate + Rendered +
Generalized + Trauma]

1017 F 1 Mar 2003 700 951 SANDIEGOZ 1 Mar 2003 503047
Birth
3 Mar 2003
Death
[Death by: Unknown means]

1018 F 10 Mar 2003 825 861 PEACE RV 10 Mar 2003 203020
Birth

1019 F 20 Mar 2003 700 857 SANDIEGOZ 20 Mar 2003 503066
Birth
PEACE RV 19 Nov 2004 UNK
Transfer

1020 F 24 Mar 2003 820 923 LANGLEY 24 Mar 2003 H03006
Birth CLEMENTINE
14 May 2003

You created this PDF from an application that is not licensed to print to novaPDF printer (http://www.novapdf.com)

Death
[Death by: Unknown means]

1021 F 25 Mar 2003 825 872 PEACE RV 25 Mar 2003 203021
Birth

1022 F 25 Mar 2003 820 832 LANGLEY 25 Mar 2003 H03007
Birth C.C.
15 Jul 2005
Death
[Death by: Unknown means]

1023 F 26 Mar 2003 814 813 SANDIEGOZ 26 Mar 2003 503067
Birth
15 Apr 2003
Death
[Death by: Unknown means]

1024 F 28 Mar 2003 814 815 SANDIEGOZ 28 Mar 2003 503068
Birth
28 Mar 2003
Death
[Death by: Unknown means]

1025 M 29 Mar 2003 778 774 PALM DES 29 Mar 2003 403004
Birth FRANCOIS

1026 M 31 Mar 2003 822 760 ST LOUIS 31 Mar 2003 102355
Birth
31 Mar 2003
Death
[Death by: Unknown means]

1027 F 6 Apr 2003 814 848 SANDIEGOZ 6 Apr 2003 503078
Birth
23 Oct 2004
Death
[Death by: Unknown means]

1028 M 9 Apr 2003 814 856 SANDIEGOZ 9 Apr 2003 503082
Birth

1029 F 11 Apr 2003 904 663 LANGLEY 11 Apr 2003 H03010
Birth CAMUS
20 Mar 2005
Death
[Death by: Unknown means]

1030 M 10 May 2003 838 876 LANGLEY 10 May 2003 H03014
Birth CARY
31 Oct 2004
Death
[Death by: Unknown means]

1031 F 10 May 2003 838 876 LANGLEY 10 May 2003 H03015
Birth
12 May 2003
Death
[Death by: Unknown means]

1032 M 12 May 2003 904 882 LANGLEY 12 May 2003 H03016
Birth COSMOS
11 Mar 2004
Death
[Death by: Injury from exhibit mate + Unknown + No necropsy planned]

1033 F 12 May 2003 904 882 LANGLEY 12 May 2003 H03017
Birth CAMILLA

You created this PDF from an application that is not licensed to print to novaPDF printer (http://www.novapdf.com)

```
                                                            13 Jul 2004
Death
                              [Death by: Unknown means]

      1034   M    18 May 2003     838     944  LANGLEY    18 May 2003 H03018
Birth     CISCO
                                                             7 May 2005
Death
                              [Death by: Unknown means]

      1035   M    18 May 2003     838     944  LANGLEY    18 May 2003 H03019
Birth     CLARK
                                                             8 Jun 2003
Death
                              [Death by: Unknown means]

      1036   M    19 May 2003     814     858  SANDIEGOZ  19 May 2003 503163
Birth
                                                            22 May 2003
Death
                              [Death by: Unknown means]

      1037   F    22 Jun 2003     814     706  SANDIEGOZ  22 Jun 2003 503207
Birth
                                               PEACE RV   19 Nov 2004    UNK
Transfer

      1038   F    22 Jun 2003     814     706  SANDIEGOZ  22 Jun 2003 503208
Birth
                                                          22 Jun 2003
Death
                              [Death by: Unknown means]

      1039   M    24 Jun 2003     838     907  LANGLEY    24 Jun 2003 H03025
Birth
                                                          29 Oct 2004
Death
                              [Death by: Unknown means]

      1040   F    24 Jun 2003     838     907  LANGLEY    24 Jun 2003 H03026
Birth
                                               UARAB EM   24 Apr 2005    UNK
ltf

      1041   ?     8 Nov 2003     904     663  LANGLEY     8 Nov 2003 H03037
Birth
                                                           9 Nov 2003
Death
                              [Death by: Unknown means]

      1042   F     4 Jun 2004     565     321  ALMERIA     4 Jun 2004 EE1042
Birth
                                                          16 Jun 2004
Death
                              [Death by: Unknown means]

      1043   F     5 Jun 2004     584     971  ALMERIA     5 Jun 2004 EE1043
Birth
                                                          16 Jul 2004
Death
                              [Death by: Unknown means]

      1044   F    28 Jun 2004     584     966  ALMERIA    28 Jun 2004 EE1044
Birth
                                                          25 Apr 2013
Death
                              [Death by: Unknown means]

      1045   M    30 Jun 2004     584     592  ALMERIA    30 Jun 2004 EE1045
```


You created this PDF from an application that is not licensed to print to novaPDF printer (http://www.novapdf.com)

Birth
6 Jul 2004
Death
[Death by: Unknown means]

1046 F 30 Jun 2004 584 592 ALMERIA 30 Jun 2004 EE1046
Birth

1047 F 30 Jul 2004 584 591 ALMERIA 30 Jul 2004 EE1047
Birth
10 Sep 2004
Death
[Death by: Unknown means]

1048 M 10 Aug 2004 565 385 ALMERIA 10 Aug 2004 EE1048
Birth
22 Sep 2006
Death
[Death by: Injury from predator | Incinerate | No necropsy planned]

1049 F 10 Aug 2004 565 385 ALMERIA 10 Aug 2004 EE1049
Birth

1050 F 16 Sep 2004 594 555 ALMERIA 16 Sep 2004 EE1050
Birth
3 Oct 2012
Death
[Death by: Unknown means]

1051 F 16 Sep 2004 594 555 ALMERIA 16 Sep 2004 EE1051
Birth

1052 M 17 Sep 2004 594 588 ALMERIA 17 Sep 2004 EE1052
Birth

1053 F 17 Sep 2004 594 588 ALMERIA 17 Sep 2004 EE1053
Birth PILAR
19 Nov 2011
Death
[Death by: Unknown means]

1054 M 2 Oct 2004 594 608 ALMERIA 2 Oct 2004 EE1054
Birth
16 Jan 2012
Death
[Death by: Unknown means]

1055 M 19 Oct 2004 624 632 ALMERIA 19 Oct 2004 EE1055
Birth CANO
18 Sep 2005
Death
[Death by: Unknown means]

1056 M 4 Nov 2004 583 572 ALMERIA 4 Nov 2004 EE1056
Birth
6 Nov 2004
Death
[Death by: Unknown means]

1057 M 25 Feb 2005 583 577 ALMERIA 25 Feb 2005 EE1057
Birth
28 Feb 2005
Death
[Death by: Unknown means]

1058 M 4 Mar 2005 624 641 ALMERIA 4 Mar 2005 EE1058
Birth
20 Mar 2005

You created this PDF from an application that is not licensed to print to novaPDF printer (http://www.novapdf.com)

```
Death
                            [Death by: Unknown means]

      1059   F     4 Mar 2005     624     641  ALMERIA     4 Mar 2005 EE1059
Birth
                                                          20 Mar 2005
Death
                            [Death by: Unknown means]

      1060   F    20 Mar 2005     524     533  ALMERIA    20 Mar 2005 EE1060
Birth
                                                           2 Apr 2005
Death
                            [Death by: Unknown means]

      1061   M    20 Mar 2005     524     533  ALMERIA    20 Mar 2005 EE1061
Birth
                                                          25 Mar 2005
Death
                            [Death by: Unknown means]

      1062   F     1 Apr 2005     524     570  ALMERIA     1 Apr 2005 EE1062
Birth     FRIDA
                                               LA LAJITA  27 Nov 2006 LA1062
Transfer

      1063   F     3 Apr 2005     524     578  ALMERIA     3 Apr 2005 EE1063
Birth
                                               LA LAJITA  27 Nov 2006     H2
Transfer
                                                           6 Jun 2011
Death
                            [Death by: Unknown means]

      1064   M    29 Apr 2005     524     553  ALMERIA    29 Apr 2005 EE1064
Birth     ATILA
                                               ESTEPONA   13 Jul 2010    UNK
Transfer

      1065   F    20 May 2005     612     999  ALMERIA    20 May 2005 EE1065
Birth
                                                          24 Feb 2006
Death
                            [Death by: Infection associated + Incinerate +
Generalized + Bacterial]

      1066   M    27 May 2005     580     647  ALMERIA    27 May 2005 EE1066
Birth     CESAR

      1067   M    31 May 2005     629     991  ALMERIA    31 May 2005 EE1067
Birth     PILARICO
                                                           3 Dec 2007
Death
                            [Death by: Injury from exhibit mate + Incinerate +
Generalized + Trauma]

      1068   F     2 Jun 2005     580     966  ALMERIA     2 Jun 2005 EE1068
Birth
                                                          22 Sep 2006
Death
                            [Death by: Injury from predator + Incinerate + No
necropsy planned]

      1069   F     5 Jun 2005     635     588  ALMERIA     5 Jun 2005 EE1069
Birth
                                                          20 Aug 2011
Death
                            [Death by: Unknown means]
```


You created this PDF from an application that is not licensed to print to novaPDF printer (http://www.novapdf.com)

Birth	1070	F	5 Jun 2005	635	588	ALMERIA	5 Jun 2005	EE1070
Birth	1071 AMADOR	M	8 Jun 2005	612	996	ALMERIA	8 Jun 2005	EE1071
Death							14 Jun 2005	
			[Death by: Unknown means]					
Birth	1072	F	8 Jun 2005	635	555	ALMERIA	8 Jun 2005	EE1072
Birth	1073	F	8 Jun 2005	635	555	ALMERIA	8 Jun 2005	EE1073
Death							10 Jul 2006	
			[Death by: Unknown means]					
Birth	1074	F	10 Jun 2005	612	995	ALMERIA	10 Jun 2005	EE1074
Death							16 Jun 2005	
			[Death by: Unknown means]					
Birth	1075	M	10 Jun 2005	635	609	ALMERIA	10 Jun 2005	EE1075
Death							22 Aug 2005	
			[Death by: Unknown means]					
Birth	1076	M	10 Jun 2005	635	609	ALMERIA	10 Jun 2005	EE1076
Death							18 Dec 2008	
			[Death by: Unknown means]					
Birth	1077	M	24 Mar 2004	700	857	SANDIEGOZ	24 Mar 2004	504038
Death							24 Mar 2004	
			[Death by: Unknown means]					
Birth	1078	F	24 Mar 2004	700	857	SANDIEGOZ	24 Mar 2004	504039
ltf						JOHNSONLA	20 Nov 2006	UNK
Birth	1079	F	1 Apr 2004	700	951	SANDIEGOZ	1 Apr 2004	504045
Birth	1080	F	3 Apr 2004	904	985	LANGLEY	3 Apr 2004	H04004
ltf						UARAB EM	24 Apr 2005	UNK
Birth	1081	M	3 Apr 2004	904	985	LANGLEY	3 Apr 2004	H04005
Death							4 Apr 2004	
			[Death by: Unknown means]					
Birth	1082	M	6 Apr 2004	976	882	LANGLEY	6 Apr 2004	H04006
ltf						UARAB EM	24 Apr 2005	UNK
Birth	1083	F	6 Apr 2004	976	882	LANGLEY	6 Apr 2004	H04007
						UARAB EM	24 Apr 2005	UNK

You created this PDF from an application that is not licensed to print to novaPDF printer (http://www.novapdf.com)

ltf

1084 M 23 Apr 2004 904 923 LANGLEY 23 Apr 2004 H04011
Birth
24 Apr 2004
Death
[Death by: Unknown means]

1085 F 23 Apr 2004 904 923 LANGLEY 23 Apr 2004 H04012
Birth
UARAB EM 24 Apr 2005 UNK
ltf

1086 F 10 May 2004 820 950 LANGLEY 10 May 2004 H04013
Birth

1087 F 10 May 2004 820 950 LANGLEY 10 May 2004 H04014
Birth
UARAB EM 24 Apr 2005 UNK
ltf

1088 ? 11 May 2004 820 763 LANGLEY 11 May 2004 H04016
Birth
11 May 2004
Death
[Death by: Unknown means]

1089 F 27 May 2004 822 760 ST LOUIS 27 May 2004 103491
Birth
SANDIEGOZ 27 Feb 2005 605040
Transfer
JOHNSONLA 14 May 2007 UNK
Transfer

1090 F 6 Aug 2005 580 592 ALMERIA 6 Aug 2005 EE1090
Birth
25 Jan 2006
Death
[Death by: Injury from exhibit mate + Incinerate +
Generalized + Trauma]

1091 F 24 Sep 2005 583 577 ALMERIA 24 Sep 2005 EE1091
Birth

1092 F 11 Sep 2005 624 641 ALMERIA 11 Sep 2005 EE1092
Birth

1093 M 9 Oct 2005 524 533 ALMERIA 9 Oct 2005 EE1093
Birth
13 Jul 2006
Death
[Death by: Infection associated + Incinerate +
Generalized + Bacterial]

1094 ? 27 May 2004 976 663 LANGLEY 27 May 2004 H04018
Birth
27 May 2004
Death
[Death by: Unknown means]

1095 F 29 Jun 2004 893 928 OR WILDLF 29 Jun 2004 204907
Birth
29 Jun 2004
Death
[Death by: Unknown means]

1096 F 29 Jun 2004 893 928 OR WILDLF 29 Jun 2004 204908
Birth
29 Jun 2004

You created this PDF from an application that is not licensed to print to novaPDF printer (http://www.novapdf.com)

Death
[Death by: Unknown means]

1097 M 29 Jun 2004 893 928 OR WILDLF 29 Jun 2004 204909
Birth
29 Jun 2004
Death
[Death by: Unknown means]

1098 F 30 Jun 2004 893 898 OR WILDLF 30 Jun 2004 204893
Birth
27 Jun 2006
Death
[Death by: Unknown means]

1099 M 30 Jun 2004 893 898 OR WILDLF 30 Jun 2004 204894
Birth
19 Dec 2006
Death
[Death by: Other/Unknown + Incinerate + Generalized +
Trauma]

1100 F 2 Jul 2004 893 953 OR WILDLF 2 Jul 2004 204895
Birth

1101 F 2 Jul 2004 893 953 OR WILDLF 2 Jul 2004 204896
Birth
31 Dec 2007
Death
[Death by: Unknown means]

1102 F 4 Jul 2004 700 706 SANDIEGOZ 4 Jul 2004 504163
Birth

1103 M 7 Jul 2004 893 802 OR WILDLF 7 Jul 2004 204899
Birth

1104 ? 8 Jul 2004 893 804 OR WILDLF 8 Jul 2004 204910
Birth
8 Jul 2004
Death
[Death by: Unknown means]

1105 ? 8 Jul 2004 893 804 OR WILDLF 8 Jul 2004 204911
Birth
8 Jul 2004
Death
[Death by: Unknown means]

1106 M 14 Jul 2004 700 856 SANDIEGOZ 14 Jul 2004 504171
Birth
PEACE RV 19 Nov 2004 UNK
Transfer

1107 M 24 Jul 2004 893 902 OR WILDLF 24 Jul 2004 204900
Birth
18 Dec 2007
Death
[Death by: Unknown means]

1108 M 24 Jul 2004 893 902 OR WILDLF 24 Jul 2004 204901
Birth
9 Sep 2004
Death
[Death by: Unknown means]

1109 F 6 Aug 2004 700 813 SANDIEGOZ 6 Aug 2004 504181
Birth
PEACE RV 19 Nov 2004 UNK

You created this PDF from an application that is not licensed to print to novaPDF printer (http://www.novapdf.com)

```
Transfer
        1110    F     6 Aug 2004     700     813   SANDIEGOZ    6 Aug 2004 504182
Birth
                                                   PEACE RV    19 Nov 2004    UNK
Transfer
        1111    F    12 Aug 2004     700     858   SANDIEGOZ   12 Aug 2004 504184
Birth
                                                   PEACE RV    19 Nov 2004    UNK
Transfer
        1112    M    25 Oct 2004     838     876   LANGLEY     25 Oct 2004    UNK
Birth
                                                               25 Oct 2004
Death
                           [Death by: Unknown means]
        1113    F     8 Dec 2004     904     985   LANGLEY      8 Dec 2004    UNK
Birth
                                                                8 Dec 2004
Death
                           [Death by: Unknown means]
        1114    M    15 Dec 2004     976     663   LANGLEY     15 Dec 2004    UNK
Birth
                                                               16 Dec 2004
Death
                           [Death by: Unknown means]
        1115    M    27 Feb 2005     700     706   SANDIEGOZ   27 Feb 2005 505028
Birth
                                                   JOHNSONLA   14 May 2007    UNK
ltf
        1116    F    27 Feb 2005     700     706   SANDIEGOZ   27 Feb 2005 505029
Birth
                                                   JOHNSONLA   22 Aug 2007    UNK
ltf
        1117    M     3 Mar 2005     700     813   SANDIEGOZ    3 Mar 2005 505031
Birth
                                                   JOHNSONLA   20 Nov 2006    UNK
ltf
        1118    M     3 Mar 2005     700     813   SANDIEGOZ    3 Mar 2005 505032
Birth
                                                               17 Sep 2006
Death
                           [Death by: Unknown means]
        1119    M     9 Mar 2005     822     760   ST LOUIS     9 Mar 2005 103970
Birth
                                                   OR WILDLF   21 Nov 2005 205952
Transfer
                                                                8 Sep 2006
Death
                           [Death by: Injury from exhibit mate + Incinerate +
Generalized + Trauma]
        1120    F    12 Mar 2005     700     815   SANDIEGOZ   12 Mar 2005 505039
Birth
                                                               13 Mar 2005
Death
                           [Death by: Unknown means]
        1121    F    12 Mar 2005     700     815   SANDIEGOZ   12 Mar 2005 505040
Birth
                                                               14 Mar 2005
```


You created this PDF from an application that is not licensed to print to novaPDF printer (http://www.novapdf.com)

Death
[Death by: Unknown means]

1122 F 26 Mar 2005 700 858 SANDIEGOZ 26 Mar 2005 505047
Birth
JOHNSONLA 27 May 2007 UNK
ltf

1123 M 27 Mar 2005 820 950 LANGLEY 27 Mar 2005 UNK
Birth
15 Jan 2006
Death
[Death by: Injury from exhibit mate + Unknown + Unknown (after necropsy)]

1124 F 21 Apr 2005 904 1022 LANGLEY 21 Apr 2005 UNK
Birth

1125 F 21 Apr 2005 904 1022 LANGLEY 21 Apr 2005 UNK
Birth
10 Nov 2007
Death
[Death by: Unknown means]

1126 F 23 May 2005 838 876 LANGLEY 23 May 2005 UNK
Birth
4 Oct 2006
Death
[Death by: Injury from exhibit mate + Unknown + Unknown (after necropsy) + Metazoan]

1127 M 23 May 2005 838 876 LANGLEY 23 May 2005 UNK
Birth
23 May 2005
Death
[Death by: Unknown means]

1128 M 9 Feb 2006 580 647 ALMERIA 9 Feb 2006 EE1128
Birth

1129 M 11 Feb 2006 580 966 ALMERIA 11 Feb 2006 EE1129
Birth
14 Aug 2006
Death
[Death by: Unknown means]

1130 F 11 Feb 2006 580 966 ALMERIA 11 Feb 2006 EE1130
Birth

1131 F 6 Mar 2006 580 592 ALMERIA 6 Mar 2006 EE1131
Birth
25 Apr 2006
Death
[Death by: Unknown means]

1132 F 6 Mar 2006 580 592 ALMERIA 6 Mar 2006 EE1132
Birth
10 Apr 2006
Death
[Death by: Unknown means]

1133 F 10 Mar 2006 583 577 ALMERIA 10 Mar 2006 EE1133
Birth
14 May 2010
Death
[Death by: Unknown means]

1134 F 10 Mar 2006 583 577 ALMERIA 10 Mar 2006 EE1134
Birth

You created this PDF from an application that is not licensed to print to novaPDF printer (http://www.novapdf.com)

1135 M 11 Mar 2006 612 1046 ALMERIA 11 Mar 2006 EE1135
Birth

1136 F 11 Mar 2006 612 1046 ALMERIA 11 Mar 2006 EE1136
Birth
25 Apr 2009
Death
[Death by: Unknown means]

1137 F 14 Mar 2006 546 972 ALMERIA 14 Mar 2006 EE1137
Birth
22 Sep 2006
Death
[Death by: Injury from predator | Incinerate | No necropsy planned]

1138 F 21 Mar 2006 624 641 ALMERIA 21 Mar 2006 EE1138
Birth

1139 F 21 Mar 2006 624 641 ALMERIA 21 Mar 2006 EE1139
Birth

1140 F 23 Mar 2006 612 1044 ALMERIA 23 Mar 2006 EE1140
Birth

1141 F 23 Mar 2006 612 1044 ALMERIA 23 Mar 2006 EE1141
Birth

1142 F 23 Mar 2006 612 999 ALMERIA 23 Mar 2006 EE1142
Birth

1143 F 23 Mar 2006 612 999 ALMERIA 23 Mar 2006 EE1143
Birth

1144 M 6 Apr 2006 546 1013 ALMERIA 6 Apr 2006 EE1144
Birth
16 May 2006
Death
[Death by: Infection associated | Incinerate | Generalized | Bacterial]

1145 F 12 Apr 2006 546 1012 ALMERIA 12 Apr 2006 EE1145
Birth

1146 M 12 Apr 2006 612 995 ALMERIA 12 Apr 2006 EE1146
Birth
LUGO 20 Feb 2013 UNK
Transfer

1147 M 30 May 2006 540 1050 ALMERIA 30 May 2006 EE1147
Birth
6 Dec 2008
Death
[Death by: Unknown means]

1148 M 2 Jun 2006 549 620 ALMERIA 2 Jun 2006 EE1148
Birth
8 Sep 2006
Death
[Death by: Unknown means]

1149 F 2 Jun 2006 546 1063 ALMERIA 2 Jun 2006 EE1149
Birth

1150 M 5 Jun 2006 540 1053 ALMERIA 5 Jun 2006 EE1150
Birth
2 May 2012
Death

You created this PDF from an application that is not licensed to print to novaPDF printer (http://www.novapdf.com)

[Death by: Unknown means]

1151 M 6 Jun 2006 546 578 ALMERIA 6 Jun 2006 EE1151
Birth
9 Jun 2006
Death
[Death by: Unknown means]

1152 F 6 Jun 2006 546 578 ALMERIA 6 Jun 2006 EE1152
Birth
8 Jun 2006
Death
[Death by: Unknown means]

1153 M 6 Jun 2006 540 609 ALMERIA 6 Jun 2006 EE1153
Birth
11 Mar 2009
Death
[Death by: Unknown means]

1154 F 6 Jun 2006 540 609 ALMERIA 6 Jun 2006 EE1154
Birth
10 Jan 2009
Death
[Death by: Unknown means]

1155 F 9 Jun 2006 540 1051 ALMERIA 9 Jun 2006 EE1155
Birth
30 May 2011
Death
[Death by: Unknown means]

1156 M 12 Jun 2006 540 608 ALMERIA 12 Jun 2006 EE1156
Birth
22 Jun 2006
Death
[Death by: Unknown means]

1157 F 13 Jun 2006 546 533 ALMERIA 13 Jun 2006 EE1157
Birth
15 Jun 2006
Death
[Death by: Unknown means]

1158 M 13 Jun 2006 546 533 ALMERIA 13 Jun 2006 EE1158
Birth
29 Dec 2009
Death
[Death by: Unknown means]

1159 F 14 Jun 2006 549 1049 ALMERIA 14 Jun 2006 EE1159
Birth

1160 M 14 Jun 2006 549 581 ALMERIA 14 Jun 2006 EE1160
Birth

1161 F 14 Jun 2006 549 581 ALMERIA 14 Jun 2006 EE1161
Birth
16 Jan 2008
Death
[Death by: Other/Unknown + Incinerate + Urinary +
Toxicity]

1162 M 17 Jun 2006 546 570 ALMERIA 17 Jun 2006 EE1162
Birth
31 Aug 2006
Death
[Death by: Unknown means]

You created this PDF from an application that is not licensed to print to novaPDF printer (http://www.novapdf.com)

1163 F 17 Jun 2006 546 570 ALMERIA 17 Jun 2006 EE1163
Birth

1164 M 17 Jun 2006 549 991 ALMERIA 17 Jun 2006 EE1164
Birth
21 Oct 2007
Death
[Death by: Injury from exhibit mate + Incinerate +
Generalized + Trauma]

1165 M 1 Jul 2006 549 385 ALMERIA 1 Jul 2006 EE1165
Birth
8 Aug 2007
Death
[Death by: Self-inflicted injuries + Incinerate +
Generalized + Trauma]

1166 F 25 Sep 2006 540 588 ALMERIA 25 Sep 2006 EE1166
Birth
10 Oct 2006
Death
[Death by: Unknown means]

1167 F 2 Oct 2006 583 572 ALMERIA 2 Oct 2006 EE1167
Birth
5 Oct 2006
Death
[Death by: Unknown means]

1168 M 19 Oct 2006 583 577 ALMERIA 19 Oct 2006 EE1168
Birth
13 Jul 2012
Death
[Death by: Unknown means]

1169 F 29 Oct 2006 624 641 ALMERIA 29 Oct 2006 EE1169
Birth
17 Aug 2011
Death
[Death by: Unknown means]

1170 M 18 Jan 2007 549 620 ALMERIA 18 Jan 2007 EE1170
Birth
ESTEPONA 19 Dec 2012 UNK
Transfer

1171 M 18 Jan 2007 549 620 ALMERIA 18 Jan 2007 EE1171
Birth

1172 M 27 Jan 2007 549 991 ALMERIA 27 Jan 2007 EE1172
Birth
11 Apr 2012
Death
[Death by: Unknown means]

1173 M 30 Jan 2007 549 581 ALMERIA 30 Jan 2007 EE1173
Birth ALFONSO III
10 Jan 2012
Death
[Death by: Unknown means]

1174 F 17 Mar 2007 549 1049 ALMERIA 17 Mar 2007 EE1174
Birth

1175 M 19 Mar 2007 580 592 ALMERIA 19 Mar 2007 EE1175
Birth
9 Sep 2011
Death
[Death by: Unknown means]

You created this PDF from an application that is not licensed to print to novaPDF printer (http://www.novapdf.com)

1176 M 19 Mar 2007 580 592 ALMERIA 19 Mar 2007 EE1176
Birth
ESTEPONA 11 Dec 2007 1GCU1
Transfer

1177 F 24 Mar 2007 624 1092 ALMERIA 24 Mar 2007 EE1177
Birth

1178 F 5 Apr 2007 583 1134 ALMERIA 5 Apr 2007 EE1178
Birth

1179 M 13 Apr 2007 583 1133 ALMERIA 13 Apr 2007 EE1179
Birth
ESTEPONA 11 Dec 2007 1GCU2
Transfer

1180 M 13 Apr 2007 580 1130 ALMERIA 13 Apr 2007 EE1180
Birth
ESTEPONA 11 Dec 2007 1GCU3
Transfer
30 Nov 2008
Death
[Death by: Unknown means]

1181 F 23 Apr 2007 624 1139 ALMERIA 23 Apr 2007 EE1181
Birth
24 Apr 2007
Death
[Death by: Stillbirth | Incinerate | Generalized |
Nutrition]

1182 F 23 Apr 2007 624 1138 ALMERIA 23 Apr 2007 EE1182
Birth

1183 F 30 Apr 2007 624 641 ALMERIA 30 Apr 2007 EE1183
Birth

1184 M 30 Apr 2007 624 641 ALMERIA 30 Apr 2007 EE1184
Birth
ESTEPONA 11 Dec 2007 1GCU4
Transfer

1185 M 6 May 2007 624 640 ALMERIA 6 May 2007 EE1185
Birth
ESTEPONA 11 Dec 2007 1GCU5
Transfer
4 Feb 2008
Death
[Death by: Unknown means]

1186 F 6 May 2007 624 640 ALMERIA 6 May 2007 EE1186
Birth

1187 M 27 May 2007 583 577 ALMERIA 27 May 2007 EE1187
Birth
4 Dec 2012
Death
[Death by: Unknown means]

1188 M 27 May 2007 583 577 ALMERIA 27 May 2007 EE1188
Birth
28 Jun 2012
Death
[Death by: Unknown means]

1189 F 24 Jun 2007 583 572 ALMERIA 24 Jun 2007 EE1189
Birth
30 Jun 2007

You created this PDF from an application that is not licensed to print to novaPDF printer (http://www.novapdf.com)

Death

[Death by: Stillbirth + Incinerate + Generalized + Nutrition]

1190 F 6 Mar 2007 650 1063 LA LAJITA 6 Mar 2007 UNK
Birth

1191 M 21 May 2007 650 1012 LA LAJITA 21 May 2007 UNK
Birth

1192 F 22 May 2007 650 647 LA LAJITA 22 May 2007 UNK
Birth

1193 F 30 May 2007 650 1013 LA LAJITA 30 May 2007 UNK
Birth

1194 F 30 May 2007 650 1013 LA LAJITA 30 May 2007 UNK
Birth
4 Mar 2011
Death
[Death by: Unknown means]

1195 M 6 Jun 2007 650 1062 LA LAJITA 6 Jun 2007 UNK
Birth

1196 F 4 Jun 2005 1028 951 SANDIEGOZ 4 Jun 2005 505141
Birth
JOHNSONLA 14 May 2007 UNK
ltf

1197 F 5 Jun 2005 904 957 LANGLEY 5 Jun 2005 H05018
Birth
17 Jun 2005
Death
[Death by: Unknown means]

1198 F 9 Oct 2005 1028 858 SANDIEGOZ 9 Oct 2005 505233
Birth
JOHNSONLA 14 May 2007 UNK
ltf

1199 M 27 Oct 2005 1028 706 SANDIEGOZ 27 Oct 2005 505247
Birth
29 Oct 2005
Death
[Death by: Unknown means]

1200 M 1 Jan 2006 976 985 LANGLEY 1 Jan 2006 H06001
Birth
3 Jan 2006
Death
[Death by: Unknown means]

1201 M 4 Mar 2006 1028 813 SANDIEGOZ 4 Mar 2006 506034
Birth

1202 M 4 Mar 2006 1028 813 SANDIEGOZ 4 Mar 2006 506035
Birth
JOHNSONLA 20 Nov 2006 UNK
ltf

1203 M 28 Mar 2006 1028 1078 SANDIEGOZ 28 Mar 2006 506099
Birth
JOHNSONLA 20 Nov 2006 UNK
ltf

1204 M 24 Apr 2006 976 876 LANGLEY 24 Apr 2006 H06024
Birth
24 Apr 2006

You created this PDF from an application that is not licensed to print to novaPDF printer (http://www.novapdf.com)

Death
[Death by: Unknown means]

1205 F 24 Apr 2006 976 876 LANGLEY 24 Apr 2006 H06025
Birth
24 Apr 2006
Death
[Death by: Unknown means]

1206 M 30 Apr 2006 976 1086 LANGLEY 30 Apr 2006 H06029
Birth
30 Apr 2006
Death
[Death by: Unknown means]

1207 M 12 May 2006 1028 706 SANDIEGOZ 12 May 2006 506139
Birth
JOHNSONLA 22 Aug 2007 UNK
ltf

1208 F 19 May 2006 989 1089 SANDIEGOZ 19 May 2006 506147
Birth
JOHNSONLA 14 May 2007 UNK
ltf

1209 F 24 May 2006 1016 953 OR WILDLF 24 May 2006 206959
Birth

1210 M 26 May 2006 1016 898 OR WILDLF 26 May 2006 206960
Birth

1211 F 26 May 2006 1016 898 OR WILDLF 26 May 2006 206964
Birth
18 Apr 2007
Death
[Death by: Unknown means]

1212 M 3 Jun 2006 1016 928 OR WILDLF 3 Jun 2006 206961
Birth

1213 M 5 Jun 2006 1016 796 OR WILDLF 5 Jun 2006 206963
Birth
16 Jul 2006
Death
[Death by: Unknown means]

1214 M 9 Jun 2006 1028 1079 SANDIEGOZ 9 Jun 2006 506186
Birth
JOHNSONLA 22 Aug 2007 UNK
ltf

1215 F 10 Jun 2006 1016 802 OR WILDLF 10 Jun 2006 206962
Birth

1216 F 10 Jun 2006 1016 1101 OR WILDLF 10 Jun 2006 206965
Birth

1217 F 19 Jun 2006 1028 858 SANDIEGOZ 19 Jun 2006 506204
Birth

1218 F 26 Jun 2006 976 985 LANGLEY 26 Jun 2006 H06036
Birth
26 Jun 2006
Death
[Death by: Unknown means]

1219 F 26 Jun 2006 976 985 LANGLEY 26 Jun 2006 H06037
Birth
27 Jun 2006

You created this PDF from an application that is not licensed to print to novaPDF printer (http://www.novapdf.com)

Death
[Death by: Unknown means]

1220 M 19 Feb 2007 1028 813 SANDIEGOZ 19 Feb 2007 507037
Birth

1221 M 19 Feb 2007 1028 813 SANDIEGOZ 19 Feb 2007 507036
Birth
28 Feb 2007
Death
[Death by: Unknown means]

1222 F 24 Feb 2007 1028 706 SANDIEGOZ 24 Feb 2007 507053
Birth
FOUTS J 28 Aug 2007 UNK
Transfer

1223 M 28 Jul 2007 549 620 ALMERIA 28 Jul 2007 EE1223
Birth
ESTEPONA 19 Dec 2012 UNK
Transfer

1224 F 28 Jul 2007 549 620 ALMERIA 28 Jul 2007 EE1224
Birth
20 Dec 2010
Death
[Death by: Unknown means]

1225 M 13 Aug 2007 549 581 ALMERIA 13 Aug 2007 EE1225
Birth SILVIO
20 Feb 2009
Death
[Death by: Unknown means]

1226 F 18 Aug 2007 549 991 ALMERIA 18 Aug 2007 EE1226
Birth
13 Sep 2007
Death
[Death by: Infection associated + Incinerate +
Generalized + Bacterial]

1227 F 12 Oct 2007 645 1044 ALMERIA 12 Oct 2007 EE1227
Birth

1228 M 18 Oct 2007 646 1051 ALMERIA 18 Oct 2007 EE1228
Birth
25 Nov 2012
Death
[Death by: Unknown means]

1229 F 19 Oct 2007 580 570 ALMERIA 19 Oct 2007 EE1229
Birth
25 Oct 2007
Death
[Death by: Stillbirth + Incinerate + Generalized +
Nutrition]

1230 F 20 Oct 2007 645 995 ALMERIA 20 Oct 2007 EE1230
Birth

1231 M 21 Oct 2007 646 588 ALMERIA 21 Oct 2007 EE1231
Birth

1232 M 25 Oct 2007 645 1046 ALMERIA 25 Oct 2007 EE1232
Birth
LUGO 20 Feb 2013 UNK
Transfer

1233 M 25 Oct 2007 646 609 ALMERIA 25 Oct 2007 EE1233

You created this PDF from an application that is not licensed to print to novaPDF printer (http://www.novapdf.com)

Birth

1234 M 25 Oct 2007 646 609 ALMERIA 25 Oct 2007 EE1234
Birth

1235 F 26 Oct 2007 646 1050 ALMERIA 26 Oct 2007 EE1235
Birth

1236 M 28 Oct 2007 646 1053 ALMERIA 28 Oct 2007 EE1236
Birth
29 Apr 2008
Death
[Death by: Unknown means]

1237 M 29 Oct 2007 642 1049 ALMERIA 29 Oct 2007 EE1237
Birth

1238 M 30 Oct 2007 645 996 ALMERIA 30 Oct 2007 EE1238
Birth
1 Nov 2007
Death
[Death by: Unknown means]

1239 F 20 Nov 2007 648 533 ALMERIA 20 Nov 2007 EE1239
Birth

1240 F 11 Oct 2007 650 1063 LA LAJITA 11 Oct 2007 UNK
Birth

1241 M 7 May 1981 UNK UNK MUNICH 7 May 1981 9
Birth
L RUHE 25 Jan 1983 UNK
ltf

1242 F 4 Jun 1981 UNK UNK MUNICH 4 Jun 1981 10
Birth
L RUHE 25 Jan 1983 UNK
ltf

1243 F 4 Jun 1981 UNK UNK MUNICH 4 Jun 1981 11
Birth CONCHITA
10 May 1987
Death
[Death by: Unknown means]

1244 M 13 Apr 1984 UNK 22 MUNICH 13 Apr 1984 12
Birth ROMINO
16 Apr 1984
Death
[Death by: Unknown means]

1245 F 14 Mar 1982 UNK 22 MUNICH 14 Mar 1982 13
Birth
L RUHE 25 Jan 1983 UNK
ltf

1246 F 14 Mar 1982 UNK 22 MUNICH 14 Mar 1982 14
Birth
22 Mar 1982
Death
[Death by: Unknown means]

1247 M 25 Mar 1982 UNK 68 MUNICH 25 Mar 1982 15
Birth
L RUHE 25 Jan 1983 UNK
ltf

1248 F 25 Mar 1982 UNK 68 MUNICH 25 Mar 1982 16
Birth MARGARITA

You created this PDF from an application that is not licensed to print to novaPDF printer (http://www.novapdf.com)

9 Nov 1984
Death
[Death by: Unknown means]

1249 M 18 Sep 1982 UNK 66 MUNICH 18 Sep 1982 17
Birth PEDRO
L RUHE 9 Nov 1983 UNK
ltf

1250 M 18 Sep 1982 UNK 66 MUNICH 18 Sep 1982 18
Birth
14 Mar 1983
Death
[Death by: Unknown means]

1251 F 13 Nov 1982 UNK 22 MUNICH 13 Nov 1982 19
Birth ESPERANZA
L RUHE 9 Nov 1983 UNK
ltf

1252 M 15 Nov 1982 UNK UNK MUNICH 15 Nov 1982 20
Birth DIEGO
L RUHE 6 Sep 1983 UNK
ltf

1253 M 18 Mar 1983 UNK 1243 MUNICH 18 Mar 1983 21
Birth
22 Mar 1983
Death
[Death by: Unknown means]

1254 F 18 Mar 1983 UNK 1243 MUNICH 18 Mar 1983 22
Birth
20 Mar 1983
Death
[Death by: Unknown means]

1255 M 22 Apr 1983 UNK 66 MUNICH 22 Apr 1983 23
Birth
14 May 1983
Death
[Death by: Unknown means]

1256 M 22 Apr 1983 UNK 66 MUNICH 22 Apr 1983 24
Birth
23 Apr 1983
Death
[Death by: Unknown means]

1257 M 2 Oct 1984 UNK 68 MUNICH 2 Oct 1984 32
Birth
8 Oct 1984
Death
[Death by: Infection associated + Unknown + Respiratory + Unknown after necropsy]

1258 M 3 Oct 1983 UNK UNK MUNICH 3 Oct 1983 26
Birth JOSE
9 Dec 1983
Death
[Death by: Unknown means]

1259 M 25 Oct 1984 248 1243 MUNICH 25 Oct 1984 27
Birth COSTA
????
Death
[Death by: Unknown means]

1260 M 21 Nov 1984 UNK 22 MUNICH 21 Nov 1984 28

You created this PDF from an application that is not licensed to print to novaPDF printer (http://www.novapdf.com)

Birth RAMON
????
Death
[Death by: Unknown means]

1261 M 2 Jul 1985 248 22 MUNICH 2 Jul 1985 UNK
Birth RODRIGO
5 Apr 1986
Death
[Death by: Unknown means]

1262 M 2 Jul 1985 248 22 MUNICH 2 Jul 1985 UNK
Birth RAMIREZ
28 Jan 1986
Death
[Death by: Unknown means]

1263 F 13 Apr 1984 UNK 22 MUNICH 13 Apr 1984 31
Birth ROMINA
L RUHE 5 Mar 1986 UNK
ltf

1264 M 28 Feb 1986 UNK 22 MUNICH 28 Feb 1986 UNK
Birth GEMELO
3 Mar 1986
Death
[Death by: Unknown means]

1265 F 7 Mar 1986 UNK 68 MUNICH 7 Mar 1986 UNK
Birth
27 Mar 1987
Death
[Death by: Unknown means]

1266 F 7 Mar 1986 UNK 68 MUNICH 7 Mar 1986 UNK
Birth
27 Mar 1987
Death
[Death by: Unknown means]

1267 M 3 Oct 1986 248 22 MUNICH 3 Oct 1986 UNK
Birth ROMAN
L RUHE 5 Jul 1988 UNK
ltf

1268 M 16 Apr 1988 1267 22 MUNICH 16 Apr 1988 UNK
Birth
22 Apr 1988
Death
[Death by: Unknown means]

1269 M 6 Apr 1986 248 1243 MUNICH 6 Apr 1986 UNK
Birth
12 Feb 1987
Death
[Death by: Unknown means]

1270 M 29 Jan 2008 650 1012 LA LAJITA 29 Jan 2008 UNK
Birth
26 Feb 2009
Death
[Death by: Injury from exhibit mate + Incinerate +
Musculoskeletal + Trauma]

1271 M 3 Feb 2008 650 1013 LA LAJITA 3 Feb 2008 UNK
Birth
26 Feb 2009
Death
[Death by: Injury from exhibit mate + Incinerate +

You created this PDF from an application that is not licensed to print to novaPDF printer (http://www.novapdf.com)

Musculoskeletal ┼ Trauma]

1272 M 3 Feb 2008 650 1013 LA LAJITA 3 Feb 2008 UNK
Birth
7 Feb 2009
Death
[Death by: Unknown means]

1273 F 13 Feb 2008 624 640 ALMERIA 13 Feb 2008 EE1273
Birth BELEN
23 May 2011
Death
[Death by: Unknown means]

1274 F 13 Feb 2008 624 640 ALMERIA 13 Feb 2008 EE1274
Birth MARIA

1275 M 19 Feb 2008 611 1136 ALMERIA 19 Feb 2008 EE1275
Birth
CABARCENO 13 Aug 2009 UNK
Transfer
25 Feb 2010
Death
[Death by: Unknown means]

1276 F 19 Feb 2008 611 1136 ALMERIA 19 Feb 2008 EE1276
Birth

1277 F 21 Feb 2008 649 1072 ALMERIA 21 Feb 2008 EE1277
Birth
14 Mar 2013
Death
[Death by: Unknown means]

1278 F 21 Feb 2008 649 1072 ALMERIA 21 Feb 2008 EE1278
Birth

1279 M 23 Feb 2008 624 641 ALMERIA 23 Feb 2008 EE1279
Birth
CABARCENO 13 Aug 2009 UNK
Transfer
15 Jun 2010
Death
[Death by: Unknown means]

1280 F 23 Feb 2008 624 641 ALMERIA 23 Feb 2008 EE1280
Birth

1281 M 29 Feb 2008 648 1149 ALMERIA 29 Feb 2008 EE1281
Birth
4 Mar 2008
Death
[Death by: Infection associated ┼ Incinerate ┼
Digestive ┼ Nutrition]

1282 M 29 Feb 2008 648 1149 ALMERIA 29 Feb 2008 EE1282
Birth
4 Mar 2008
Death
[Death by: Infection associated ┼ Incinerate ┼
Digestive ┼ Nutrition]

1283 M 1 Mar 2008 642 620 ALMERIA 1 Mar 2008 EE1283
Birth
6 Mar 2008
Death
[Death by: Unknown means]

1284 M 1 Mar 2008 642 620 ALMERIA 1 Mar 2008 EE1284

You created this PDF from an application that is not licensed to print to novaPDF printer (http://www.novapdf.com)

Birth
CABARCENO 13 Aug 2009 UNK
Transfer
14 Dec 2009
Death
[Death by: Unknown means]

1285 M 5 Mar 2008 611 1140 ALMERIA 5 Mar 2008 EE1285
Birth
7 Jun 2009
Death
[Death by: Injury from exhibit mate + Incinerate + Generalized + Trauma]

1286 F 5 Mar 2008 611 1140 ALMERIA 5 Mar 2008 EE1286
Birth

1287 M 6 Mar 2008 642 991 ALMERIA 6 Mar 2008 EE1287
Birth
14 Mar 2008
Death
[Death by: Unknown means]

1288 M 6 Mar 2008 642 991 ALMERIA 6 Mar 2008 EE1288
Birth
CABARCENO 13 Aug 2009 UNK
Transfer
10 Jan 2012
Death
[Death by: Unknown means]

1289 M 10 Mar 2008 611 1142 ALMERIA 10 Mar 2008 EE1289
Birth
CABARCENO 13 Aug 2009 UNK
Transfer
6 May 2010
Death
[Death by: Unknown means]

1290 F 10 Mar 2008 611 1142 ALMERIA 10 Mar 2008 EE1290
Birth
11 Mar 2008
Death
[Death by: Unknown means]

1291 F 8 Mar 2008 649 1070 ALMERIA 8 Mar 2008 EE1291
Birth

1292 M 11 Mar 2008 645 999 ALMERIA 11 Mar 2008 EE1292
Birth
CABARCENO 13 Aug 2009 UNK
Transfer
22 Oct 2010
Death
[Death by: Unknown means]

1293 M 12 Mar 2008 649 1141 ALMERIA 12 Mar 2008 EE1293
Birth
CABARCENO 13 Aug 2009 UNK
Transfer
29 Dec 2009
Death
[Death by: Unknown means]

1294 F 12 Mar 2008 649 1141 ALMERIA 12 Mar 2008 EE1294
Birth

1295 M 14 Mar 2008 611 1069 ALMERIA 14 Mar 2008 EE1295
Birth

You created this PDF from an application that is not licensed to print to novaPDF printer (http://www.novapdf.com)

CABARCENO 13 Aug 2009 UNK
Transfer
5 Jan 2010
Death
[Death by: Unknown means]

1296 M 26 Mar 2008 646 1155 ALMERIA 26 Mar 2008 EE1296
Birth
CABARCENO 13 Aug 2009 UNK
Transfer

1297 M 26 Mar 2008 646 1155 ALMERIA 26 Mar 2008 EE1297
Birth
CABARCENO 13 Aug 2009 UNK
Transfer

1298 F 26 Mar 2008 646 1155 ALMERIA 26 Mar 2008 EE1298
Birth

1299 F 28 Mar 2008 648 1163 ALMERIA 28 Mar 2008 EE1299
Birth

1300 F 28 Mar 2008 648 1163 ALMERIA 28 Mar 2008 EE1300
Birth

1301 F 5 Apr 2008 648 1145 ALMERIA 5 Apr 2008 EE1301
Birth

1302 F 5 Apr 2008 648 1145 ALMERIA 5 Apr 2008 EE1302
Birth
24 Jun 2008
Death
[Death by: Unknown means]

1303 M 7 Apr 2008 646 1051 ALMERIA 7 Apr 2008 EE1303
Birth
CABARCENO 13 Aug 2009 UNK
Transfer
11 Nov 2010
Death
[Death by: Unknown means]

1304 F 7 Apr 2008 646 1051 ALMERIA 7 Apr 2008 EE1304
Birth

1305 M 17 Apr 2008 646 1050 ALMERIA 17 Apr 2008 EE1305
Birth
CABARCENO 13 Aug 2009 UNK
Transfer
9 Dec 2009
Death
[Death by: Unknown means]

1306 F 17 Apr 2008 646 1050 ALMERIA 17 Apr 2008 EE1307
Birth

1307 M 21 Apr 2008 645 995 ALMERIA 21 Apr 2008 EE1307
Birth JUAN PEDRO T
CABARCENO 13 Aug 2009 UNK
Transfer

1308 F 21 Apr 2008 645 995 ALMERIA 21 Apr 2008 EE1308
Birth BELEN TV

1309 F 21 Apr 2008 645 995 ALMERIA 21 Apr 2008 EE1309
Birth
25 Apr 2008
Death
[Death by: Unknown means]

You created this PDF from an application that is not licensed to print to novaPDF printer (http://www.novapdf.com)

```
       1310    F    21 Apr 2008     645    1046  ALMERIA    21 Apr 2008 EE1310
Birth
       1311    F    21 Apr 2008     645    1046  ALMERIA    21 Apr 2008 EE1311
Birth      PEPA TV
       1312    M    25 Apr 2008     646     588  ALMERIA    25 Apr 2008 EE1312
Birth
                                                 CABARCENO  13 Aug 2009    UNK
Transfer
                                                            29 Apr 2010
Death
                           [Death by: Unknown means]
       1313    M     2 May 2008     642    1049  ALMERIA     2 May 2008 EE1313
Birth
                                                 CABARCENO  13 Aug 2009    UNK
Transfer
                                                             2 Jul 2010
Death
                           [Death by: Unknown means]
       1314    F     2 May 2008     642    1049  ALMERIA     2 May 2008 EE1314
Birth
       1315    M     4 May 2008     646    1053  ALMERIA     4 May 2008 EE1315
Birth
                                                 CABARCENO  13 Aug 2009    UNK
Transfer
                                                             9 Jun 2010
Death
                           [Death by: Unknown means]
       1316    M     4 May 2008     646    1053  ALMERIA     4 May 2008 EE1316
Birth
                                                 CABARCENO  13 Aug 2009    UNK
Transfer
                                                             5 Dec 2009
Death
                           [Death by: Unknown means]
       1317    M     5 May 2008     646     608  ALMERIA     5 May 2008 EE1317
Birth
                                                            17 May 2008
Death
                           [Death by: Unknown means]
       1318    M    10 Feb 2008     650    1062  LA LAJITA  10 Feb 2008    UNK
Birth
                                                            14 Feb 2009
Death
                           [Death by: Unknown means]
       1319    M    14 Jun 2008     648     533  ALMERIA    14 Jun 2008 EE1319
Birth
                                                 CABARCENO  13 Aug 2009    UNK
Transfer
                                                             6 Dec 2009
Death
                           [Death by: Unknown means]
       1320    F     6 Jul 2008     645    1044  ALMERIA     6 Jul 2008 EE1320
Birth
                                                             1 Aug 2008
Death
                           [Death by: Unknown means]
       1321    F     6 Jul 2008     645    1044  ALMERIA     6 Jul 2008 EE1321
```


You created this PDF from an application that is not licensed to print to novaPDF printer (http://www.novapdf.com)

Birth

1322 M 7 Jun 2008 646 609 ALMERIA 7 Jun 2008 EE1322

Birth

CABARCENO 13 Aug 2009 UNK

Transfer

16 Dec 2009

Death

[Death by: Unknown means]

1323 F 6 Jul 2008 646 609 ALMERIA 6 Jul 2008 EE1323

Birth

14 Jul 2008

Death

[Death by: Unknown means]

1324 F 21 Mar 2007 989 1196 SANDIEGOZ 21 Mar 2007 507066

Birth

JOHNSONLA 14 May 2007 UNK

ltf

1325 F 30 Mar 2007 1028 1079 SANDIEGOZ 30 Mar 2007 507076

Birth

2 Apr 2007

Death

[Death by: Unknown means]

1326 M 30 Mar 2007 1028 1079 SANDIEGOZ 30 Mar 2007 507077

Birth

30 Apr 2007

Death

[Death by: Unknown means]

1327 M 1 Apr 2007 1028 858 SANDIEGOZ 1 Apr 2007 507080

Birth

6 Jun 2007

Death

[Death by: Unknown means]

1328 F 2 Apr 2007 989 1089 SANDIEGOZ 2 Apr 2007 507082

Birth

JOHNSONLA 14 May 2007 UNK

ltf

1329 M 2 Apr 2007 989 1089 SANDIEGOZ 2 Apr 2007 507081

Birth

JOHNSONLA 14 May 2007 UNK

ltf

1330 F 17 Apr 2007 1028 1122 SANDIEGOZ 17 Apr 2007 507094

Birth

JOHNSONLA 27 May 2007 UNK

ltf

1331 F 17 Apr 2007 1028 1122 SANDIEGOZ 17 Apr 2007 507095

Birth

17 Apr 2007

Death

[Death by: Unknown means]

1332 F 26 Apr 2007 976 1086 LANGLEY 26 Apr 2007 H07010

Birth

26 Apr 2007

Death

[Death by: Unknown means]

1333 F 19 May 2007 1028 1116 SANDIEGOZ 19 May 2007 507116

Birth

JOHNSONLA 19 Dec 2007 UNK

You created this PDF from an application that is not licensed to print to novaPDF printer (http://www.novapdf.com)

ltf

1334 M 1 Jun 2007 1028 1102 SANDIEGOZ 1 Jun 2007 507126
Birth
29 Jun 2007
Death
[Death by: Unknown means]

1335 F 9 Sep 2007 1028 813 SANDIEGOZ 9 Sep 2007 507194
Birth

1336 F 25 Mar 2008 1028 1102 SANDIEGOZ 25 Mar 2008 508030
Birth

1337 F 25 Mar 2008 1028 1102 SANDIEGOZ 25 Mar 2008 508031
Birth

1338 M 30 Mar 2008 1028 1079 SANDIEGOZ 30 Mar 2008 508032
Birth

1339 F 30 Mar 2008 1028 1079 SANDIEGOZ 30 Mar 2008 508033
Birth
3 Apr 2008
Death
[Death by: Unknown means]

1340 F 7 May 2009 1076 1155 ALMERIA 7 May 2009 EE1340
Birth

1341 F 7 May 2009 1076 1155 ALMERIA 7 May 2009 EE1341
Birth

1342 M 12 May 2009 610 1049 ALMERIA 12 May 2009 EE1342
Birth

1343 F 12 May 2009 970 1145 ALMERIA 12 May 2009 EE1343
Birth

1344 F 15 May 2009 1076 1138 ALMERIA 15 May 2009 EE1344
Birth

1345 M 15 May 2009 1076 1138 ALMERIA 15 May 2009 EE1345
Birth

1346 M 15 May 2009 1064 1224 ALMERIA 15 May 2009 EE1346
Birth
16 May 2009
Death
[Death by: Unknown means]

1347 M 18 May 2009 1064 1224 ALMERIA 18 May 2009 EE1347
Birth

1348 M 18 May 2009 1064 1051 ALMERIA 18 May 2009 EE1348
Birth
11 Dec 2012
Death
[Death by: Unknown means]

1349 F 18 May 2009 1064 1051 ALMERIA 18 May 2009 EE1349
Birth

1350 M 18 May 2009 1064 1050 ALMERIA 18 May 2009 EE1350
Birth

1351 M 18 May 2009 1064 1050 ALMERIA 18 May 2009 EE1351
Birth

1352 M 18 May 2009 1064 1239 ALMERIA 18 May 2009 EE1352

You created this PDF from an application that is not licensed to print to novaPDF printer (http://www.novapdf.com)

Birth
1353 M 18 May 2009 1076 1140 ALMERIA 18 May 2009 EE1353
Birth
19 Sep 2011
Death
[Death by: Unknown means]

1354 M 20 May 2009 970 640 ALMERIA 20 May 2009 EE1354
Birth
21 Dec 2012
Death
[Death by: Unknown means]

1355 F 20 May 2009 970 640 ALMERIA 20 May 2009 EE1355
Birth

1356 M 21 May 2009 970 1182 ALMERIA 21 May 2009 EE1356
Birth

1357 F 21 May 2009 970 1182 ALMERIA 21 May 2009 EE1357
Birth

1358 F 23 May 2009 1064 1174 ALMERIA 23 May 2009 EE1358
Birth

1359 F 23 May 2009 1064 1174 ALMERIA 23 May 2009 EE1359
Birth

1360 M 25 May 2009 970 1177 ALMERIA 25 May 2009 EE1360
Birth

1361 M 25 May 2009 970 1177 ALMERIA 25 May 2009 EE1361
Birth

1362 M 28 May 2009 1064 1235 ALMERIA 28 May 2009 EE1362
Birth

1363 F 3 Jun 2009 610 1159 ALMERIA 3 Jun 2009 EE1363
Birth

1364 F 3 Jun 2009 610 1159 ALMERIA 3 Jun 2009 EE1364
Birth
30 Jul 2009
Death
[Death by: Unknown means]

1365 M 4 Jun 2009 610 641 ALMERIA 4 Jun 2009 EE1365
Birth

1366 F 4 Jun 2009 610 641 ALMERIA 4 Jun 2009 EE1366
Birth

1367 M 22 Jun 2009 970 1163 ALMERIA 22 Jun 2009 EE1367
Birth

1368 M 15 Jul 2009 970 1230 ALMERIA 15 Jul 2009 EE1368
Birth
18 Jul 2009
Death
[Death by: Unknown means]

1369 F 9 Jul 2009 1000 1012 LA LAJITA 9 Jul 2009 UNK
Birth

1370 M 9 Jul 2009 1000 1012 LA LAJITA 9 Jul 2009 UNK
Birth

1371 M 13 Jul 2009 1000 1062 LA LAJITA 13 Jul 2009 UNK

You created this PDF from an application that is not licensed to print to novaPDF printer (http://www.novapdf.com)

Birth
1372 M 17 Jul 2009 1000 1063 LA LAJITA 17 Jul 2009 UNK
Birth
1373 M 22 Jul 2009 1000 1190 LA LAJITA 22 Jul 2009 UNK
Birth
3 May 2011
Death
[Death by: Unknown means]
1374 F 23 Jul 2009 1000 1013 LA LAJITA 23 Jul 2009 UNK
Birth
1375 M 23 Jul 2009 1000 1194 LA LAJITA 23 Jul 2009 UNK
Birth
1376 M 11 May 2010 1150 1183 ALMERIA 11 May 2010 EE1376
Birth
1377 F 12 May 2010 1052 1235 ALMERIA 12 May 2010 EE1377
Birth
1378 M 13 May 2010 1052 1069 ALMERIA 13 May 2010 EE1378
Birth
1379 M 15 May 2010 1168 1276 ALMERIA 15 May 2010 EE1379
Birth
20 Jun 2011
Death
[Death by: Unknown means]
1380 F 15 May 2010 1168 1276 ALMERIA 15 May 2010 EE1380
Birth
1381 F 17 May 2010 1150 1182 ALMERIA 17 May 2010 EE1381
Birth
19 May 2010
Death
[Death by: Unknown means]
1382 F 18 May 2010 1150 1277 ALMERIA 18 May 2010 EE1382
Birth
1383 M 18 May 2010 1168 1280 ALMERIA 18 May 2010 EE1383
Birth
1384 F 19 May 2010 1168 1051 ALMERIA 19 May 2010 EE1384
Birth
27 May 2010
Death
[Death by: Unknown means]
1385 M 19 May 2010 1168 1051 ALMERIA 19 May 2010 EE1385
Birth
27 May 2010
Death
[Death by: Unknown means]
1386 M 19 May 2010 1168 1051 ALMERIA 19 May 2010 EE1386
Birth
27 May 2010
Death
[Death by: Unknown means]
1387 F 23 May 2010 1160 1304 ALMERIA 23 May 2010 EE1387
Birth
1388 F 23 May 2010 1150 1186 ALMERIA 23 May 2010 EE1388

You created this PDF from an application that is not licensed to print to novaPDF printer (http://www.novapdf.com)

Birth

1389 M 23 May 2010 1150 1145 ALMERIA 23 May 2010 EE1389

Birth

5 Jun 2010

Death

[Death by: Unknown means]

1390 M 23 May 2010 1150 1145 ALMERIA 23 May 2010 EE1390

Birth

1391 F 23 May 2010 1168 1224 ALMERIA 23 May 2010 EE1391

Birth

1392 F 26 May 2010 1168 1274 ALMERIA 26 May 2010 EE1392

Birth

7 Jun 2010

Death

[Death by: Unknown means]

1393 F 26 May 2010 1150 1177 ALMERIA 26 May 2010 EE1393

Birth

1394 F 1 Jun 2010 1160 1138 ALMERIA 1 Jun 2010 EE1394

Birth

21 Jan 2013

Death

[Death by: Unknown means]

1395 M 7 Jun 2010 1168 1308 ALMERIA 7 Jun 2010 EE1395

Birth

1396 M 7 Jun 2010 1168 1308 ALMERIA 7 Jun 2010 EE1396

Birth

30 Jun 2012

Death

[Death by: Unknown means]

1397 F 7 Jun 2010 1160 1140 ALMERIA 7 Jun 2010 EE1397

Birth

1398 F 8 Jun 2010 1160 1141 ALMERIA 8 Jun 2010 EE1398

Birth

30 Jun 2010

Death

[Death by: Unknown means]

1399 F 13 Jun 2010 1160 1092 ALMERIA 13 Jun 2010 EE1399

Birth

1400 F 13 Jun 2010 1160 1092 ALMERIA 13 Jun 2010 EE1400

Birth

1401 F 21 Jun 2010 1052 1070 ALMERIA 21 Jun 2010 EE1401

Birth

23 Jun 2010

Death

[Death by: Unknown means]

1402 F 21 Jun 2010 1052 1070 ALMERIA 21 Jun 2010 EE1402

Birth

1403 F 22 Jun 2010 1052 588 ALMERIA 22 Jun 2010 EE1403

Birth

1404 F 22 Jun 2010 1052 588 ALMERIA 22 Jun 2010 EE1404

Birth

1405 M 30 Jun 2010 1168 1311 ALMERIA 30 Jun 2010 EE1405

You created this PDF from an application that is not licensed to print to novaPDF printer (http://www.novapdf.com)

Birth

1406 F 3 Aug 2010 1160 1306 ALMERIA 3 Aug 2010 EE1406
Birth

1407 M 15 Aug 2010 1160 1298 ALMERIA 15 Aug 2010 EE1407
Birth
19 Jan 2013
Death
[Death by: Unknown means]

1408 M 15 Aug 2010 1160 1298 ALMERIA 15 Aug 2010 EE1408
Birth
23 May 2012
Death
[Death by: Unknown means]

1409 M 24 Sep 2010 1168 1310 ALMERIA 24 Sep 2010 EE1409
Birth

1410 M 17 Jun 2011 1054 1227 ALMERIA 17 Jun 2011 EE1410
Birth
6 Aug 2011
Death
[Death by: Unknown means]

1411 M 25 Jun 2011 1054 1053 ALMERIA 25 Jun 2011 EE1411
Birth
30 Jan 2013
Death
[Death by: Unknown means]

1412 M 25 Jun 2011 1054 1053 ALMERIA 25 Jun 2011 EE1412
Birth
20 Aug 2011
Death
[Death by: Unknown means]

1413 M 11 Jul 2011 1054 1053 ALMERIA 11 Jul 2011 EE1413
Birth

1414 F 6 Jun 2011 1000 1012 LA LAJITA 6 Jun 2011 UNK
Birth

1415 F 3 Jun 2011 1000 647 LA LAJITA 3 Jun 2011 UNK
Birth

1416 F 15 Mar 2011 1000 1062 LA LAJITA 15 Mar 2011 UNK
Birth

1417 M 25 Mar 2011 1000 1013 LA LAJITA 25 Mar 2011 UNK
Birth

1418 M 4 Mar 2011 1000 1190 LA LAJITA 4 Mar 2011 UNK
Birth

1419 M 4 Mar 2011 1000 1193 LA LAJITA 4 Mar 2011 UNK
Birth

1420 F 6 Mar 2011 1000 1240 LA LAJITA 6 Mar 2011 UNK
Birth

==
===============

TOTALS: 690.715.15 (1420)

You created this PDF from an application that is not licensed to print to novaPDF printer (http://www.novapdf.com)

ALDERGROV Greater Vancouver Zoo
5048 - 264th St., Aldergrove, British Columbia, Canada, V4W 1N7
(604)856-6825 fax: (604)857-9008 jdorgan@greatervancouverzoo.com

ALMER.CTY CASSINELLO
CORTIJO SAN INDALECIO,, Almeria, Spain, European Region, 04009

ALMERIA Parque de Rescate de Fauna Sahariana
Estacion Exper. de Zonas-Aridas, Almeria, Spain, E-04001
34 950 281045 fax: 34 950 277100 gerardo@eeza.csic.es

BOUKORNIN _______________________________

BULVERDE NBJ Ranch (Buddy Jordan)
4580 Farm Road 1863, Bulverde, Texas, USA, 78163-2439
(512)653-3673

CABARCENO Parque De La Naturaleza De Cabarceno
Cantur, S.A., Santander, Cantabria, Spain, 39004
34 942 56 3736

CINCINNAT Cincinnati Zoo & Botanical Garden
3400 Vine St., Cincinnati, Ohio, USA, 45220-1399
(513)569-8225 fax: (513)569-8213 mary.noell@cincinnatizoo.org
Contact: Ms. Mary Noell

EDWARDS Valley Game Farm (Donald Pyne)
Rural Route 2, Edwards, Ontario, Canada

ESTEPONA Parque De La Naturaleza Selwo (GRPR)
Autovia decCadiz Malaga km.162,5, Estepona, Malaga, Spain, 29680
34 952 79 2150 fax: 34 952 79 6532 estepona@mail.ddnet.es

EVANSVLLE Mesker Park Zoo
2421 Bement Ave., Evansville, Indiana, USA, 47720
(812)435-6143x405 fax: (812)422-9673 sdawson@meskerparkzoo.com
Contact: Ms. Sydney Dawson

FOUTS J Tanganyika Wildlife Co. (Jim Fouts)
PO Box 12084, Wichita, Kansas, USA, 67277
(316)794-8954

HOGLE Utah's Hogle Zoo
Utah Zoological Society, Salt Lake City, Utah, USA, 84108
(801)584-1740 fax: (801)584-1770 dawnm@hoglezoo.org
Contact: Ms. Dawn Ross

HOLIDAY Earl Tatum
5 Pleasant Ridge Dr., Eureka Springs, Arkansas, USA, 72632
(501)253-9696

JOHNSONLA Safari Enterprises (Larry Johnson)
5421 Berryhill Dr., Yorba Linda, California, USA, 92686
(714)978-7742

L RUHE Louis Ruhe GmbH (1860-1995)
Gerdag Strasse 8, 3220 Alfeld/leine, Germany

LA LAJITA Camel Safari and Zoo
(Oasis de Los Camellos), La Lajita, Fuerteven, Canary Islands,
Spain

LANGLEY Mountain View Conservation & Breeding
8011 - 240th Street, Langley, British Columbia, Canada, V1M 3P9
(604)881-1220 fax: (604)881-1221 gblankstein@mtnviewfarms.com
Contact: Mr. Sean Mac Connell

LUGO LUGO

You created this PDF from an application that is not licensed to print to novaPDF printer (http://www.novapdf.com)

Spain, Cont Europe, European Region

MADRID Z Zoo Aquarium Madrid (GRPR)
Casa de Campo, s/n, Madrid, Spain, E-28011
34 91 512 3770 fax: 34 91 711 8163 comzoo@mail.ddnet.es
Contact: Dr. Enrique Saez Fernandez

MARRAKECH MARRAKECH
Morocco, North Africa, African Region

MEMPHIS Memphis Zoological Garden & Aquarium
2000 Prentiss Place, Memphis, Tennessee, USA, 38112
(901)725-3400x3119 fax: (901)725-9305 knewland@memphiszoo.org
Contact: Mr. Dietrich Schaaf

MUNICH Munchener Tierpark Hellabrunn
Tierparkstrasse 30, Muenchen, Germany, D-81543
49 89 62 50817 fax: 49 89 62 50852 rau@zoo-munich.de
Contact: Ms. Beatrix Rau

OR WILDLF Oregon Wildlife Foundation
8375 Steel Bridge Rd., Sheridan, Oregon, USA, 97378
(503)876-2988
Contact: Ms. Nancy Ayotte

PALM DES The Living Desert Zoo and Gardens
47-900 Portola Ave., Palm Desert, California, USA, 92260
(760)346-5694x275 fax: (760)346-9070
afletcherjones@livingdesert.org
Contact: Ms. Anne Fletcher-jones

PEACE RV Peace River Ctr. Cons.Trop.Ungulates
4300 Southwest County Rd 769, Arcadia, Florida, USA, 34266
(863)993-4529
Contact: Ms. Sherry Norris

PROVIDNCE Roger Williams Park Zoo
1000 Elmwood Ave., Providence, Rhode Island, USA, 02907-3600
(401)785-3510x310 fax: (401)941-3988 happyhopping@rwpzoo.org
Contact: Ms. Adrienne Miller

RABAT Parc Zoologique Natl. De Rabat-Morocco
B.P. 41 42, Temara, Rabat, Morocco, 12 000
212 7 741259

SANDIEGOZ Zoological Society of San Diego
PO Box 120551, San Diego, California, USA, 92112-0551
(619)685-3250 fax: (619)231-6572 tgiezendan@sandiegozoo.org
Contact: Ms. Toni Giezendanner

SD-WAP San Diego Wild Animal Park
15500 San Pasqual Valley Rd, Escondido, California, USA, 92027
(619)685-3250 fax: (760)231-6572 tgiezendan@sandiegozoo.org
Contact: Ms. Toni Giezendanner

SHADOW Shadow Nursery Inc. (Don Shadow)
254 Shadow Nursery Road, Winchester, Tennessee, USA, 37398
(931)967-6059

ST LOUIS Saint Louis Zoological Park
1 Government Dr., St. Louis, Missouri, USA, 63110-1395
(314)781-0900x372 fax: (314)647-7969 haliday@stlzoo.org
Contact: Ms. Rae Lynn Haliday

TABERNAS Reserva Zool Del Desierto de Tabernas
Ctra.Nacional 340, Km 364, Desierto De Tabernas, Almeria, Spain,
04200
34 950 36 2931

UARAB EM UNION OF ARAB EMIRATES
Southwestern Asia, Asian Region

W.AFRICA WEST AFRICA
African Region

WILDS The Wilds
14000 International Rd., Cumberland, Ohio, USA, 43732
(740)638-2072 fax: (740)638-2287 pglaze@thewilds.org

You created this PDF from an application that is not licensed to print to novaPDF printer (http://www.novapdf.com)

stdk cuvier 01-01-2012
Contact: Ms. Patty Glaze

Total number of institutions: 35

Studbook Legend

Stud#: Animal's permanent studbook number. Studbook numbers may not always be assigned chronologically, due to animals being recorded as they become known to the Studbook Keeper.

Sex: M for male, m for castrated male, F for female, f for contracepted/neutered female, U for unknown sex

Birth Date: Animal's birth date. A tilde mark, '~', before a date indicates that it is approximate. '??' indicates that the date is unknown.

Sire and Dam: Studbook numbers for the animal's sire and dam. 'UNK' if unknown. 'MULT' if several candidates have been identified. 'WILD' if the sire or dam was considered to be wild - this animal is a founder.

Location: The institution holding the animal at the time. If the animal is no longer traceable by the Studbook Keeper, 'ltf' (lost-to-followup) will appear to the right of the last known location.

Date: The date on which the event occurred. A tilde mark, '~', before a date indicates that it is approximate. '??' indicates that the date is unknown.

Local ID: The identification assigned by the specimen's location, often its ISIS number, house number, or occasionally house name.

Event: Capture, Birth, Transfer, Loan, Ownership change, Death or Release

Identifiers: House Name, Transponder, Tag/Band, Tattoo, Notch

You created this PDF from an application that is not licensed to print to novaPDF printer (http://www.novapdf.com)

♀→

You created this PDF from an application that is not licensed to print to novaPDF printer (http://www.novapdf.com)
