# Supplementary material for: Better Fitness in Captive Cuvier’s Gazelle despite Inbreeding Increase: Evidence of Purging?
Source: PLoS One. 2015 Dec 17;10(12):e0145111. doi: 10.1371/journal.pone.0145111 (PMC4682998; doi:10.1371/journal.pone.0145111)
Supplement: S2 File — PERIOD indicates whether individuals (ID) were born before (P1) or after (P2) the pairing strategy changes in 2006. (PDF) [file pone.0145111.s002.pdf]

Supporting Information

S2 File: Genotypes for the seven microsatellite markers used to estimate genetic diversity in Cuvier's gazelle. PERIOD indicates whether individuals (ID) were born before (P1) or after (P2) the pairing strategy changes in 2006.

Period	ID	CSPS115	CSPS115	OarFCB193	OarFCB193	CelJP15	CelJP15	CSSM22	CSSM22	ETH225	ETH225	OarFCB304	OarFCB304	MM12	MM12
P1	GC125	258	262	118	124	169	169	222	240	0	0	140	140	180	182
P1	GC154	256	262	124	144	169	169	0	0	0	0	138	140	180	180
P1	GC159	262	262	118	144	169	169	224	240	0	0	130	140	180	180
P1	GC253	256	258	108	116	169	169	224	226	150	164	144	152	180	182
P1	GC270	256	262	116	120	169	175	226	240	142	150	140	152	182	182
P1	GC323	260	262	116	124	169	169	222	240	0	0	140	152	180	182
P1	GC335	258	262	118	124	169	169	224	224	150	150	152	152	180	182
P1	GC349	256	258	120	124	169	175	222	224	0	0	140	152	180	182
P1	GC370	260	260	118	118	169	169	224	240	0	0	140	150	180	182
P1	GC397	256	262	120	144	175	175	226	226	0	0	0	0	180	182
P1	GC399	256	262	116	118	169	169	226	240	0	0	140	152	180	180
P1	GC546	256	260	116	116	0	0	224	226	148	150	140	152	182	182
P1	GC569	256	262	124	144	169	175	224	226	142	150	140	152	180	182
P1	GC571	256	256	120	124	169	169	222	222	0	0	144	152	182	182
P1	GC581	256	262	108	116	169	171	224	224	148	150	152	152	182	182
P1	GC592	256	258	118	124	169	169	222	224	148	150	140	144	180	182
P1	GC611	256	256	116	118	169	171	226	226	0	0	140	152	180	182
P1	GC624	256	258	118	124	169	169	224	226	0	0	144	152	180	182
P1	GC628	256	262	120	144	169	169	226	240	150	158	140	152	180	180
P1	GC630	256	256	118	144	169	169	224	240	150	150	140	152	180	182
P1	GC633	256	256	116	116	169	169	226	226	148	150	144	152	180	182
P1	GC1001	256	260	118	144	169	169	224	226	146	150	144	152	180	182
P1	GC1067	256	262	116	116	169	169	226	240	0	0	144	152	182	182
P1	GC1068	260	262	118	144	169	171	226	240	0	0	152	152	180	182

P1	GC1076	262	262	116	124	169	169	224	224	0	0	126	142	182	182
P1	GC1093	252	258	116	116	169	171	226	240	0	0	140	152	180	182
P1	GC1136	256	262	116	116	169	169	222	222	0	0	140	144	180	182
P1	GC1166	256	260	108	116	169	169	222	224	0	0	140	152	180	182
P1	GC997	260	262	116	116	169	171	226	240	148	150	140	152	180	182
P2	GC1382	256	258	108	144	169	169	224	224	0	0	142	152	182	182
P2	GC1386	260	262	118	124	169	169	222	226	0	0	152	152	180	182
P2	GC1395	262	262	116	124	169	169	222	226	0	0	144	152	182	182
P2	GC1397	256	262	116	120	171	171	226	226	158	160	140	152	180	182
P2	GC1399	252	252	116	124	169	171	224	226	164	164	144	144	180	182
P2	GC1404	256	260	116	118	169	169	224	224	160	160	152	152	180	182
P2	GC1405	262	262	116	144	169	169	224	226	142	158	140	152	182	182
P2	GC1406	256	256	124	124	169	171	226	226	148	158	140	144	180	182
P2	GC1408	262	262	116	118	169	171	224	226	0	0	144	152	180	182
P2	GC1409	262	262	116	116	169	169	222	226	0	0	144	152	182	182
